# Positioning Imatinib for Pulmonary Arterial Hypertension: A Dose-Finding Phase 2 Study

**DOI:** 10.1164/rccm.202410-1929OC

**Published:** 2025-03-13

**Authors:** Alexander M. K. Rothman, Sofia S. Villar, Jennifer Middleton, Andreas A. Roussakis, Frances Varian, Hamza Zafar, Martin Law, Jane Apperley, Imke H. Bartelink, Medhat M. Said, Juan A. Delgado-SanMartin, David G. Kiely, Luke Howard, Mark Toshner, S. John Wort, Martin R. Wilkins

**Affiliations:** ^1^ Sheffield Pulmonary Vascular Disease Unit, Royal Hallamshire Hospital, Sheffield Teaching Hospitals NHS Foundation Trust, Sheffield, United Kingdom;; ^2^ Department of Clinical Medicine, The University of Sheffield, Sheffield, United Kingdom;; ^3^ MRC Biostatistics Unit and; ^4^ VPD Heart & Lung Research Institute, University of Cambridge, Cambridge, United Kingdom;; ^5^ Papworth Trials Unit Collaboration, Royal Papworth Hospital NHS Foundation Trust, Cambridge, United Kingdom;; ^6^ National Heart and Lung Institute and; ^7^ Centre for Haematology, Imperial College London, London, United Kingdom;; ^8^ Department of Clinical Pharmacology and Pharmacy, Amsterdam University Medical Center, Amsterdam, the Netherlands;; ^9^ Cancer Center Amsterdam, Amsterdam, the Netherlands;; ^10^ National Pulmonary Hypertension Service, Hammersmith Hospital, London, United Kingdom; and; ^11^ National Pulmonary Hypertension Service, Royal Brompton Hospital, London, United Kingdom

**Keywords:** implanted hemodynamic sensors, remote monitoring, adaptive trial design

## Abstract

**Rationale:**

Imatinib, 400 mg daily, reduces pulmonary vascular resistance and improves exercise capacity in patients with pulmonary arterial hypertension. Concerns about safety and tolerability limit its use.

**Objectives:**

We sought to identify a safe and tolerated dose of oral imatinib between 100 mg and 400 mg daily and evaluate its efficacy.

**Methods:**

Oral imatinib was added to the background therapy of 17 patients with pulmonary arterial hypertension, including 13 who were implanted with devices that provide daily measurements of cardiopulmonary hemodynamics and physical activity. The first patient was started on 100 mg daily. The next 12 patients, recruited serially, were started on 200 mg, 300 mg, or 400 mg daily, following a continuous reassessment dose-finding model. An extension cohort (Patients 14–17) received 100 mg or 200 mg daily.

**Measurements and Main Results:**

The continuous reassessment model recommended starting dose was 200 mg daily. The most common side effect was nausea. Imatinib reduced mean pulmonary artery pressure (−6.5 mm Hg; 95% confidence interval [CI] = −2.4 to −10.6; *P* < 0.01) and total pulmonary resistance (−2.8 Wood units; 95% CI = −1.5 to −4.2; *P* < 0.001), with no significant change in cardiac output. The reduction in total pulmonary resistance was dose and exposure dependent; the reduction from baseline with imatinib, at 200 mg daily, was −20.3% (95% CI = −14.3 to −26.3%). Total pulmonary resistance and night heart rate declined steadily over the first 28 days of treatment and remained below baseline up to 40 days after imatinib withdrawal.

**Conclusions:**

Oral imatinib, 200 mg daily, is well tolerated as an add-on treatment for pulmonary arterial hypertension. A delay in the return of cardiopulmonary hemodynamics to baseline was observed after imatinib was stopped.

**At a Glance Commentary**

**Scientific Knowledge on the Subject**

Oral imatinib has been shown to improve cardiopulmonary hemodynamics and exercise capacity in patients with pulmonary arterial hypertension (PAH) but is poorly tolerated at doses of 400 mg daily. It is not licensed for the treatment of PAH.

**What This Study Adds to the Field**

Oral imatinib, 200 mg daily, is well tolerated and reduces total pulmonary resistance in patients with PAH when added to background licensed therapies. Stopping imatinib results in a return of total pulmonary resistance to levels measured before commencing imatinib, but there is a temporal delay of up to 40 days. This property of imatinib may lend itself to an intermittent or pulsed dosing regimen, but this remains to be demonstrated.

Pulmonary arterial hypertension (PAH) is a rare condition in which pulmonary artery pressure (PAP) is elevated by increased resistance to blood flow through precapillary pulmonary vessels (1, 2). Patients die prematurely of right heart failure. Histological analysis shows marked structural remodeling of pulmonary arterioles with occlusion of the lumen (1). Currently licensed treatments improve symptoms, but none, as yet, have been shown to address the structural remodeling of arterioles (3) or satisfy other disease-modifying criteria (4).

Imatinib was the first treatment to be investigated in PAH with the primary aim of targeting directly vascular remodeling (5, 6). In addition to Bcr-Abl, imatinib protein targets include platelet-derived growth factor (PDGF) and c-kit, two trophic factors elevated in the lungs of patients with PAH (7, 8). A Phase-3 study (the Imatinib in Pulmonary Arterial Hypertension, a Randomized Efficacy Study, or the IMPRES) (6) of 202 patients showed that imatinib, at 400 mg daily, reduces mean PAP and pulmonary vascular resistance (PVR) and increases 6-minute walk distance in patients who are able to tolerate the drug, but only 43% continued the drug for 6 months. Additionally, 8 patients, who were also treated with an anticoagulant, developed a subdural hematoma during the main and extension studies (6). The high number of dropouts and concerns about safety prevented regulatory approval. Recognition, such as in the first case report (9), that some patients respond well and that there is an unmet clinical need in PAH has led some experienced centers to consider the use of imatinib off license (10, 11).

Against this background, we conducted an open-label, single-arm, adaptive design study using a Bayesian continuous reassessment model (CRM) (12) to explore the tolerability and safety of once-daily (q.d.) doses of imatinib between 100 mg and 400 mg when added to background treatment (13). We obtained daily home measurements of cardiopulmonary hemodynamics and physical activity from a subset of patients to explore efficacy. Patient genotype at the *PDGFRB* locus (rs2304058) and plasma imatinib levels were used to understand the variability in the response to the drug.

## Methods

### Trial Design and Participants

Patients with PAH were recruited from four National Pulmonary Hypertension referral centers in the United Kingdom (Royal Hallamshire Hospital, Sheffield; Hammersmith Hospital, London; Royal Brompton Hospital, London; and Royal Papworth Hospital, Cambridge). All patients were stable on their existing licensed therapy for at least 1 month before screening. None were taking anticoagulants. Imatinib—100, 200, 300, and 400 mg q.d.—was added to existing treatment and continued for up to 24 weeks. The first patient was started on imatinib at 100 mg q.d. Safety and tolerability data collected during the subsequent 4 weeks was used to update the CRM. Patients 2–13 were recruited serially at a minimum of 4-week intervals, with the starting dose for each patient recommended by the continuously updated CRM. Patients 14–17 were recruited to an extension cohort that was designed to collect additional data to assess the tolerability of 100 mg and 200 mg as a starting dose. The full protocol has been published previously (13). The code for the CRM-based dose-finding and interim analysis for the study is available at: https://github.com/MRCBSU/pipah-wilkins.

### Trial Oversight and Timelines

The trial was designed by the authors and sponsored by the U.K. National Institute for Health and Care Research. The trial protocol was approved by the U.K. Health Research Authority (Research Ethics Committee reference 10/SC/0240) and is registered on ClinicalTrials.gov (NCT 04416750). All the participants provided written informed consent.

The protocol was approved on July 28, 2020. The trial was planned initially in two stages, with Stage 1 focused on safety and tolerability and Stage 2 focused on efficacy. However, the coronavirus disease (COVID-19) pandemic prompted an amendment to the study protocol in October 2020 to permit enrollment of patients who had been implanted with a CardioMEMS PAP monitor (Abbott; *see* Figure E1 in the online supplement) and a LinQ insertable cardiac monitor (Medtronic) to enable remote monitoring and follow-up (with the Cordella Heart Failure System; Endotronix) under an established research ethics protocol: Feasibility of Novel Clinical Trial Infrastructure, Design and Technology for Early Phase Studies in Patients with Pulmonary Hypertension (or, the FIT-PH study; ClinicalTrials.gov ID: NCT04078243, REC 19/YH/0354). This enabled the collection of efficacy data in patients with implanted devices, and the statistical analysis plan was updated to include the analysis of longitudinal data from these patients. The first patient entered the study on January 26, 2021.

The trial was conducted in accordance with the International Council for Harmonization guidelines for Good Clinical Practice and the principles of the Declaration of Helsinki. The starting dose for Patients 2–17 was considered and approved by an independent data monitoring (and ethics) committee, informed by the CRM. A separate independent trial steering committee met regularly to review the overall conduct of the study. The authors were responsible for the collection, analysis, and interpretation of the data.

### Endpoint and Assessments

The primary endpoint was a binary variable; discontinuation of the drug for more than 5 consecutive days in the first 4 weeks after starting treatment because of adverse events that were Grade 2 or higher, as defined by the National Cancer Institute Common Terminology Criteria for Adverse Events (Version 5.0, November 2017).

Predefined secondary endpoints were the change from baseline to 24 weeks in *1*) 6-minute walk distance, a measure of functional capacity; *2*) right ventricular ejection fraction by echocardiogram; *3*) plasma N-terminal pro–B-type natriuretic peptide (NT-proBNP) levels; *4*) quality of life, as determined with EmPHasis-10 questionnaire scores; and *5*) PVR, according to genes that regulate PDGF activity.

### Treatment Period

The primary safety endpoint was tolerability at 4 weeks, with an intended imatinib treatment duration of 24 weeks. Dose adjustment, including early cessation, was permitted in patients experiencing adverse events. To ensure safety, a protocolized approach was applied at drug withdrawal, with follow-up visits at 1 and 3 months, including clinical examination, to monitor World Health Organization (WHO) functional class, NTproBNP level, and noninvasive imaging. After withdrawal, remote physiology was monitored weekly under the FIT-PH study’s ethical approval, with accelerated clinical visits for patients in whom a 10% increase in mean PAP was observed. Reinitiation of imatinib was approved by the Sheffield Teaching Hospitals NHS Foundation Trust Medicines Safety Committee for patients who showed an improvement in remote hemodynamics, NTproBNP, and WHO functional class with drug initiation, showed worsening on imatinib withdrawal, and wanted to continue on the drug.

### Genotyping

The rs2304058 gene variant was typed by 5′-nuclease real-time PCR chemistry by https://patentimages.storage.googleapis.com/e6/c2/36/e1fc24e6588746/US10093965.pdf.

### Imatinib Assay

Imatinib plasma concentrations were determined using validated methods (14, 15). We performed pharmacokinetic (PK) analysis using a previously developed imatinib PK model and nonlinear mixed-effects modeling (NONMEM, Version 7.3) (15).

### Statistical Analysis

A statistical design, the CRM, was used to identify and dose patients sequentially at the highest dose of imatinib with a ⩽20% likelihood of drug discontinuation. The initial dose/toxicity skeleton for the CRM was generated on the basis of a one parameter power model in the form, p(tox|d)=d^α^, where d is the standardized dose and our single parameter α follows a γ (1,1) distribution. The initial expected dose-toxicity curve combined expert opinion with data from the IMPRES trial (6). Simulations (13) suggested that a minimum of 13 patients were sufficient to achieve a recommended dose within 10% of the target dose, 90% of the time (*see* Statistical Analysis Plan in the online supplement). R statistical software (16) was used to update the CRM.

The efficacy endpoint, total pulmonary resistance (TPR), was calculated from invasive right heart catheterization (snapshot) or PAP monitor–derived measures as mean PAP/cardiac output. Data from remote monitoring are represented as the mean and 95% CIs of individual patient baseline adjusted 3-day average. Change in baseline and endpoint efficacy measures were compared by using paired Student’s *t* test or Wilcoxon test, as appropriate. Time to stability of TPR after drug initiation and withdrawal was evaluated using time-to-event analysis, with an event defined as a change in 3-day average of TPR of <0.15 Wood units for 6 of 7 consecutive days. We conducted the analysis by using SPSS for MacOS (29.0.2.0) and Prism for MacOS (10.2.2).

## Results

### Participants

**Figure 1.**
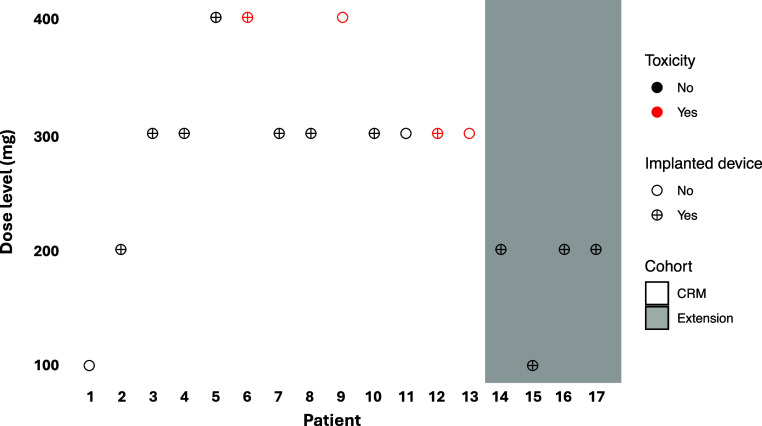
Continuous reassessment model (CRM) method. Trajectory of dose and tolerability data at 4 weeks during recruitment using the CRM (Patients 1–13) in the trial protocol and the 4-patient extension (Patients 14–17; shaded area). Red symbols represent dose-limiting adverse events; cross symbols represent patients with implanted devices.

Seventeen patients were recruited from January 2021 to February 2023 (Table 1; *see* Figures E2 and E3). All were diagnosed and managed according to European guidelines (17). Thirteen patients (9 in the CRM and 4 in the extension cohort) had implanted monitoring devices (18, 19), with a mean time from device implantation of 8.6 months (SD = 8.5). The sequence of dosing after the recommendations from the CRM is shown in Figure 1.

**Table 1.** Patient Demographics at Baseline

Characteristic	PIPAH, All (*n* = 17)	Remote Monitoring, with Devices (*n* = 13)	Baseline and Endpoint, without Devices (*n* = 4)
Gender, F:M	10F:7M	8F:5M	2F:2M
Age, yr, mean	48	53	31
BMI, kg/m^2^, mean	25	26	24
Ethnicity	15WB:1WO: 1AP	11WB: 1WO: 1AP	4WB: 0WO: 0AP
PAH classification			
Idiopathic	14	10	4
Heritable (BMPR2 mutation)	2 (1)	2 (1)	0
Associated with connective tissue disease	1	1	0
WHO functional class			
II	6	6	0
III	11	7	4
6MWD, m, mean (SD)	374 (159)	336 (156)	497 (96)
Borg Dyspnea Index, median (range)	3 (0–10)	3 (0–10)	3 (3–5)
NTproBNP, ng/L, mean (SD)	985 (1,357)	887 (1,240)	1,278 (1,848)
SBP, mm Hg, mean, (SD)	122.6 (14.4)	124.0 (16.0)	118.0 (6.0)
DBP, mm Hg, mean, (SD)	70.6 (8.7)	72.2 (8.9)	65.5 (6.8)
PVR, Wood units, mean (SD)	9.5 (3.4)	9.6 (3.0)	9.1 (5.2)
TPR, Wood units, mean (SD)	11.7 (4.0)	11.9 (3.5)	11.2 (6.0)
mPAP, mm Hg	52.9	55.8	53.6
mRAP, mm Hg	9.4	9.2	10.0
mPAWP, mm Hg, mean (SD)	10.3 (2.4)	10.2 (2.6)	10.8 (1.9)
Cardiac output, L/min, mean (SD)	4.88 (1.4)	4.74 (1.4)	5.35 (1.5)
Cardiac index, L/min/m^2^, mean (SD)	2.71 (0.76)	2.57 (0.70)	3.15 (1.02)
Treatment (before IMP)			
Dual therapy	6	6	0
Triple therapy	11	7	4
Intravenous prostacyclin	4	2	2
Calcium channel blocker	3	3	0

*Definition of abbreviations*: 6MWD = 6-minute walk distance; AP = Asian Pakistani; BMI = body mass index; DBP = diastolic blood pressure; F = female; M = male; mPAP = mean pulmonary artery pressure; mPAWP = mean pulmonary arterial wedge pressure; mRAP = mean right atrial pressure; NTproBNP = N-terminal pro–B-type natriuretic peptide; PAH = pulmonary arterial hypertension; PVR = pulmonary vascular resistance; TPR = total pulmonary resistance; SBP = systolic blood pressure; WB = White British; WO = White Other; WHO = World Health Organization.

PVR was calculated by imputation of mPAWP for instrumented patients.

### Tolerability

The primary endpoint, dose-limiting toxicity at 4 weeks follow-up, was met by 4 subjects; 2 who were started with 400 mg q.d. and 2 who were started with 300 mg q.d. (Figure E2).

At the point when Patient 13 had passed Week 4 of treatment, the CRM, driven only by tolerability, recommended 200 mg as the dose closest to the 20% tolerability target (mean and median posterior probabilities of nontolerability at 4 wk were 0.196 and 0.168, respectively). Recognizing the study had accumulated limited data in the range of 100 to 200 mg, we recruited 4 additional patients (Patients 14–17) as an extension cohort, and they were started on 100 mg or 200 mg daily to explore the lower dose range. All 4 patients tolerated their starting dose.

Overall, 14 of the 17 patients continued treatment for 6 months. Two patients (one on 400 mg daily at 18 wk and one on 300 mg daily in Week 5, respectively) withdrew during the coronavirus disease (COVID-19) pandemic for family reasons, and one patient (on 100 mg daily) withdrew at 9 weeks because of a deterioration in their PAH (requiring lung transplantation). The most common side effect was nausea. Adverse events are summarized in Table 2.

**Table 2.** Summary of Treatment-Emergent Reported Adverse Events up to 24 Weeks

Reported Adverse Event	Imatinib Patients, Various Doses (*n* = 17)		
	Any Grade	Grade ⩾2	Grade ⩾3
Nausea	9	1	0
Headache	8	2	0
Fatigue	7	2	0
Lymphocyte count decreased	5	3	0
Vomiting	5	0	0
Dyspnea	4	2	2
Dizziness	4	1	0
Periorbital edema	4	1	0
Hyperuricemia	4	0	0
Platelet count decreased	4	0	0
Neutrophil count decreased	3	2	1
Bloating	3	2	0
Bruising	3	1	0
Edema	3	0	0
Anemia	2	0	0
Alkaline phosphatase, increased	2	0	0
Arthralgia	2	0	0
Blood lactate hydrogenase, increased	2	0	0
Blurred vision	2	0	0
Dyspepsia	2	0	0
Hypokalemia	2	0	0
Lung infection	2	2	0
Noncardiac chest pain	2	1	0
Palpitations	2	0	0
Paraesthesia	2	0	0
Skin infection	2	1	0

*Definition of abbreviation*: AEs = adverse events.

Reported in *n* ⩾ 2 patients, coded using the Common Terminology Criteria for Adverse Events, Volume 5 (November 2017). Grade 1 = mild; asymptomatic or mild symptoms; clinical or diagnostic observations only; intervention not required. Grade 2 = moderate; minimal, local, or noninvasive intervention indicated; limiting age-appropriate activities of daily living. Grade 3 = severe or medically significant but not immediately life threatening; hospitalization; disabling; limiting self-care.

The final recommended starting dose of oral imatinib, which is based on the CRM for Patient 13, was 200 mg q.d. Eight patients ended the study on this dose.

### Hemodynamic Response and Physical Activity

**Figure 2.**
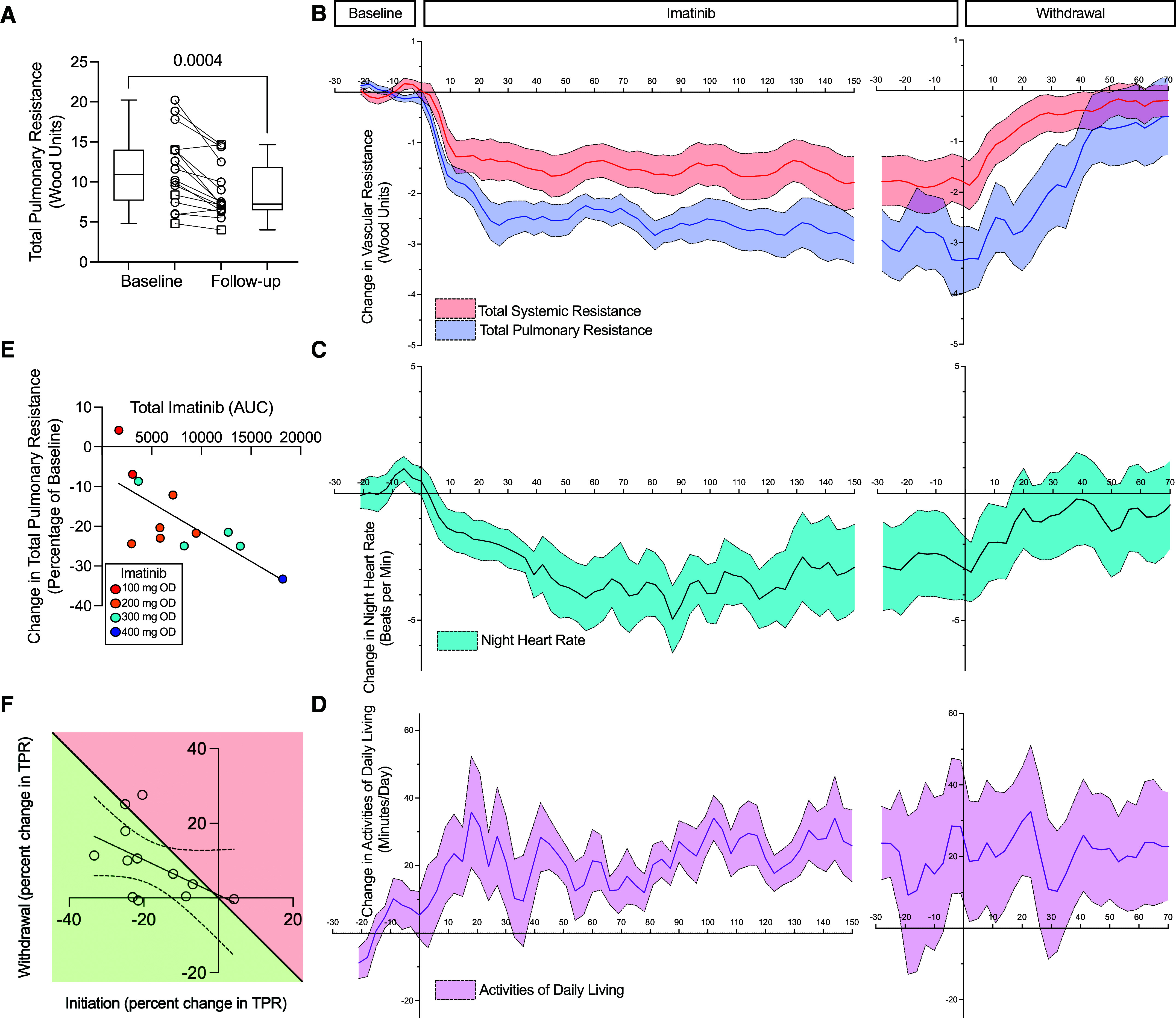
Change in total pulmonary resistance (TPR), resting heart rate, and physical activity. (*A*) Change in TPR from baseline to end of treatment with imatinib (*n* = 16). Circles represent CardioMEMS data; squares represent right heart catheter data; box and whiskers represent median and interquartile range. (*B–D*) Time course of change in (*B*) TPR (dark blue) and total systemic resistance (red), (*C*) night heart rate (light blue), and (*D*) physical activity (mauve) from baseline (period before drug administration) starting at Day 0, plotted as a rolling 3-day average in all patients with implanted devices (*n* = 13; all doses grouped response, mean ± 95% confidence interval). (*E*) Percent change in TPR from baseline at 60 days in relation to plasma level (area under the curve in μg · h/L) of imatinib at steady state. Red represents100 mg once daily (q.d.), orange represents 200 mg q.d., cyan represents 300 mg q.d., and blue represents 400 mg q.d. (*F*) Percent change in TPR 60 days after imatinib withdrawal in patients with devices (*n* = 12; one patient withdrew before plasma levels were obtained). TPR in patients represented by the green shaded area had not returned to baseline 60 days after stopping imatinib.

Follow-up hemodynamic measurements that were available for 16 patients showed a mean reduction in TPR of −2.8 Wood units (95% CI = −1.5 to −4.2; *P* = 0.0004; Figure 2A and Table 3), accompanied by reductions in plasma NTproBNP (−239 ng/L; for geometric mean ratio, 95% CI = 0.57–0.89; *P* = 0.005) and improvement in functional class (Table 3). There were no statistically significant changes in 6-minute walk distance or EmPHasis-10 scores at 24 weeks (Table 3).

**Table 3.** Change in Hemodynamic, Functional and Patient Experience Assessments from Baseline to Follow-up

Measurement	Baseline	Follow-up	Change	95% CI		*P* Value
Mean pulmonary artery pressure, mm Hg, mean (SD)	54.3 (12.7)	47.8 (11.1)	−6.5	−10.6 to −2.4		0.004
Cardiac output, L/min, mean (SD)	5.2 (1.5)	5.5 (1.4)	0.3	−0.3 to 0.9		0.28
Daytime resting heart rate, bpm, mean (SD)	73 (8.6)	68.7 (7.8)	−4.3	−7.7 to −0.9		0.02
Systemic systolic blood pressure, mm Hg, mean (SD)	116.7 (16.9)	110.2 (18.5)	−6.5	−10.3 to −2.7		0.002
Systemic diastolic blood pressure, mm Hg, mean (SD)	67 (14.7)	67.2 (18)	0.2	−5.5 to 5.8		0.948
Mean arterial pressure, mm Hg, mean (SD)	83.6 (14)	82.5 (17.8)	−1.1	−5.6 to 3.5		0.626
Total pulmonary resistance, Wood units, mean (SD)	11.6 (4.7)	8.8 (3.4)	−2.8	−4.2 to −1.5		<0.001
Total systemic resistance, Wood units, mean (SD)	16.9 (4.6)	15.5 (4.2)	−1.4	−2.8 to −0.1		0.04
Total pulmonary resistance/total systemic resistance, mean (SD)	0.7 (0.2)	0.6 (0.2)	−0.1	−0.2 to −0.1		<0.001
Six-minute walk distance, m, mean (SD)	361 (162)	386 (166)	25	−8 to 58		0.12
NTproBNP, ng/L, mean (SD)	935.7 (1330)	696.5 (1095)	0.71*	0.57 to 0.89		0.005
EmPHasis-10, mean, SD	20.7 (10.1)	17.5 (12.2)	−3.2	−7.1 to 0.8		0.1
WHO functional class, *n*, (%)						
I	0 (0)	0 (0)	—	—		0.029
II	6 (35.3)	13 (76.5)	—	—		—
III	11 (64.7)	3 (17.6)	—	—	—	—
IV	0 (0)	1 (5.9)	—	—	—	—

*Definition of abbreviations*: bpm = beats per minute; CI = confidence interval; NTproBNP = N-terminal pro–B-type natriuretic peptide; q.d. = once daily; WHO = World Health Organization.

*n* = 16 patients taking 100–400 mg q.d. Follow-up measurements are the last available measurement up to 24 weeks on treatment. One patient without implanted devices on 100 mg q.d. (who withdrew at Week 9 for lung transplantation) was excluded because of no follow-up data.

* Geometric mean ratio.

Daily remote measurements were available on 13 patients with implanted devices (Figures 2B–2D). In the 3 months before the initiation of imatinib, there were no changes in PAH therapy. After starting imatinib, TPR, total systemic resistance (TSR), and night heart rate decreased, and physical activity increased. The reduction in TPR was gradual over the first 28 days (95% CI = 9–47), with a mean decrease of −2.4 Wood units (95% CI = −1.7 to −3.1; *P* = 0.0001), and thereafter slowed (Figure 2B). The change in TPR was driven by a reduction in mean PAP with no deterioration in cardiac output (Table 3 and *see* Figure E4). It was accompanied by a reduction in resting (night) heart rate of −2.7 beats per minute (95% CI = −1.6 to −3.9; *P* = 0.0001; Figure 2C) and an increase in physical activity of 14.3 minutes (activities of daily living: 95% CI = 2.6–25.8; *P* = 0.02; Figure 2D). The reduction in TPR was dose and exposure dependent (Figure 2E). A mean difference of −20.3% (95% CI = −14.4 to −26.3) reduction in TPR from baseline was seen with 200 mg q.d. (Figure 2E), the tolerated dose predicted from the CRM. Consistent with these findings, echocardiographic markers of cardiac response, such as right atrial area and tricuspid annular plane systolic excursion (or, TAPSE), were directionally improved (*see* Table E1).

TPR and TSR increased after withdrawal of imatinib, but the increase was gradual, more so for TPR. The mean reduction in TPR on withdrawal of imatinib was −3.3 (95% CI = −2.5 to −4.1) Wood units and was still −2.6 (95% CI = −1.7 to −3.3) Wood units 15 days after stopping the drug. The rise in TPR stabilized at 47 days (95% CI = 37–58) and was −0.7 (95% CI = 0.1 to −1.6; Figure 2F) Wood units relative to baseline 60 days after withdrawal of imatinib. This pattern was also observed in the remote measurement of night heart rate. The increase in physical activity observed after drug initiation did not decline after drug withdrawal.

**Figure 3.**
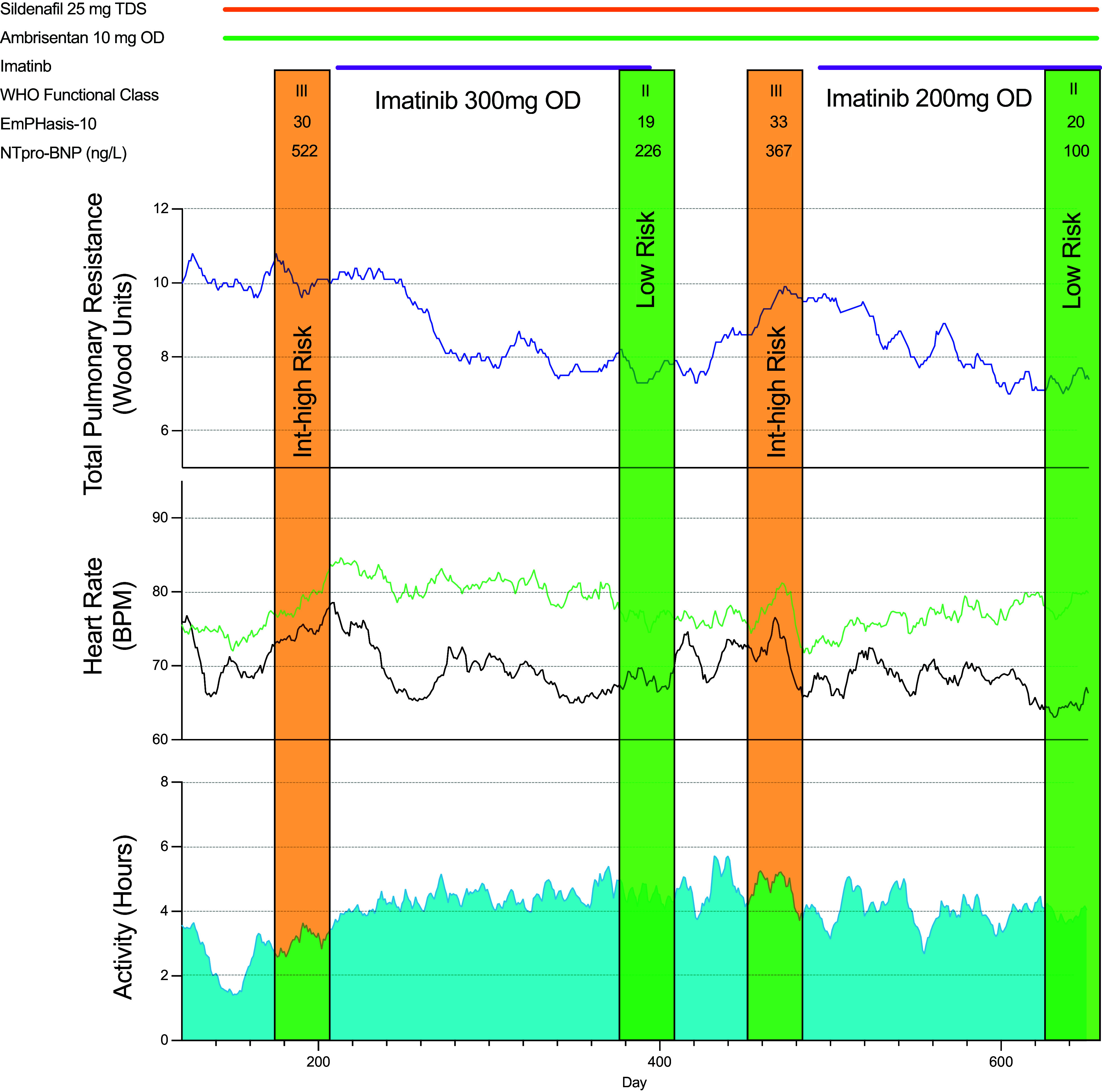
Remote evaluation of physiological effect of imatinib in a patient with pulmonary arterial hypertension. A female in her 40s was established on dual oral therapy with a pulmonary artery pressure monitor and insertable cardiac monitor implanted 8.6 months before enrollment. Initiation of imatinib at 300 mg q.d. was followed by an improvement in World Health Organization (WHO) functional class, EmPHasis-10 questionnaire score, N-terminal pro–B-type natriuretic peptide (NTproBNP), and Comparative, Prospective Registry of Newly Initiated Therapies for Pulmonary Hypertension (COMPERA) 2.0 risk score (change from intermediate–high risk, marked by an orange vertical strip, to low risk, marked by a green strip) and a reduction in total pulmonary resistance and night heart rate. Per-protocol withdrawal resulted in a decline in WHO functional class, EmPHasis-10 score, NTproBNP, and COMPERA 2.0 risk score (second orange vertical strip) and an increase in total pulmonary resistance and night heart rate (black) with maintenance of day heart rate (green). After approval was granted by the Sheffield Teaching Hospitals NHS Foundation Trust Medicines Safety Committee, imatinib was reintroduced off-label at 200 mg q.d., which led to an improvement in WHO functional class, EmPHasis-10, NTproBNP, and COMPERA 2.0 risk score (second green vertical strip) and a reduction in total pulmonary resistance and night heart rate.

One patient was challenged with imatinib, 200 mg q.d., 3 months after completing the study; the positive hemodynamic and functional responses were reproduced (Figure 3).

### Genotype

All patients were genotyped for a C/G polymorphism in *PDGFRB*, which has a pronounced effect on the circulating levels of the encoded protein (20). The distribution of genotypes in the study group was 8GG:7CG:2CC. There was no clear relationship between genotype and reduction in TPR.

### Plasma Imatinib Levels

Exposure to imatinib was dose dependent (Figure 2E). Consistent with other studies (21), there was interindividual variation in plasma levels for a given oral dose, but plasma imatinib levels at steady-state fell within the range predicted using a validated model based on population PK data (15) (*see* Figures E5A and E5B).

## Discussion

We hypothesized that there is an oral dose of imatinib that is tolerated by patients with PAH who are not on anticoagulants that effects a clinically meaningful reduction in PVR. Consistent with the IMPRES (6) report, we found that 400 mg daily is poorly tolerated, but we established that 200 mg is generally acceptable for periods of at least 6 months and results in a clinically significant reduction in TPR and night heart rate and an increase in physical activity. This improvement was observed on the background of combination therapies prescribed according to European guidelines (17).

Investigating the safety and efficacy of a treatment for a rare condition can be challenging and require innovative approaches (22). One such approach is the use of an adaptive study design. The CRM model enabled each sequentially recruited patient to receive the current, best tolerated dose. The successful execution of this study hinged on effective collaboration between clinicians, statisticians, and the trial support team. Data and reports were produced expeditiously after each 4-week outcome period. The data monitoring (and ethics) committee convened within a week or two to review the data and provide recommendations for the next patient, who was already enrolled and ready to commence treatment.

A second innovation was to include patients with implantable PAP sensors and heart rate and activity monitors. These provided longitudinal data, maximized the information obtained from each patient exposure, and enabled direct observation of the time course of the response to imatinib and its withdrawal. A measurable effect on TPR, driven by a fall in mean PAP with no deterioration in cardiac output, was seen within days of starting treatment and increased over 4 weeks before leveling to a sustained reduction. On withdrawal of imatinib, TPR increased gradually toward baseline over 6 weeks and remained below baseline at 8 weeks. Night heart rate followed a similar pattern, whereas physical activity remained increased.

These observations offer some insights into the possible mode of action of imatinib and use of the drug as a treatment. The most commonly available treatments for PAH act primarily through vasorelaxation, providing symptom relief. Imatinib exhibits some vasorelaxant activity in both pulmonary and systemic vascular beds (23, 24). Supporting this mode of action, a reduction in TPR and TSR was seen in patients who provided data through remote monitoring. Nonetheless, the keen interest in imatinib is in its direct vascular antiproliferative activity and its potential to modify the underlying pathophysiology and course of PAH.

Establishing modification of the underlying pathology of PAH in patients is a topical discussion of considerable clinical and regulatory interest (4). Lung biopsies are not considered safe (25). There are no accepted imaging or biochemical surrogate markers of vascular remodeling (4). One suggested criterion for “disease modification” is the persistence of a clinical benefit beyond the short-term pharmacological effects of a drug (4). In the absence of a cure, the duration of sustained benefit after stopping a drug is undefined, but it should continue beyond the period of exposure to the drug. Given a plasma half-life of 15 to 19 hours (26), there would be little active circulating imatinib after 1 week (more than five half-lives). By this criterion, the persistence of a reduced TPR and night heart rate, with a sustained improvement in physical activity, 8 weeks after stopping imatinib would meet this specified criterion for a “disease-modifying effect.” An effect on the trajectory of disease outcomes, another criterion for disease modification (4), remains to be demonstrated, but the lack of a sharp rebound after drug withdrawal does suggest that, unlike with some vasodilators (e.g., prostacyclin), occasional missed doses of the drug would be tolerated (i.e., without a sharp deterioration in the patient). Indeed, given the delayed TPR, heart rate, and physical activity response to imatinib withdrawal, intermittent or pulsed therapy with imatinib after the first 4 weeks of continuous exposure might achieve the same therapeutic benefit while reducing the risk of adverse side effects.

There are ethical considerations in stopping a drug in a patient when that drug appears to be beneficial. The use of implanted devices allows this to be carefully monitored remotely in patients managed according to best practice with a protocol in place to intervene when thresholds are met. Imatinib was reintroduced 12 weeks after withdrawal in one patient because of a gradual deterioration in their condition. The original improvement with imatinib was reproduced. Reproduceable changes on rechallenging highlight another advantage of continuous monitoring of cardiopulmonary hemodynamics in a rare disease; specifically, it enables *n*-of-1 studies and a convincing evaluation of personalized responses (27).

The magnitude of hemodynamic change (−2.8 Wood units at the end of treatment; 95% CI = −1.5 to −4.2 Wood units) from imatinib in our patient cohort compares favorably with recent studies of novel therapies added to background treatment (28–32). When modeled, the predicted reduction in PVR from baseline with sotatercept, an activin ligand trap (28), in a PAH population is around 210 dynes · s/cm^5^ (approximately 2.6 Wood units) (33). Seralutinib, a tyrosine kinase inhibitor, given twice daily by inhalation, reduced PVR by 96 dynes · s/cm^5^ (approximately 1.2 Wood units) over 24 weeks (32). The inhaled route of delivery offers the prospect of reducing systemic exposure and inhaled seralutinib is now in a Phase-3 study. This route of delivery has not proven effective with imatinib (34). This raises the possibility that systemic exposure may be important for imatinib to exert its effect (e.g., through reduction in bone marrow–derived cells) (8). Although 200 mg gives adequate exposure in most patients, given the interindividual variability in pharmacokinetics after oral administration, uptitration from 200 mg daily could be considered in some patients if it is tolerated.

The treatment landscape is changing with the regulatory approval and introduction of sotatercept in many countries. Although its long-term impact remains to be seen, this parenterally administered drug will not suit all patients, and, even if tolerated, many will be left with an elevated PAP (31) and in need of additional treatments. Indeed, although not evaluated in this study, there is no pharmacological contraindication to combining imatinib and sotatercept. The further development of oral imatinib as an add-on therapy is clinically relevant.

### Limitations

First, this study is an open-label design, but the temporal association between change in TPR and imatinib dosing, and the exposure–response relationship, support a drug effect. Second, patients with PAH and implanted devices are a subset of patients that may not represent the PAH population. At the time of device implantation, our patients were required to be in WHO Functional Class 3, with a prior hospitalization within the preceding 12 months. The close support that this patient group receives from healthcare professionals may help with imatinib tolerability. To mitigate against this, some patients without implanted devices were also included, but their numbers were small. Third, the implanted devices measure changes in PAP, and the cardiac output is derived from the waveform, rather than measured. The two measurements provide TPR rather than PVR, and we relied on echocardiography and NTproBNP measurements to exclude a deterioration in cardiac function. Fourth, 3 patients in the remote-monitored group were on calcium channel blockers. Finally, establishing disease modification with current clinical tools is a challenge. Serial hemodynamic measurements after drug withdrawal provide valuable data, but further studies supplemented by timely imaging and patient reported outcomes are needed.

### Conclusions

In summary, imatinib 200 mg q.d. is better tolerated than 400 mg and demonstrated an efficacy signal in patients with PAH, underscoring the importance of reevaluating the dose when repurposing a drug from one indication to another. Integrating remote cardiopulmonary hemodynamic and activity monitoring revealed a slow offset response to withdrawal of treatment, which meets a recently proposed disease-modifying criterion. This property of imatinib lends itself to the possibility of maintaining efficacy while reducing off-target effects through an intermittent or pulsed-dosing regimen.

## Supplemental Materials

10.1164/rccm.202410-1929OC Online data supplement

10.1164/rccm.202410-1929OC Online data supplement
