# Supplementary material for: Positioning Imatinib for Pulmonary Arterial Hypertension: A Dose-Finding Phase 2 Study
Source: Am J Respir Crit Care Med. 2025 Mar 13;211(6):1018–27. doi: 10.1164/rccm.202410-1929OC (PMC12175952; doi:10.1164/rccm.202410-1929OC)
Supplement: Online data supplement [file rccm.202410-1929OCS1.docx]

**Positioning Imatinib for Pulmonary Arterial Hypertension: A Dose Finding Phase 2 Study**

Alexander M K Rothman, Sofia Villar, Jennifer Middleton, Andreas A. Roussakis, Frances Varian, Hamza Zafar, Martin Law, Jane Apperley, Imke H Bartelink, Medhat M Said, Juan A Delgado-SanMartin, David G Kiely, Luke Howard, Mark Toshner, S. John Wort, Martin R Wilkins

**ONLINE DATA SUPPLEMENT**

**Supplementary Figure Legends**

**Supplementary Figure 1:** Implanted remote monitoring devices: Chest x-ray showing placement of remote monitoring devices; CardioMEMS*, a pulmonary artery pressure monitor in a ~10 mm left interlobar pulmonary artery, and LinQ#, a subcutaneous insertable heart rate-physical activity monitor in the left 4th intercostal space

**Supplementary Figure 2:** Overview of study recruitment (Consort diagram) and assessments

**Supplementary Figure 3:** Timeline for recruitment

**Supplemental Figure 4.** Remote monitored haemodynamics**:** Comparison of area under the curve (AUC) for (A) mean pulmonary artery pressure (mPAP), (B) cardiac output and (C) total pulmonary resistance in the 20 days before (pre) and the 20 days after (post) initiation of imatinib (mean±SEM, Wilcoxon matched-pairs signed rank test). WU = Wood units. N=13.

**Supplementary Figure 5: (A) Plasma imatinib concentration profiles at Week 4.** Prediction-corrected visual predictive check of total imatinib (left panel), unbound imatinib (middle panel) and total DM-imatinib (right panel) concentrations using a previously developed population pharmacokinetic model. Black lines represent the observed 5th, 50th, and 95th percentiles of the observed data. The shaded areas represent 95% confidence intervals around the simulated percentiles. **(B)** **Individual Week 4 pharmacokinetic data.** Individual pharmacokinetic simulations for patients with imatinib plasma concentration measured at Week 4. Simulated and observed concentrations for total imatinib (red), unbound imatinib (green) and total DM-imatinib (blue) are shown. Circles represent measured concentrations, while solid lines indicate the simulated profiles using a previously developed population pharmacokinetic model.
