# Supplementary material for: Positioning Imatinib for Pulmonary Arterial Hypertension: A Dose-Finding Phase 2 Study
Source: Am J Respir Crit Care Med. 2025 Mar 13;211(6):1018–27. doi: 10.1164/rccm.202410-1929OC (PMC12175952; doi:10.1164/rccm.202410-1929OC)
Supplement: Online data supplement [file rccm.202410-1929OCS2.pdf]

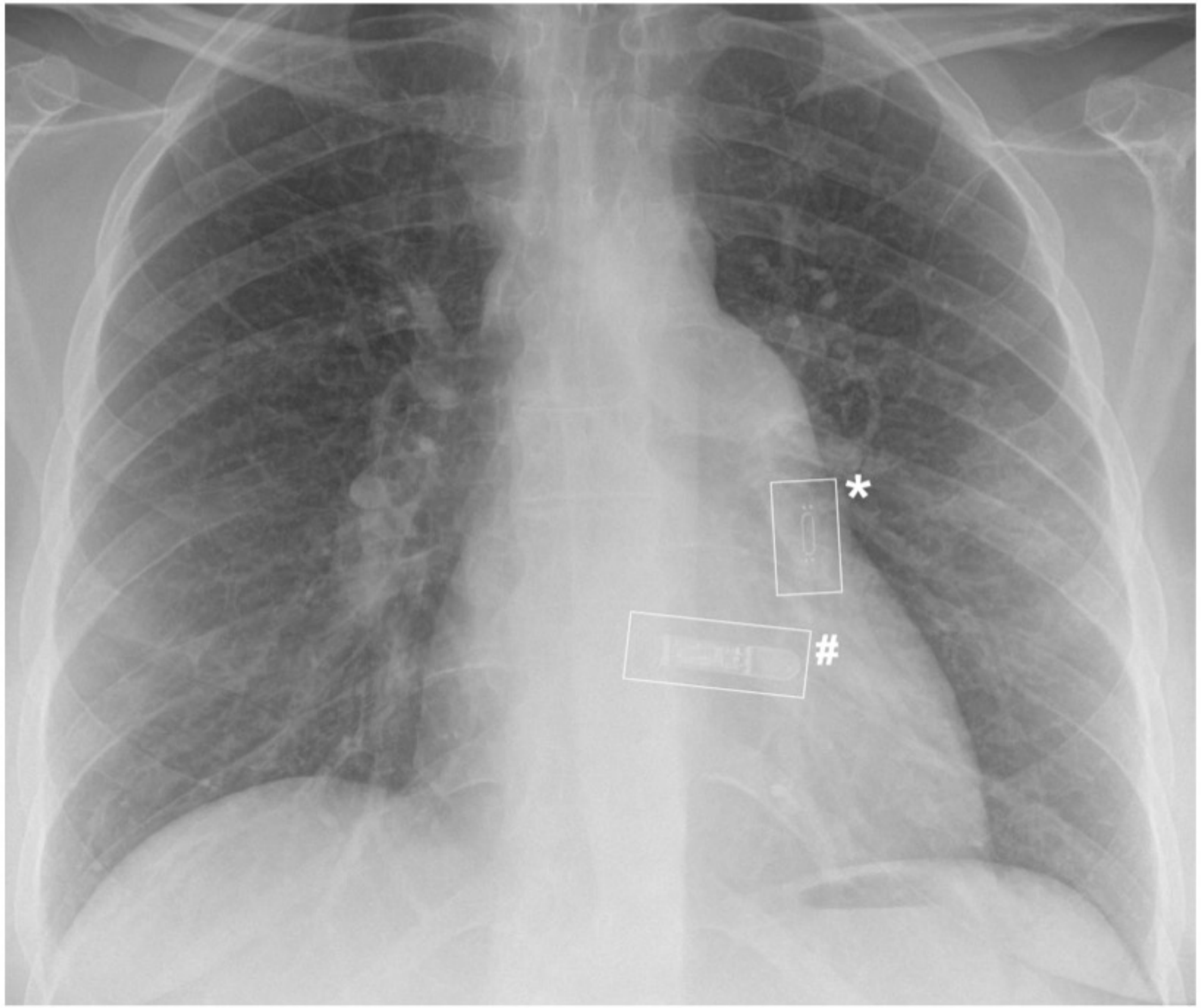


**Supplemental Figure 1: Implanted remote montoring devices:**
Chest x-ray showing placement of CardioMEMS*, a pulmonary artery pressure monitor in a ~10 mm left interlobar pulmonary artery, and LinQ#, a subcutaneous insertable heart rate-physical activity monitor in the left 4th intercostal space

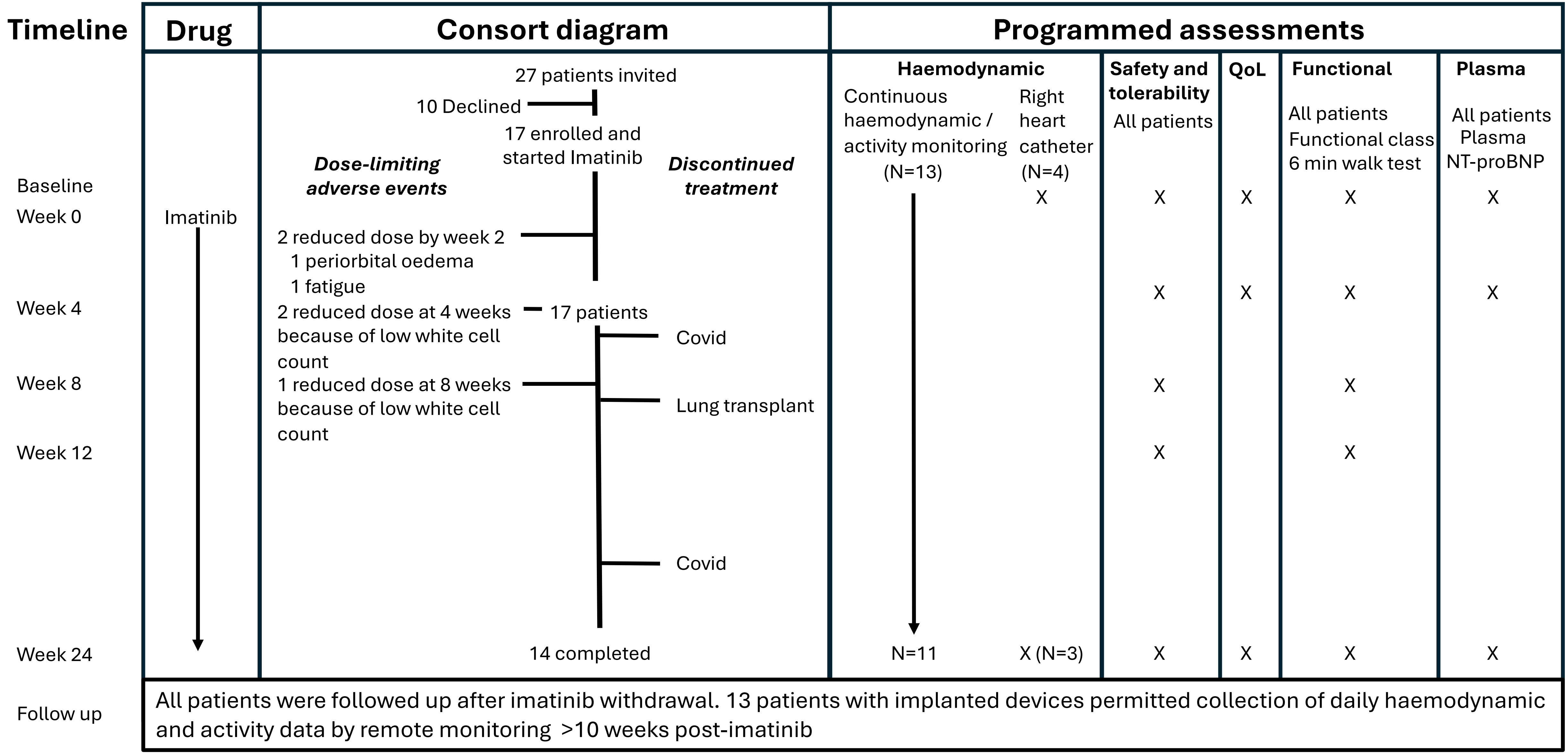


**Supplementary Figure 2: Overview of study recruitment (Consort diagram) and assessments**

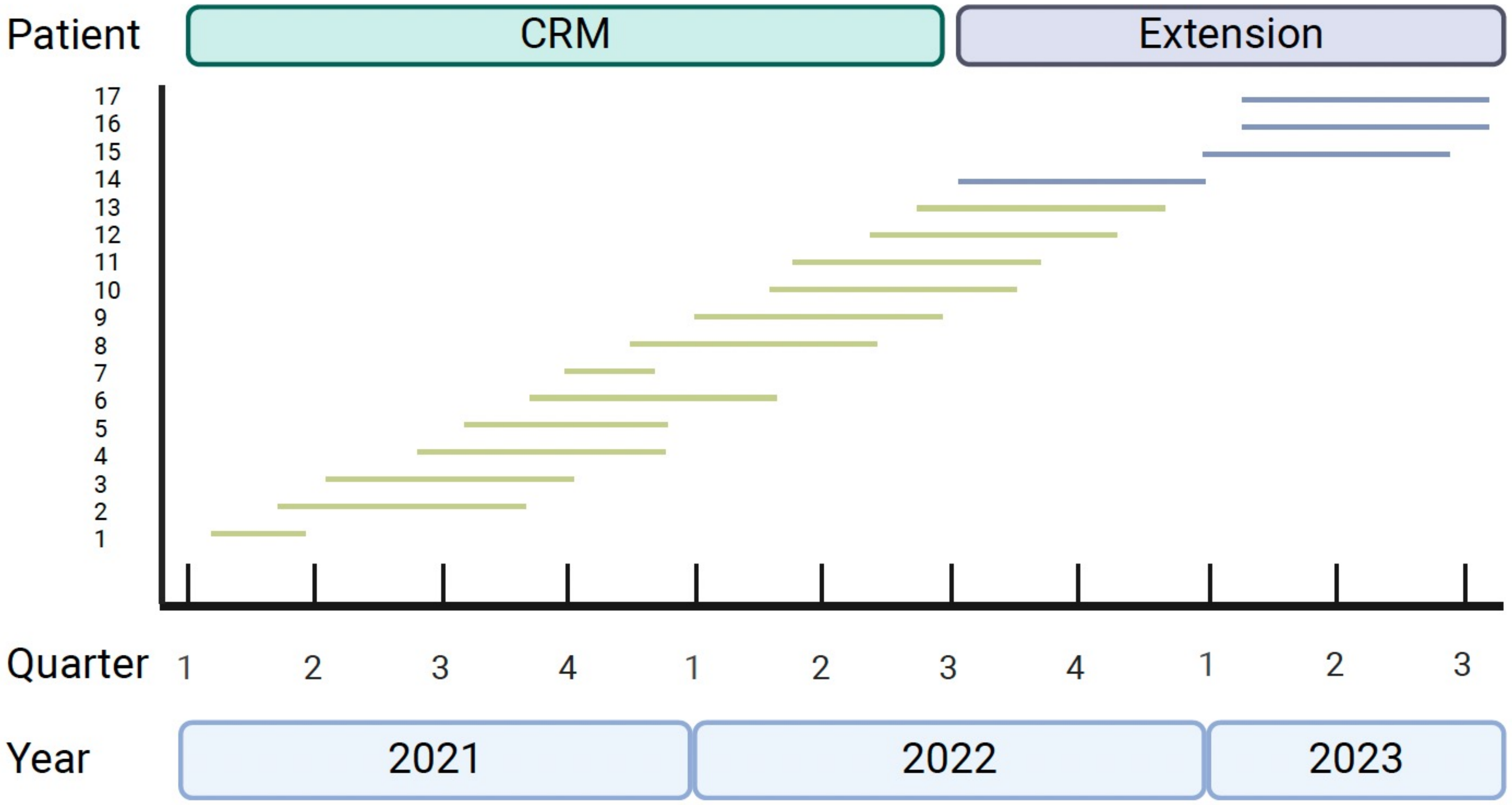


**Supplementary Figure 3: Timeline for recruitment**

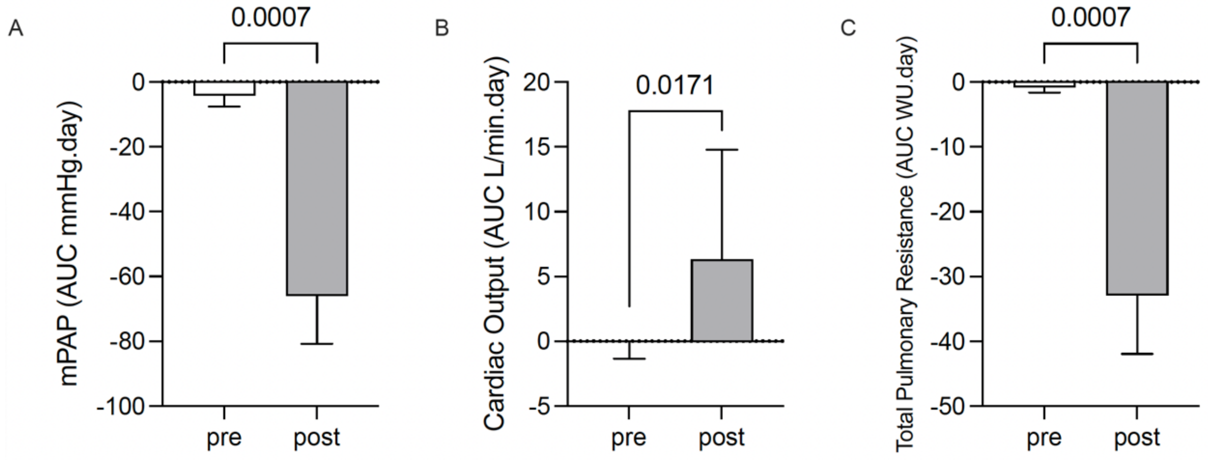


**Supplementary Figure 4: Remote monitored haemodynamics**. Comparison of area under the curve (AUC) for (A) mean pulmonary artery pressure (mPAP), (B) cardiac output and (C) total pulmonary resistance in the 20 days before (pre) and the 20 days after (post) initiation of imatinib (mean +/- SEM, Wilcoxon matched-pairs signed rank test). WU = Wood Units. N=13.

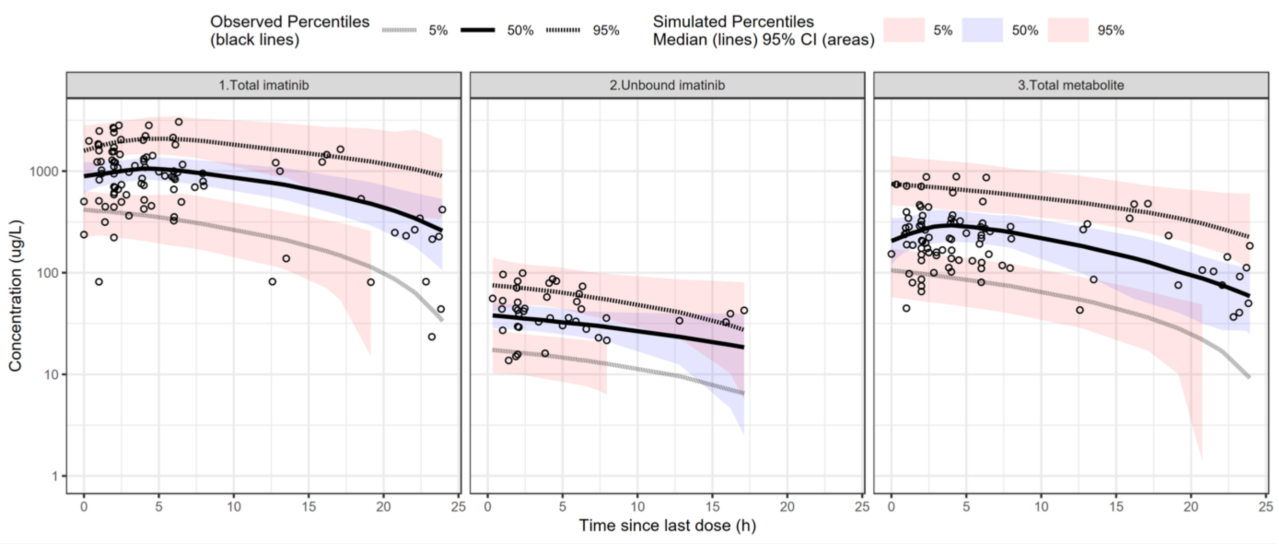


**Supplementary Figure 5A: Plasma imatinib concentration profiles at Week 4.** Prediction-corrected visual predictive check of total imatinib (left panel), unbound imatinib (middle panel) and total DM-imatinib (right panel) concentrations using a previously developed population pharmacokinetic model. Black lines represent the observed 5th, 50th, and 95th percentiles of the observed data. The shaded areas represent 95% confidence intervals around the simulated percentiles.

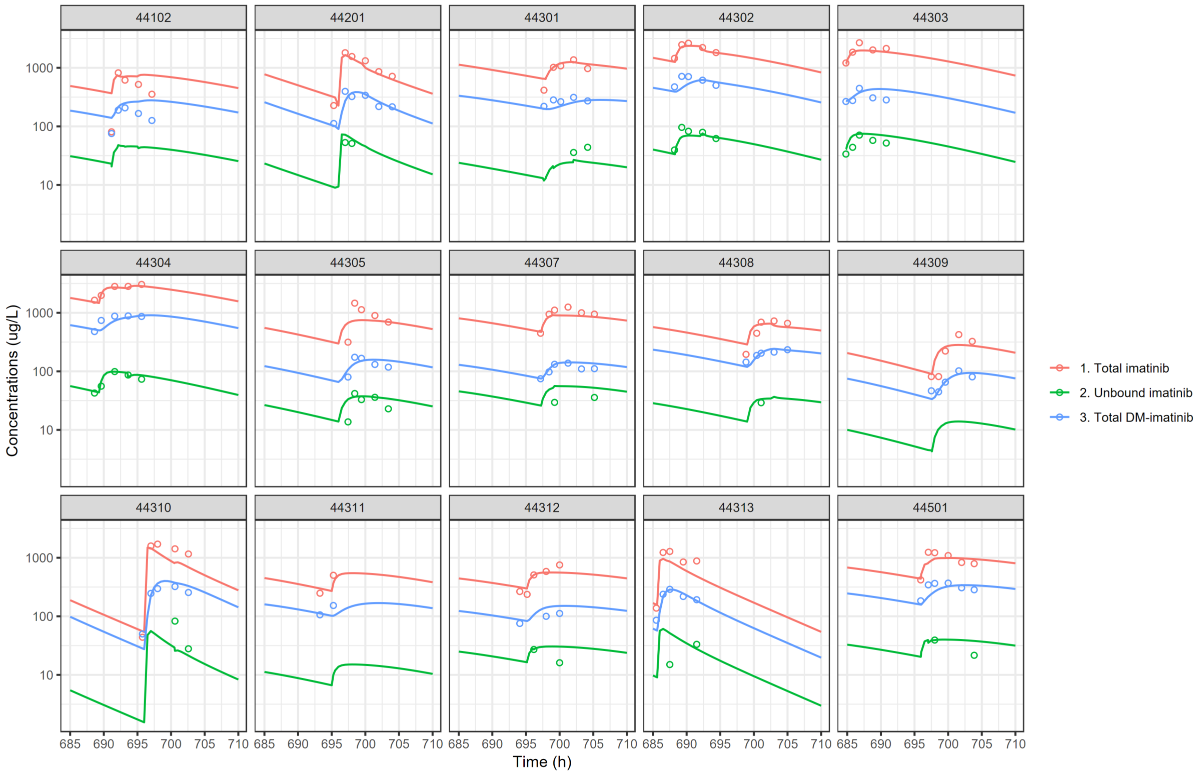


**Supplementary Figure 5B: Individual Week 4 pharmacokinetic data.** Individual pharmacokinetic simulations for patients with imatinib plasma concentration measured at Week 4. Simulated and observed concentrations for total imatinib (red), unbound imatinib (green) and total DM-imatinib (blue) are shown. Circles represent measured concentrations, while solid lines indicate the simulated profiles using a previously developed population pharmacokinetic model.

## Supplementary Tables

| Measurement | Baseline | | Follow up | | Diff | Pval |
|---|---|---|---|---|---|---|
| TAPSE | 22.67 | 2.92 | 24.56 | 3.54 | 1.89 | 0.102 |
| esPAP | 55.89 | 17.3 | 51.33 | 18.2 | -4.56 | 0.129 |
| RA area | 21.08 | 3.65 | 19.75 | 4.2 | -1.33 | 0.156 |
| TAPSE/sPAP | 0.45 | 0.18 | 0.5461 | 0.25 | 0.096 | 0.547 |

**Supplementary Table 1**: **Echocardiography measurements in patients with implanted devices.** Change in mean (SD) tricuspid annular plane systolic excursion (TAPSE), end systolic pulmonary artery pressure (esPAP), right arial (RA) area, and ratio of TAPSE to systolic pulmonary artery pressure (TAPSE/PAP). N=12

# Research Protocol

## Positioning Imatinib for Pulmonary Arterial Hypertension
## - PIPAH Study-

| | |
|---|---|
| **Protocol Version:** | 2.0, 06-OCT-2020 |
| **Superseding:** | 1.1, 16-JUL-2020 |
| **NIHR Ref.:** | NIHR128465 |
| **R&D Ref.:** | 20HH5896 |
| **IRAS registration:** | 274093 |
| **REC Ref.:** | 20/SC/0240 |
| **MHRA Ref.:** | CTA 19174/0419/001-0001 |
| **EudraCT Register:** | 2020-001157-48 |
| **ClinicalTrials.gov:** | NCT04416750 |

| | |
|---|---|
| **Sponsor:** | Imperial College London, UK |
| **Funding:** | Efficacy and Mechanism Evaluation (EME) Programme, an MRC and NIHR partnership |
| **Chief Investigator:** | Professor Martin R. Wilkins |

| | | |
|---|---|---|
| **Developed by:** | Professor MR Wilkins, Dr AA Roussakis | |
| **Authorised by:** | *Martin R. Wilkins, Chief Investigator*<br>*Andreas A. Roussakis, Clinical Project Manager* | **Name, Role** |
| | *electronically signed* | **Signature** |
| | *06-OCT-2020* | **Date** |

This protocol describes the above study and provides information about procedures for entering participants. The protocol should not be used as a guide for the treatment of other participants; every care was taken in its drafting, but corrections or amendments may be necessary. These will be circulated to the Investigators of the study, but centres entering participants for the first time are advised to contact the trials centre to confirm they have the most recent version. Problems relating to this trial should be referred, in the first instance, to the study coordination centre. This trial will adhere to the principles outlined in the Medicines for Human Use (Clinical Trials) Regulations 2004 (SI 2004/1031), amended regulations (SI 2006/1928) and the International Conference on Harmonisation Good Clinical Practice (ICH GCP) guidelines. It will be conducted in compliance with the protocol, the Data Protection Act and other regulatory requirements, as appropriate.

**PROTOCOL AMENDMENTS**
**Positioning Imatinib for Pulmonary Arterial Hypertension**
**-PIPAH Study-**

The original protocol for study has been amended and re-issued as follows. See the summary of changes in Appendix 3.

| **Substantial Amendment** | **Date** |
|---|---|
| AM01 | 06-OCT-2020 |

## GLOSSARY OF ABBREVIATIONS

| | |
|---|---|
| 6MWD | 6-Minute Walk Distance |
| 6MWT | 6-Minute Walk Test |
| AE | Adverse Event |
| AR | Adverse Reaction |
| β-hCG | beta human Chorionic Gonadotropin |
| BP | Blood Pressure |
| CRF | Case Report Form |
| eCRF | electronic Case Report Form |
| FSH | Follicle Stimulating Hormone |
| GCP | Good Clinical Practice |
| ICH | International Conference on Harmonisation |
| IMP | Investigation Medicinal Product |
| MHRA | Medicines and Healthcare Products Regulatory Agency |
| MRI | Magnetic Resonance Imaging |
| PAH | Pulmonary Arterial Hypertension |
| PCR | Polymerase Chain Reaction |
| PDGF | Platelet Derived Growth Factor |
| PDGFR | Platelet Derived Growth Factor Receptor |
| PDGFRB | Platelet Derived Growth Factor Receptor Beta protein coding gene |
| pQTL | protein Quantitative Trait Locus |
| PVR | Pulmonary Vascular Resistance |
| QoL | Quality of Life |
| REC | Research Ethics Committee |
| RHC | Right Heart Catheterisation |
| SAE | Serious Adverse Event |
| SAR | Serious Adverse Reaction |
| SNP | Single Nucleotide Polymorphism |
| SUSAR | Suspected Unexpected Serious Adverse Reaction |
| TKI | Tyrosine Kinase Inhibitor |
| TTL | Target Toxicity Level |
| WHO | World Health Organisation |

**Keywords:** Pulmonary Arterial Hypertension; PAH; Imatinib; TKI

## STUDY SUMMARY

<table>
<tr><td>TITLE:</td><td colspan="3">Positioning Imatinib for patients with Pulmonary Arterial Hypertension (PIPAH Study)</td></tr>
<tr><td>PHASE:</td><td colspan="3">II</td></tr>
<tr><td>INVESTIGATIONAL MEDICINAL PRODUCT:</td><td colspan="3">Imatinib mesilate</td></tr>
<tr><td>DESIGN:</td><td colspan="3">Open-label, multi-centre comprising two parts - see Appendix 1</td></tr>
<tr><td></td><td colspan="3">Part 1 uses the Bayesian continual reassessment method to define the highest tolerated dose.<br>Part 2 takes this dose forward into an endpoint efficacy study using a Simon’s optimal two-stage design.</td></tr>
<tr><td>OBJECTIVE(S):</td><td colspan="3">Part 1: To identify the highest tolerated dose of Imatinib (between 100mg and 400mg once daily for 4 weeks) in patients with Pulmonary Arterial Hypertension (PAH).<br><br>Part 2: To assess the efficacy of Imatinib administered at the highest tolerated dose (once daily for 24 weeks) on pulmonary vascular resistance (PVR) in PAH patients according to genes that influence the actions of PDGF (platelet derived growth factor).</td></tr>
<tr><td>INTERVENTION:</td><td colspan="3">Imatinib - oral administration once daily for up to 24 weeks</td></tr>
<tr><td>PRIMARY OUTCOME MEASURES:</td><td colspan="3">Part 1: Discontinuation of the drug for more than 5 consecutive days due to Grade 2 or above Adverse Events defined by the National Cancer Institute (NCI) Common Terminology Criteria for Adverse Events (version 5.0, 2017), adapted for this study - see Appendix 2<br><br>Part 2: The primary efficacy endpoint is a binary variable. For patients with a baseline PVR >1000 dynes·s·cm$^{-5}$, success is defined by an absolute reduction in PVR of ≥300 dynes·s·cm$^{-5}$ at 24 weeks. For patients with a baseline PVR ≤1000 dynes·s·cm$^{-5}$, success is a 30% reduction in PVR at 24 weeks.</td></tr>
<tr><td>POPULATION:</td><td colspan="3">Up to 43 individuals with PAH. Recruitment is competitive among sites.</td></tr>
<tr><td rowspan="9">STUDY CENTRES:</td><td>Site ID</td><td>Site Name</td><td>Academic Affiliation</td></tr>
<tr><td>4410</td><td>Hammersmith Hospital</td><td>Imperial College London</td></tr>
<tr><td>4420</td><td>Royal Papworth Hospital</td><td>University of Cambridge</td></tr>
<tr><td>4430</td><td>Royal Hallamshire Hospital</td><td>University of Sheffield</td></tr>
<tr><td>4440</td><td>Royal Free Hospital</td><td>University College London</td></tr>
<tr><td>4450</td><td>Royal Brompton Hospital</td><td>Imperial College London</td></tr>
<tr><td>4460</td><td>Golden Jubilee National Hospital</td><td>University of Glasgow</td></tr>
<tr><td>4470</td><td>Newcastle Freeman Hospital</td><td>Newcastle University</td></tr>
<tr><td>4480</td><td>Royal United Hospital</td><td>University of Bath</td></tr>
<tr><td>STUDY DURATION:</td><td colspan="3">Up to 48 months<br>First Patient First Visit: Q4, 2020<br>Last Patient Last Visit: Q2, 2024</td></tr>
</table>

| | |
|---|---|
| **ELIGIBILITY** | **Inclusion criteria:**<br>**1.** Subjects aged between 18-75 years old<br>**2.** PAH which is idiopathic, heritable or associated with anorexigens<br>**3.** Subjects willing to be genotyped for genes that influence PDGF activity<br>**4.** Resting mean pulmonary artery pressure >25 mmHg, pulmonary capillary wedge pressure ≤15 mmHg, PVR >5 wood units, and normal or reduced cardiac output, as measured by right heart catheterisation (RHC) at entry<br>**5.** Six-minute walking distance >50m at entry<br>**6.** Stable on an unchanged PAH therapeutic regime comprising at least 2 therapies licensed for PAH (any combination of endothelin receptor antagonist, phosphodiesterase inhibitor or prostacyclin analogue) for at least 1 month prior to screening<br>**7.** Able to provide written informed consent prior to any study mandated procedures<br>**8.** Contraception: Fertile females (women of childbearing potential) are eligible to participate after a negative highly sensitive pregnancy test, if they are taking a highly effective method of contraception during treatment and until the end of relevant systemic exposure. Fertile males who make use of condom and contraception methods during treatment and until the end of relevant systemic exposure in women of childbearing potential **-see details in section 4.3.1-**<br><br>**Exclusion criteria:**<br>**1.** Unable to provide informed consent and/or are non-fluent speakers of the English language<br>**2.** Hypersensitivity to Imatinib or to any of the excipients<br>**3.** Clinically-significant renal disease (confirmed by creatinine clearance <30 ml/min per $1.73m^2$)<br>**4.** Clinically-significant liver disease (confirmed by serum transaminases >3 times than upper normal limit)<br>**5.** Patients receiving oral and/or parenteral anticoagulants*<br>**6.** Anaemia confirmed by haemoglobin concentration <10 g/dl<br>**7.** History of thrombocytopenia<br>**8.** Individuals known to have haemoglobinopathy sickle cell disease, thalassaemia<br>**9.** Hospital admission related to PAH or change in PAH therapy within 3 months prior to screening<br>**10.** History of left-sided heart disease and/or clinically significant cardiac disease, including but not limited to any of the following:<br>a. Aortic or mitral valve disease (stenosis or regurgitation) defined as greater than mild aortic insufficiency, mild aortic stenosis, mild mitral stenosis, moderate mitral regurgitation<br>b. Mechanical or bioprosthetic cardiac valve<br>c. Pericardial constriction, effusion with tamponade physiology, |

| | |
|---|---|
| | or abnormal left atrial size.<br>d. Restrictive or congestive cardiomyopathy<br>e. Left ventricular ejection fraction ≤50% (measured in echocardiogram at screening)<br>f. Symptomatic coronary disease<br>g. Significant (2+ for regurgitation) valvular disease other than tricuspid or pulmonary regurgitation<br>h. Acutely decompensated left heart failure within 1 month of screening<br>i. History of untreated obstructive sleep apnoea<br>**11.** Evidence of significant lung disease on high-resolution CT (if available) or recent (performed within 12 months) lung function, where FEV1 < 50% predicted and FVC < 70% predicted, and DLCO (or TLCO) < 50% predicted if any CT abnormalities; judged by the Site Physician<br>**12.** Patients with a history of uncontrolled systemic hypertension<br>**13.** Acute infection (including eye, dental, and skin infections)<br>**14.** Chronic inflammatory disease including HIV, and Hepatitis B<br>**15.** Women of childbearing potential who are pregnant or breastfeeding (if applicable)<br>**16.** Previous intracerebral haemorrhage<br>**17.** Patients who have received an Investigational Medicinal Product (IMP) within 5 half-lives of the last dose of the IMP or 1 month (whichever is greater) before the baseline visit<br>**this does not apply to single antiplatelet therapy* |
| **TREATMENT:** | There are two parts to the study. Part 1 will recruit 6 to 13 patients. The objective is to identify the highest tolerated dose. Part 2 will follow on. It will recruit a separate group of patients, up to 34, to evaluate the efficacy of the highest tolerated dose.<br><br>Patients will be invited to participate by one of the 8 NHS partner sites specialised in the management of PAH. Suitable patients will be treated with Imatinib once daily for up to 24 weeks. Patients will be asked to keep diaries for as long as they will be taking the study drug to ensure compliance.<br><br>Part 1: Some patients will have an insertable FDA/CE cardiac rhythm monitor and pulmonary artery pressure monitor that captures cardiopulmonary haemodynamics and daily activity. These patients will be provided with a remote monitoring system to capture daily blood pressure, oxygen saturations and body weight. This permits remote monitoring with the option to attend the hospital, as required. The patients without a monitor will attend their specialist hospital site for a baseline assessment (inclusive of a right heart catheterisation, RHC) , and then again at the end of Week 4 to assess tolerability, with telephone assessments at the end of Weeks 1, 2 and 3.<br><br>The first patient will receive Imatinib 100mg (x1) once daily. Subsequent patients will enter the trial at no less than 4 week intervals and receive |

| | |
|---|---|
| | a dose of Imatinib (up to 400mg) based on the experience of the previous patient(s) at 4 weeks, using a statistical model to identify the most likely highest tolerated dose. By definition, the highest tolerated dose is reached in this trial when there is a 20% probability that a patient will not be able to continue the drug for 5 consecutive days.<br><br>All patients who are tolerating Imatinib will be asked to continue on their assigned dose to the end of week 24 and their clinical response will be measured by the change in PVR (measured using the implanted monitor or, where a patient does not have a monitor by RHC).<br><br>Part 2: All patients will receive the highest tolerated dose (defined in Part 1) for 24 weeks. Patients will be recruited as suitable (i.e. there is no staggering) and monitored for safety at the end of Weeks 4, 8, 12 and 24, with telephone assessments at the end of Weeks 16, 20 and 28. Clinical response will be measured by change in PVR from baseline at Week 24. The primary efficacy endpoint is a binary variable: for patients with a baseline PVR >1000 dynes·s·cm$^{-5}$, success is defined by an absolute reduction in PVR of ≥300 dynes·s·cm$^{-5}$ at 24 weeks; for patients with a baseline PVR ≤1000 dynes·s·cm$^{-5}$, success is a 30% reduction in PVR at 24 weeks.<br><br>An interim analysis is planned after 13 patients have completed the 24-week treatment on the highest tolerated dose; some patients from Part 1 will be evaluable if they have completed 24 weeks on the highest tolerated dose. If there are 1 or no responses in these patients, the study will be stopped. Otherwise, additional patients will be recruited for a total of 34 evaluable patients (target for Part 2). The null hypothesis will be rejected if 7 or more responses are observed in the 34 patient cohort.<br><br>Blood levels of Imatinib will be measured in each patient at baseline and at the end of Weeks 4 and 24. A trough level (noting the time for last dose) will be measured at Week 4 and Week 24. |

# 1. BACKGROUND AND RATIONALE

## 1.1 Pulmonary arterial hypertension (PAH)

PAH is an uncommon condition characterised by pre-capillary resistance to pulmonary blood flow in the absence of airway or parenchymal lung diseases, left heart failure or chronic thromboembolism (1,2). The resultant elevation in pulmonary artery pressure places an increased pressure load on the right ventricle, leading to right heart failure and premature death.

In around 50% of patients there is no identifiable underlying cause, and patients are classified as idiopathic PAH or, where there is a family history, heritable PAH. Histological examination of post-mortem or transplantation PAH lung tissue shows marked pulmonary arterial remodelling with vascular cell proliferation narrowing the vascular lumen (3).

The estimated prevalence of PAH is 15 per million (2,4). It affects people in middle age and is an unmet clinical need. Five-year mortality for idiopathic/heritable PAH managed by experienced centres in the UK is around 58% (5). The current licensed treatments [prostanoids, endothelin receptor antagonists, phosphodiesterase type 5 inhibitors, and soluble guanylate cyclase stimulator] focus on pharmacologically manipulating 3 signaling pathways better known for regulating vascular tone

(1,2,4). These treatments have little impact on the underlying vascular remodelling and do not arrest or reverse the course of the condition.

The past few years have seen attempts to target pulmonary vascular remodelling directly with antiproliferative or anti-inflammatory drugs (6). To date, the most promising data have come from studies with Imatinib (7-10); however, serious concerns over its safety and tolerability have limited its development as a treatment for PAH (11).

**1.2 Imatinib**

Imatinib is an orally active tyrosine kinase inhibitor (TKI) that inhibits platelet-derived growth factor receptor (PDGFR) alpha and beta, BCR-ABL, DDR and c-KIT (12). PDGF is a potent vascular smooth muscle cell mitogen (13). Studies of lung tissue from PAH patients and animal models indicate that PDGF is important in vascular smooth muscle proliferation and hyperplasia (6). Histological studies of human PAH lung show infiltration of pulmonary arterial lesions with c-kit(+) cells, which may also participate in vascular remodelling (14, 15). Imatinib consistently reverses pulmonary hypertension in experimental models (7).

These data are supported by two placebo-controlled randomised clinical trials; a Phase II involving 59 patients (9) and a Phase III including 202 patients (10) have provided evidence of clinical efficacy in PAH. The Phase III IMPRES study (10) reported a significant improvement in functional capacity (the mean placebo-corrected treatment effect on six-minute walk distance (6MWD) was 32m; 95% confidence interval, 12-52; P=0.002) and a reduction in pulmonary vascular resistance (PVR) of 379 dynes·s·cm$^{-5}$ (95% confidence interval, −502 to −255; P<0.001, between-group difference) with Imatinib at 24 weeks compared to placebo in patients already receiving their licensed vasodilatory treatments (10). Unfortunately, Serious Adverse Events (SAEs) and discontinuations were more common in the Imatinib group (10).

Of particular concern, eight patients enrolled in IMPRES (10) developed a subdural haematoma while taking Imatinib, leading to death in two. These patients were taking an anticoagulant, warfarin, at the same time as Imatinib. An interaction between these two drugs is strongly suspected although the mechanism of this interaction is not known.

Despite these concerns, in the absence of alternative effective treatments, there remains considerable interest in the use of Imatinib for PAH. It is raised at all international meetings discussing PAH and drug development. As an indicator of clinical need, Imatinib continues to be used worldwide on compassionate grounds as a treatment of last resort and up to 100 patients may be taking it across Europe. Many specialist centres have case reports where patients have been ‘rescued’ by the drug.

**Recent UK case study - April 2019**

A 46-year-old female patient with idiopathic PAH in WHO functional class IV (syncope) with a PVR of 1690 dynes·s·cm$^{-5}$ was being treated with two licensed therapies (Tadalafil and Macitentan). She was intolerant of prostanoids and had a contraindication to lung transplantation.

She received oral Imatinib as a last resort. She tolerated a dose of 100mg daily for the past 9 months and on recent review her recent PVR had fallen to 1120 dynes·s·cm$^{-5}$. Her 6MWD had increased from 144 to 405m. She had suffered no further episodes of syncope and the repeated hospitalisations with heart failure have also stopped.

There is a clear need for a study to revisit and evaluate the clinical potential of Imatinib in PAH in a stratified way. To address safety concerns, this study should (a) revisit the dose, and (b) seek to identify patients most likely to respond and reduce exposure to those least likely to benefit.

(a) The lowest efficacious dose of Imatinib in PAH has not been established. The target dose of Imatinib (400mg) used in clinical studies to date (9,10) was adopted from its use in chronic myeloid leukaemia. Clinical experience in patients receiving Imatinib on compassionate grounds indicate that lower does, even as low as 100mg daily, may be effective. Of note (*vide infra*), patients with clonal eosinophilia driven by overexpression of Platelet Derived Growth Factor Receptor Beta (PDGFRB) respond rapidly to low doses of Imatinib (100mg to 200mg daily) (16,17). The hypothesis that patients with PAH may respond to low doses of Imatinib needs formal investigation.

(b) Clinical studies and physician experience suggest that some patients benefit substantially from addition of the drug to existing therapy (8,18-20). Some patients demonstrate large (>30%) reductions in PVR - see Figure 4. A responder analysis of patients in the Phase III study shows that some patients gained a >60m increase in 6MWD (10). We have insight into a genotype and panel of biomarkers that may identify patients most likely to benefit from Imatinib and inform early stop-go decisions in individual patients. In addition to a lower dose, targeting Imatinib to those patients most likely to benefit and stopping treatment early if they are not responding would improve the benefit-harm profile of Imatinib for PAH.

### 1.3 Targeting treatment with Imatinib - work to date

Patients with a diagnosis of PAH attend one of the UK centres that specialise in the management of PAH. Since 2013, we have recruited and followed a group of patients with idiopathic or heritable PAH as part of a cohort study (http://www.ipahcohort.com/). We currently have over 650 well-phenotyped patients with whole genome sequence data (21). We have plasma proteomic data (1,124 proteins using the aptamer-based assay from SomaScan) on 354 patients (22).

#### 1.3.1 Expression of PDGFRB is influenced by common gene variant

A review of the literature shows three separate population-based studies that have identified a cis-acting protein quantitative trait locus (pQTL) at PDGFRB for circulating levels of PDGFRB reaching genome-wide significance (P-value $<3\times10^{-113}$). We have reproduced this in our PAH cohort (rs2304058 variant, P-value $<1.88\times10^{-92}$) - see Figure 1.

**Figure 1 -** pQTL for PDGFRB

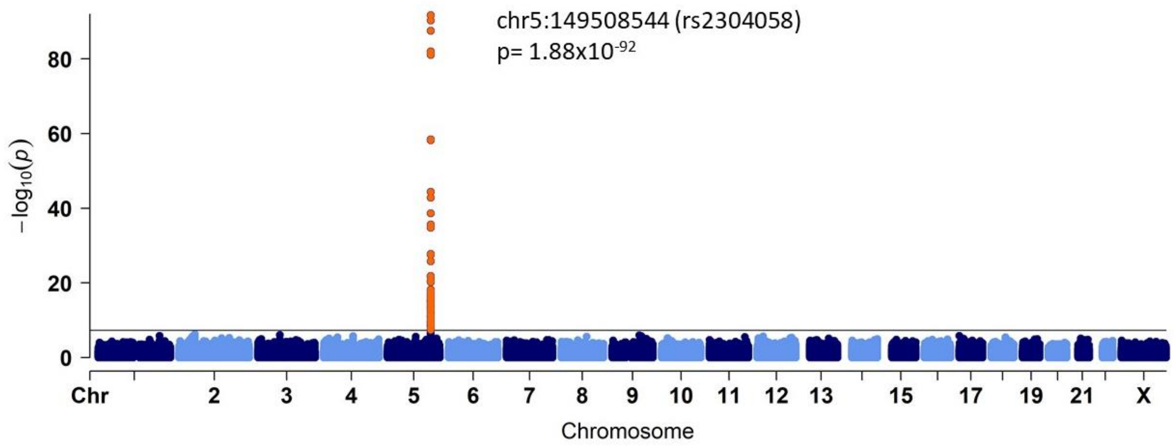


Further analysis shows a 1.6-fold increase in circulating PDGFRB levels per risk allele - see Figure 2. The difference between patients homozygous for the reference and risk alleles is approximately 3.5 standard deviations. If PDGFRB is an important target for Imatinib in PAH, PDGFRB genotype may influence therapeutic response, or at least the dose required to elicit a response and the risk of dose-limiting side effects.

**Figure 2 -** Relationship of PDGFRB pQTL to plasma PDGFRB levels in PAH patients

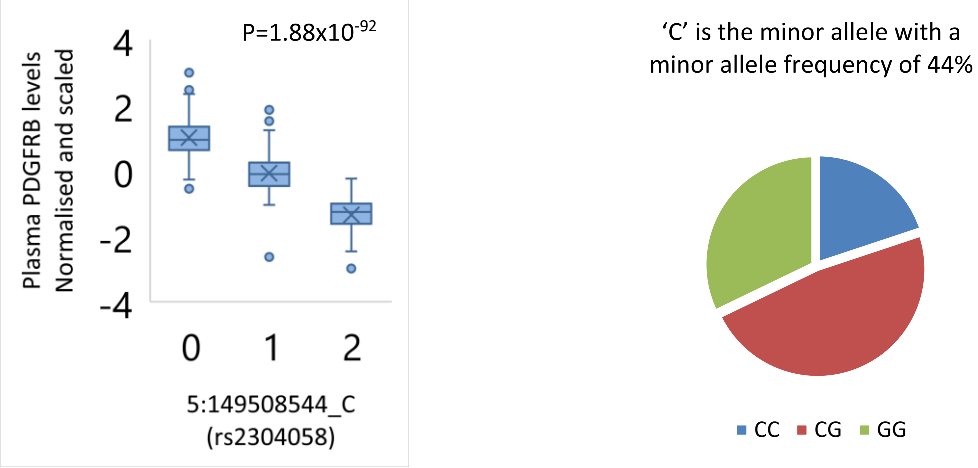


### 1.3.2 Circulating proteins that track and/or predict response

Working with collaborators at Harvard (led by Dr Brad Maron), we accessed a protein-protein interactome network (comprising around 350,000 protein-protein interactions connecting approximately 17,000 proteins) and mapped the proximity of the top 20 proteins associated with Imatinib -see Figure 3a- to proteins associated with PAH. Within this list were PDGFRB, ABL1 and ABL2, c-Kit and EGFR (ErbB1). We analysed paired plasma proteome data (measured in the SomaScan assay) from 14 patients treated with Imatinib for at least 6 months in IMPRES (10) and identified 166 proteins that changed in response to treatment (P<0.05 paired t-test). From a list of top 20 proteins from the proximity analysis, the change in circulating levels of c-Kit was statistically significant after false discovery rate correction and the change in levels of 2 other proteins, RET proto-oncogene, and mitogen-activated protein kinase 9, reached nominal significance - see Figure 3.

**Figure 3 -** (a) Protein network associated with Imatinib showing PDGFRB circled; (b) Change in plasma levels after Imatinib

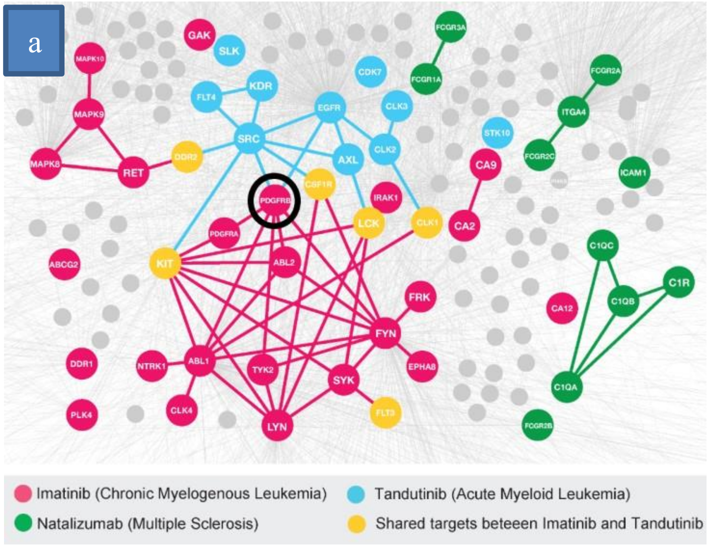


b

| TubeUniqueID | Difference | paired ttest sig |
|---|---|---|
| SCF sR | -0.17 | 4E-05 |
| RET | -0.03 | 0.044 |
| JNK2 | -0.03 | 0.028 |
| LYNB | -0.11 | 0.146 |
| PDGF Rb | -0.05 | 0.268 |
| FYN | 0.30 | 0.218 |
| ERBB1 | 0.04 | 0.172 |
| MK08 | -0.17 | 0.082 |
| ABL1 | 0.06 | 0.223 |
| LYN | -0.10 | 0.377 |
| ABL2 | -0.05 | 0.386 |
| FGR | -0.06 | 0.131 |
| LCK | -0.01 | 0.677 |
| TrkA | -0.05 | 0.628 |
| TYK2 | 0.00 | 0.372 |

Five of the 20 proteins from the proximity analysis (RAF1, Syk, IRAK1, ABCB1 and CLK1) are not on the 1,124 SomaScan platform. Interestingly, the plasma proteome analysis detected changes in the circulating levels of 3 proteins (fibroblast activation protein-alpha/Seprase, and Stem Cell Growth Factor (alpha and beta) after false discovery rate correction that did not emerge from the proximity analysis; a working hypothesis is that the changes in levels of these proteins may indicate exposure to Imatinib but independent of any impact on underlying pathology of PAH.

**Figure 4 -** Relationship of baseline ErbB3 to change in PVR post-Imatinib

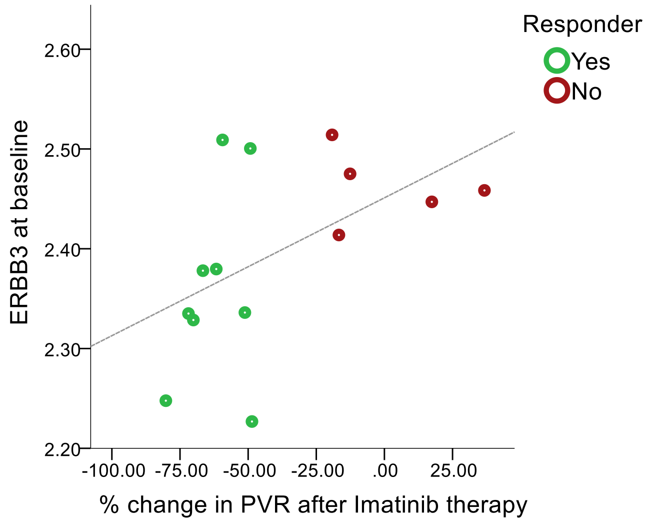


We also investigated the relationship between baseline levels of proteins captured in the proteome analysis of 14 patients treated with Imatinib and response to the drug, assessed by change in PVR, and identified an association with plasma ErbB3 levels; low ErbB3 levels at the start of treatment were associated with a greater change in PVR - see Figure 4. This needs further validation. From this analysis, we suggest that the PDGFRB pQTL and/or a panel of proteins including c-Kit, RET, JNK2 and ERBB3 will inform the response of PAH patients to Imatinib.

## 2. STUDY OBJECTIVES

Part 1: To identify the highest tolerated dose of Imatinib (in the range of 100mg and 400mg once daily for 4 weeks) in patients with PAH.

Part 2: To assess the efficacy of Imatinib administered at the highest tolerated dose (once daily for 24 weeks) on PVR in PAH patients according to genes that regulate PDGF activity.

## 3. STUDY OUTCOME MEASURES

### 3.1 Primary outcome measures:

- Part 1: Discontinuation of the drug for more than 5 consecutive days due to Grade 2 or above Adverse Events defined by the National Cancer Institute (NCI) Common Terminology Criteria for Adverse Events (version .5.0, 2017), adapted for this study - see Appendix 2.

- Part 2: The primary efficacy endpoint is a binary variable. For patients with a baseline PVR >1000 dynes·s·cm$^{-5}$, success is defined by an absolute reduction in PVR of ≥300 dynes·s·cm$^{-5}$ at 24 weeks. For patients with a baseline PVR ≤1000 dynes·s·cm$^{-5}$, success is a 30% reduction in PVR at 24 weeks.

### 3.2 Secondary outcome measures:

- Change in PVR from baseline according to genes that regulate PDGF activity.
- Change in 6MWD at 24 weeks.
- Change in right ventricular ejection fraction (RVEF) values, measured in echocardiogram (at screening assessment and at 24 weeks).
- Change in plasma brain natriuretic peptide (proNT-BNP) levels from baseline at 24 weeks.
- Change in Quality of Life (QoL) scores (23) from baseline at 24 weeks.

### 3.3 Exploratory outcome measures:

- Change in plasma proteome from baseline at 24 weeks.

## 4. INVESTIGATIONAL PLAN

### 4.1 Overall study design and plan

Patients will be invited to participate by their local designated pulmonary hypertension hospital. Suitable patients will be treated with Imatinib once daily for up to 24 weeks. The study is in two parts. The screening and consenting procedures are the same for Parts 1 and 2.

Part 1 will recruit 6 to 13 patients to identify the highest tolerated dose of Imatinib within the 100mg to 400mg per day range. This dose will then be used in Part 2 in a separate group of patients to assess efficacy according to the genes that relate to PDGF activity. It is necessary to find a single dose to maximise the opportunity to assess variation in response by genotype (i.e. reduce the influence of variation in exposure). It is necessary to find the highest tolerated dose to maximise the opportunity to capture a clinical response (i.e. reduce the chance of missing an effect). A starting dose of 100mg is based on experience from compassionate use; some patients, such as the case report above, have reported benefit from this dose. Doses of 200mg to 400mg daily were investigated in IMPRES (10) and many patients ended up on 200mg daily in the extension study. Our prior model of toxicity -see Section 10; skeleton- predicts that 200mg daily is likely to be the highest tolerated dose in PAH, and Part 1 is designed to explore this. A lower dose will be investigated in the efficacy phase if better tolerated.

Some patients with PAH attending the specialist clinics have both an implanted cardiopulmonary monitor and an activity monitor that permits remote sensing and a remote system for provision of daily blood pressure, oxygen saturations and body weight. These offer a particular advantage in a time of Covid-19, minimising contact with the hospital. The monitoring also offers greater oversight of the patient and closer safety monitoring. Remotely acquired patient data are reviewed twice a week via a central clinical team based in Sheffield. Many of these patients would be suitable for Part 1 of this study where the emphasis is on identifying a tolerated dose. These patients would not require a baseline RHC, as key data would be retrieved from the implanted monitor. Relevant data will be sent as de-identifiable information to the recruiting site for upload onto the study database (eCRF). Continuous monitoring data can be sent to Imperial College London (upon request) as pseudonymised (coded) data for consideration of the Trial Steering and Data Monitoring Committees, if there are any concerns regarding patients' safety. Follow-up would be scheduled by telephone or video conferencing with hospital visits being optional. Participants who consent to take part but do not have an implanted cardiac monitor will attend the hospital for a baseline RHC and then again at the end of Week 4 after starting Imatinib to assess tolerability, with telephone or video conference assessments at the end of Weeks 1, 2 and 3.

We will explore 4 doses between 100mg and 400mg of Imatinib (100mg, 200mg, 300mg and 400mg), the maximum dose escalation between patients is limited to 100mg to protect against rapid escalation to an intolerable dose. The first patient will receive Imatinib 100mg (x1) once daily. Subsequent patients will enter the trial at no less than 4 week intervals. The second patient will receive a dose of Imatinib based on the experience of the previous patient(s) at 4 weeks; if 100mg is tolerated, the second patient will receive 200mg. The third patient will receive a dose of Imatinib based on the experience of the previous patients at 4 weeks, using a statistical model to identify the most likely highest tolerated dose. This will be between 100mg and 300mg daily. The statistical model will be used to choose the dose for the fourth patient up to a maximum of 400mg daily.

By definition, the highest tolerated dose is reached in this trial when there is a 20% probability that a patient will not be able to continue the drug for 5 consecutive days. All patients who are tolerating Imatinib will be asked to continue on their assigned dose to the end of week 24 and their clinical response will be measured by the change in PVR (measured through a follow-up RHC). If a patient becomes intolerant of Imatinib, they will be allowed to reduce the dose guided by their physician; if they are unable to tolerate 100mg daily, they are withdrawn from the study.

The objective of Part 2 is to define the clinical response to Imatinib after 24 weeks treatment with the highest tolerated dose by measuring the change in PVR through a follow-up RHC. Some patients from Part 1 will have continued on the highest tolerated dose to 24 weeks and so be evaluable for additional efficacy at this dose. Part 2 will continue recruitment of additional patients to a total of 34 evaluable patients; all patients in Part 2 will receive the highest tolerated dose (defined in Part 1) for 24 weeks. Patients will be monitored for safety at the end of Weeks 4, 8, 12 and 24, with telephone assessments at the end of Weeks 16, 20, and 28. If a patient becomes intolerant of Imatinib, they will be allowed to continue with a reduced dose or alterative treatment as guided by their NHS specialist physician but they will be withdrawn from the study. Beyond that point, we will not be collecting new data from those patients. The clinical response of all patients who complete 24 weeks on the highest tolerated dose of Imatinib will be measured by change in PVR from baseline at Week 24. The primary efficacy endpoint is a binary variable: for patients with a baseline PVR >1000 dynes·s·cm$^{-5}$, success is defined by an absolute reduction in PVR of ≥300 dynes·s·cm$^{-5}$at 24 weeks; for patients with a baseline PVR ≤1000 dynes·s·cm$^{-5}$, success is a 30% reduction in PVR at 24 weeks.

An interim analysis is planned after 13 patients have completed the 24 week treatment on the highest tolerated dose. If there are 1 or no responses in these patients, the study will be stopped. Otherwise, additional patients will be recruited for a total of 34 evaluable patients. The null hypothesis will be rejected if 7 or more responses are observed in the 34 patient cohort. Blood levels of Imatinib will be measured in each patient at baseline and at the end of Weeks 4 and 24. A trough level (noting the time for last dose) will be measured at the assessment on Week 4 and that on Week 24. Dose-limiting toxicity is defined as discontinuation of treatment for more than 5 consecutive days due to Grade 2 or above Adverse Events defined by the NCI Common Terminology Criteria for Adverse Events (version 5.0, 2017), adapted for this study - see Appendix 2. The criteria have been adapted by removing reference to pulmonary hypertension as an adverse event (since this is the indication for this study).

The most common recognised side effects of Imatinib include:
- Muscle cramps
- Joint pains
- Nausea and vomiting
- Swelling of the ankles and face
- Skin rash
- Loose stools
- Unusual bleeding or bruising
- Fatigue

Leg oedema and weight gain can also indicate deterioration in PAH due to right heart failure, rather than Imatinib toxicity. At the discretion of the study physician, a trial of increased dose of diuretics is permitted to help reduce the oedema and weight gain. If the oedema and weight gain are judged to be due to Imatinib toxicity, the dose of Imatinib may be reduced or the drug stopped and the patient is managed according to best clinical practice.

If a patient is unable to continue with the prescribed dose of Imatinib to 24 weeks, they will be withdrawn from the study. These patients may continue with a lower dose of Imatinib or discontinue the drug under the guidance of their local NHS specialist physician.

### 4.2 Discussion of study design

The first part of this study is open label, single dose escalation. The aim is to find a safe dose that is tolerated in the target patient population. Doses will be escalated slowly between patients, guided by a statistical model and an independent Data Monitoring Committee. The starting and maximum doses (i.e. dose range) to be investigated is based on a Phase 3 study [IMPRES (10)] and clinical experience. Our planned sample size, as is generally the case for Phase I trials, is dictated by practical constraints, even more so when the condition being treated is rare (as is PAH). However, simulation studies of the

most likely scenario based on the IMPRES trial (10) and expert opinion have shown that a sample size of 13 resulted in a 90% chance of recommending a dose with an associated toxicity within 10% of the target toxicity level (20%) in the most likely scenario. We will be updating the skeleton graph -see Section 10- and revising the number of patients required overall after each patient is treated. We will also consider criteria for extending the sample size if needed and feasible.

The recruitment rate is dictated by the time to assess safety and tolerability at each dose. The decision to escalate dose, identify the highest tolerated dose, and Go/No-Go decision for Part 2 will be made by the independent Trial Safety and Data Monitoring Committees, comprising of lead clinician(s), and oncologist with expertise in the use of Imatinib and a Senior Trial Statistician.

### 4.3 Selection of study population

Patients participating in this study are adult males and females with symptomatic PAH as defined by the eligibility criteria below. No deviations will be made to the eligibility criteria.

PAH is a rare condition. Patients with PAH attend one of 8 UK specialist centres in the UK for diagnosis and management decisions. Currently these centres have recruited over 650 patients with well-defined PAH to a cohort study. These patients are in regular follow-up and have been consented to recall to future clinical studies. We anticipate recruiting 1 to 2 patients per month. This is based on a recent academic-led study of tocilizumab in PAH and the expertise of the recruiting centres in clinical studies in PAH.

The Covid-19 pandemic has changed clinical practice, with many more consultations taking place by telephone or video conferencing. This is facilitated by the use of remote monitoring – See Figure 5. The PAH cohort has an expanding number of patients with an implanted CardioMEMS$^{TM}$ Heart Failure system and a Fitbit activity monitor as part of the FIT-PH study (Feasibility of Novel Clinical Trial Infrastructure, Design and Technology for Early Phase Studies in Patients with Pulmonary Hypertension; Ethics approval 19/YH/0354). These have been under follow-up for a mean of 7 months and are stable.

This patient group provide daily data about their health (level of physical activity, day and night cardiac rate and rhythm, heart rate variability, thoracic impedance, respiratory rate, systemic blood pressure, oxygen saturations, body weight, pulmonary artery pressure, cardiac output and stroke volume) to a hospital-based monitoring team and benefit from close contact with the specialist team while reducing travel to the hospital. Remote systemic blood pressure, oxygen saturations and body weight are provided through the FDA/CE approved Cordella Heart Failure System (24). The protocol of the FIT-PH study observational study permits evaluation of therapeutic interventions. This group of patients would be suitable for assessing the tolerability of Imatinib in Part 1 of the PIPAH study. They can provide valuable additional information on the haemodynamic response to Imatinib that cannot be obtained by other means as well as objective activity data to enable interpretation of self-reported information.

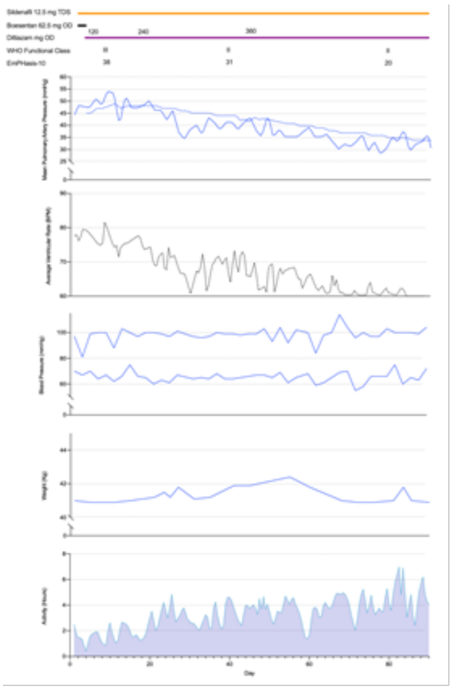

**Figure 5 -** Remote monitoring of a 55-year old patient with PAH over 90 days reporting mean pulmonary artery pressure (mmHg), heart rate (bpm), blood pressure (mmHg), body weight (kg), and physical activity (in hours). Note response to increased dose of Diltiazem (120 to 360 mg).

#### 4.3.1 Inclusion criteria

**1.** Subjects aged between 18-75 years old

**2.** PAH which is idiopathic, heritable or associated with anorexigens

**3.** Subjects willing to be genotyped for genes that influence PDGF activity

**4.** Resting mean pulmonary artery pressure >25 mmHg, Pulmonary capillary wedge pressure ≤15 mmHg, PVR >5 wood units, and normal or reduced cardiac output, as measured by right heart catheterisation (RHC) at entry

**5.** Six-minute walking distance >50m at entry

**6.** Stable on an unchanged PAH therapeutic regime comprising at least 2 therapies licensed for PAH (any combination of endothelin receptor antagonist, phosphodiesterase inhibitor or prostacyclin analogue) for at least 1 month prior to screening

**7.** Able to provide written informed consent prior to any study mandated procedures

**8.** Contraception: Women of childbearing potential are eligible to participate after a negative highly sensitive pregnancy test if they are taking a highly effective method of contraception during treatment and until the end of relevant systemic exposure.

Fertile males who make use of condom and contraception methods during treatment and until the end of relevant systemic exposure in women of childbearing potential.

Women of childbearing potential and fertile men* are eligible to participate, if they agree to use highly effective contraceptive methods with typical-use failure rate <1% per year, when used consistently and correctly. Such methods include:

- sexual abstinence
- combined (oestrogen and progestogen containing) hormonal contraception associated with inhibition of ovulation[1]:
    - o oral
    - o intravaginal
    - o transdermal
- progestogen-only hormonal contraception associated with inhibition of ovulation1:
    - o oral
    - o injectable
    - o implantable[2]
- intrauterine device (also called IUD)[2]
- intrauterine hormone-releasing system (also known as IUS)[2]
- bilateral tubal occlusion[2]
- vasectomised partner[2,3]

*Definition of fertile females (women of childbearing potential) and of fertile men:

For the purpose of this document, a woman is considered of childbearing potential, i.e. fertile, following menarche and until becoming post-menopausal unless permanently sterile. Permanent sterilisation methods include hysterectomy, bilateral salpingectomy and bilateral oophorectomy. A postmenopausal state is defined as no menses for 12 months without an alternative medical cause. A high follicle stimulating hormone (FSH) level in the postmenopausal range may be used to confirm a post-menopausal state in women not using hormonal contraception or hormonal replacement therapy. However in the absence of 12 months of amenorrhea, a single FSH measurement is insufficient. For the purpose of this document, a man is considered fertile after puberty unless permanently sterile by bilateral orchidectomy.

[1]Hormonal contraception may be susceptible to interaction with the IMP, which may reduce the efficacy of the contraception method.

[2]Contraception methods that in this context are considered to have low user dependency.

[3]Vasectomised partner is a highly effective birth control method provided that partner is the sole sexual partner of the associated female trial participant and that the vasectomised partner has received medical assessment of the surgical success.

[4]In the context of this guidance sexual abstinence is considered a highly effective method only if defined as refraining from heterosexual intercourse during the entire period of risk associated with the study treatments. The reliability of sexual abstinence needs to be evaluated in relation to the duration of the clinical trial and the preferred and usual lifestyle of the subject.

#### 4.3.2 Exclusion criteria

**1.** Unable to provide informed consent and/or are non-fluent speakers of the English language

**2.** Hypersensitivity to Imatinib or to any of the excipients

**3.** Clinically-significant renal disease (confirmed by creatinine clearance <30 ml/min per $1.73m^2$)

**4.** Clinically-significant liver disease (confirmed by serum transaminases >3 times than upper normal limit)

**5.** Patients receiving oral and/or parenteral anticoagulants*

**6.** Anaemia confirmed by haemoglobin concentration <10 g/dl

**7.** History of thrombocytopenia

**8.** Individuals known to have haemoglobinopathy sickle cell disease, thalassaemia

**9.** Hospital admission related to PAH or change in PAH therapy within 3 months prior to screening

**10.** History of left-sided heart disease and/or clinically significant cardiac disease, including but not limited to any of the following:

- a. Aortic or mitral valve disease (stenosis or regurgitation) defined as greater than mild aortic insufficiency, mild aortic stenosis, mild mitral stenosis, moderate mitral regurgitation
- b. Mechanical or bioprosthetic cardiac valve
- c. Pericardial constriction/effusion with tamponade physiology, or abnormal left atrial size.
- d. Restrictive or congestive cardiomyopathy
- e. Left ventricular ejection fraction ≤50% (measured in echocardiogram at screening)
- f. Symptomatic coronary disease
- g. Significant (2+ for regurgitation) valvular disease other than tricuspid or pulmonary regurgitation
- h. Acutely decompensated left heart failure within 1 month of screening
- i. History of untreated obstructive sleep apnoea

**11.** Evidence of significant lung disease on high-resolution CT (if available) or recent(performed within 12 months) lung function, where FEV1 <50% predicted and FVC <70% predicted, and DLCO (or TLCO) <50% predicted if any CT abnormalities; judged by the Site Physician

**12.** Patients with a history of uncontrolled systemic hypertension

**13.** Acute infection (including eye, dental, and skin infections)

**14.** Chronic inflammatory disease including HIV and Hepatitis B

**15.** Women of childbearing potential who are pregnant or breastfeeding (if applicable)

**16.** Previous intracerebral haemorrhage

**17.** Patients who have received an IMP within 5 half-lives of the last dose or 1 month (whichever is greater) before the baseline visit

**this does not apply to single daily antiplatelet treatment*

#### 4.3.3 Subject completion

Subjects will be considered complete for the purpose of this study once they have completed all procedures of the follow-up telephone assessment or unscheduled visit at the end of Week 28 (or 4 Weeks after early termination). The end of the clinical trial is defined as the last assessment of the last subject undergoing the trial.

### 4.3.4 Discontinuation criteria

It is possible that the Sponsor or the Competent Authorities request termination of the study if there are concerns about conduct or safety, or due to a change in the opinion of the Ethics Committee.

When the Sponsor is aware of information on matters concerning the quality, efficacy, and safety of the study drug, as well as other important information that may affect proper conduct of the clinical study, the Sponsor may discontinue the clinical study and send a written notice of the discontinuation along with the reasons to the Investigators. Patients will be informed that they have the right to withdraw from the study at any time for any reason, without prejudice to their medical care. The site Investigator/Chief Investigator can discontinue subjects from the study for any of the following reasons:

**1**. Occurrence of an unacceptable Adverse Event due to Grade 2 or above defined by the NCI Common Terminology Criteria for Adverse Events (version 5.0, 2017), adapted for this study -see Appendix 2.
**2**. Subject request
**3.** Subject is lost to follow-up
**4.** Administrative reasons
**5.** Development of an intercurrent illness, condition, or procedural complication, which would interfere with the subject's continued participation
**6.** Patient becomes pregnant

The site Investigator/Chief Investigator also reserves the right to discontinue subjects in the interest of subject safety and welfare. Investigators must contact all participants and the hospital pharmacy (where applicable) to notify them of the termination of the study. In cases of an early termination (discontinuation), a follow-up telephone assessment will be performed 4 weeks ± 3 days -see Appendix 1.

### 4.3.5 Subject identification and replacement policy

After informed consent is obtained, patients who are screened will be assigned a 7-digit permanent identification number (subject ID) such that all patients from each research site are given consecutive identification numbers in successive order of inclusion. The first 4 digits of the will be the designated research site ID, and the last 3 digits will be assigned at the research study centre (e.g., if the site ID is 4410, the third patient screened at 4410 site, would be given the number of 4410003).

A patient who is screened but fails to proceed into baseline assessment (e.g., because entry criteria were not met or enrolment did not occur within the specified time frame) may be considered for screening again. Re-screening will be permitted by the site Investigator on a case-by-case basis. A new informed consent form will be signed in any case of re-screening. A new subject ID will be assigned to the subject. Patients who are discontinued will be replaced. Each replacement is by a default a new subject, who will be assigned a completely new subject ID.

## 5. INVESTIGATIONAL MEDICINAL PRODUCT (IMP)

Patients who are eligible to participate in the study will receive Imatinib.

### 5.1 Imatinib

Imatinib is licensed in the UK to treat patients with certain types of cancer -see more in the British National Formulary: https://bnf.nice.org.uk/drug/imatinib.html#indicationsAndDoses. In this study, patients will be prescribed one (or a combination) of the following preparations:

| Qualitative and quantitative composition |
|---|
| Each film-coated tablet contains 100mg Imatinib (as mesilate)<br>Each film-coated tablet contains 400mg Imatinib (as mesilate) |

| Excipients, Shelf life, Nature and contents of container, Storage and disposal precautions |
|---|
| The study drug will be stored in Pharmacy. For details on contents, storage and disposal precautions - see respective Summary of Product Characteristics (SmPc) |
| **Supply** |
| Commercial supply via the NHS. Patients will be collecting the IMP at study visits, or if necessary receiving the IMP at home (dispensed by local pharmacy, shipped by the research team). |

### 5.2 IMP preparation and administration

#### 5.2.1 IMP Preparation

Film-coated tablets

#### 5.2.2. IMP Administration

Oral

### 5.3 Labelling

The study drug will be labelled by local Pharmacy under Regulation 37 of The Medicines for Human Use (Clinical Trials) Regulations 2004 in accordance with local regulatory requirements. Labelling will be done in Pharmacy.

### 5.4 Study drug accountability

Accountability for the study drug at the study site is the responsibility of the site Investigator. He/she will ensure that the study drug is used only in accordance with this protocol. Where allowed, the site Investigator may choose to assign some of the drug accountability responsibilities to a pharmacist or other appropriate individual.

Drug accountability records indicating the drug's delivery date to the site, inventory at the site, use by each subject, and end-of-study destruction and disposal of the drug, will be maintained by each clinical site. These records will adequately document that the subjects were provided the doses as specified in the protocol. Site's accountability pharmacy logs can be used to confirm if sites can destroy/dispose of unused drug in line with their local requirements.

### 5.5 Treatment assignment

Patients will receive Imatinib as a single oral daily dose. For instance, the dose for the first subject will be film-coated tablet Imatinib 100mg (x1) OD (i.e. once daily).

### 5.6 Concomitant medications

#### 5.6.1 Permitted concomitant medications

Approved endothelin receptor antagonists, phosphodiesterase-5 type inhibitors and/or prostacyclin analogues are permitted for the treatment of PAH. The dose must be stable for at least 1 month prior to entry into the study.

#### 5.6.2 Prohibited concomitant medications

Oral or parenteral anticoagulant therapy. A list of drugs that cannot be used concomitantly with Imatinib is provided in the SmPC.

## 6. VISIT AND ASSESSMENT SCHEDULE

For a tabulated summary of all visits and assessments see the Schedule of Events, Appendix 1.

### 6.1 Screening assessment (Before Week 0)

The screening assessment should be performed no more than 28 days prior to the baseline visit. Participants' eligibility will be determined using data collected during their routine hospital appointment:

- Review of inclusion/exclusion criteria
- Obtain written informed consent*
- Demographics
- Medical and medication history (incl. smoking and alcohol history)
- Physical examination
- Concomitant medications
- Vital signs: (i) Resting supine blood pressure (BP), (ii) pulse measurement, (iii) temperature, (iv) pulse oximetry (incl. respiratory rate), (v) height, and (vi) weight
- World Health Organisation (WHO) Functional Class
- Six-minute walk test (6MWT) and Borg dyspnoea index
- Echocardiogram
- Mouth swab sample for genotyping
- Blood samples for haematology, clinical chemistry, and serum pregnancy test whereas indicated (for women of childbearing potential)
- Brain MRI scan** (or brain CT scan if patient unable to tolerate/contraindicated to have MRI)

**a signed and dated informed consent form will be obtained before screening procedures commence. A hard copy of the participant information sheet and signed consent form will be given to the patient.*

***can be performed on a separate day ± 3 days apart from the original visit date, as needed.*

### 6.2 Baseline assessment (Week 0)

The patient will undergo the following procedures:

- Review of test results and reports with the Study Physician
- Physical examination
- Concomitant medications
- Document AEs
- Vital signs: (i) Resting supine BP, (ii) pulse measurement, (iii) temperature, (iv) pulse oximetry (incl. respiratory rate), and (v) weight
- Electrocardiogram (ECG)
- 6MWT and Borg dyspnoea index
- Right heart catheterisation (unless the patient has an implanted CardioMEMS$^{TM}$ device)
- QoL questionnaire
- Blood samples for haematology, clinical chemistry (incl. virology), and serum pregnancy test whereas indicated (for women of childbearing potential)
- Blood sample for Imatinib levels (25)
- Research blood sampling

**can be performed on a separate day ± 3 days apart from the original visit date, as needed.*

Following completion of the above procedures, each patient will receive their first dose of Imatinib.

### 6.3 Telephone assessment (Week 1 ± 3 days)

Patients will receive a telephone call at the end of Week 1 to check concomitant medications, and for the occurrence of AEs. Patients will be asked to weigh themselves and report their weight and any ankle swelling.

### 6.4 Telephone assessment (Week 2 ± 3 days)

Patients will receive a telephone call at the end of Week 2 to check concomitant medications, and for the occurrence of AEs. Patients will be asked to weigh themselves and report their weight and any ankle swelling.

### 6.5 Telephone assessment (Week 3 ± 3 days)

Patients will receive a telephone call at the end of Week 3 to check concomitant medications, and for the occurrence of AEs. Patients will be asked to weigh themselves and report their weight and any ankle swelling.

### 6.6 Assessment on Week 4 (Week 4 ± 3 days)

The patient will be invited to attend their local hospital or a home assessment will be made at the end of Week 4 in order to undergo the following procedures:

- Physical examination
- Concomitant medications
- Document AEs
- Vital signs: (i) Resting supine BP, (ii) pulse measurement, (iii) temperature, (iv) pulse oximetry (incl. respiratory rate), and (v) weight
- ECG
- WHO Functional Class
- 6MWT and Borg dyspnoea index
- QoL questionnaire
- Blood samples for haematology and clinical chemistry, and serum pregnancy test whereas indicated (for women of childbearing potential)
- Blood sample for Imatinib levels
- Research blood sampling
- Review of the diary, Imatinib collection and reconciliation

### 6.7 Assessment on Week 8 (Week 8 ± 3 days)

The patient will be invited to attend their local hospital or a home assessment will be made at the end of Week 8 in order to undergo the following procedures:

- Physical examination
- Concomitant medications
- Document AEs
- Vital signs: (i) Resting supine BP, (ii) pulse measurement, (iii) temperature, (iv) pulse oximetry (incl. respiratory rate), and (v) weight
- ECG
- WHO Functional Class
- 6MWT and Borg dyspnoea index
- Blood samples for haematology and clinical chemistry, and serum pregnancy test whereas indicated (for women of childbearing potential)
- Review of the diary, Imatinib collection and reconciliation

### 6.8 Assessment on Week 12 (Week 12 ± 3 days)

The patient will be invited to attend their local hospital or a home assessment will be made at the end of Week 12 in order to undergo the following procedures:

- Physical examination
- Concomitant medications
- Document AEs
- Vital signs: (i) Resting supine BP, (ii) pulse measurement, (iii) temperature, (iv) pulse oximetry (incl. respiratory rate), and (v) weight
- ECG

- WHO Functional Class
- 6MWT and Borg dyspnoea index
- Blood samples for haematology and clinical chemistry, and serum pregnancy test whereas indicated (for women of childbearing potential)
- Review of the diary, Imatinib collection and reconciliation

**6.9 Telephone assessment (Week 16 ± 3 days)**

Patients will receive a telephone call at the end of Week 16 to check concomitant medications, and for the occurrence of AEs. Patients will be asked to weigh themselves and report their weight and any ankle swelling. For women of childbearing potential: A urine pregnancy test will be performed at home. The test and guidance will be given to the patient at the hospital at one of the previous assessments. A telephone call will be performed within 72 hours of the urine test date to enquire about the results.

**6.10 Telephone assessment (Week 20 ± 3 days)**

Patients will receive a telephone call at the end of Week 20 to check concomitant medications, and for the occurrence of AEs. Patients will be asked to weigh themselves and report their weight and any ankle swelling. For women of childbearing potential: A urine pregnancy test will be performed at home. The test and guidance will be given to the patient at the hospital at one of the previous assessments. A telephone call will be performed within 72 hours of the urine test date to enquire about the results.

**6.11 End of treatment assessment (Week 24 ± 3 days)**

The patient will be invited to attend their local hospital at the end of Week 24 (or earlier if early termination of imatinib) in order to undergo the following procedures (in the case of patients with an implanted CardioMEMS$^{TM}$ device, a home assessment will be offered):

- Physical examination
- Concomitant medications
- Document AEs
- Vital signs: (i) Resting supine BP, (ii) pulse measurement, (iii) temperature, (iv) pulse oximetry (incl. respiratory rate), and (v) weight
- ECG
- WHO Functional Class
- 6MWT and Borg dyspnoea index
- Right heart catheterisation*(unless the patient has an implanted CardioMEMS$^{TM}$ device)
- Echocardiogram
- QoL questionnaire
- Blood samples for haematology and clinical chemistry, and serum pregnancy test whereas indicated (for women of childbearing potential)
- Blood sample for Imatinib levels
- Research blood sampling
- Review of the diary, Imatinib collection and reconciliation

**can be performed on a separate day ± 3 days apart from the original visit date, as needed.*

**6.12 Follow-up telephone assessment (Week 28, or 4 weeks after early termination ± 3 days)**

At the end of the study, clinical follow-up will continue via the local NHS-designated pulmonary hypertension clinics. The patients that have completed the 24-week treatment period on Imatinib may continue with the study drug under the supervision of their local NHS specialist physician. Those who discontinue Imatinib will be reviewed by their local physician for alternative treatment. In all cases, patients will receive a telephone call from the study physician/clinical study team once they have been off study drug for 4 weeks (±3 days) to check concomitant medications, and for the occurrence of AEs. Patients will be also asked to weigh themselves and report their weight and ankle swelling. For women of childbearing potential: A urine pregnancy test will

be performed at home. A telephone call will be performed within 72 hours of the urine test date to enquire about the results.

### 6.13 Unscheduled visit (when needed)

This type of visit can be performed at any time during the study, when the patient/study physician finds it necessary. For example, an unscheduled visit can be performed if the patient experiences discomfort at home due to ankle swelling that needs further investigation. In every unscheduled visit, the patient will undergo the following procedures:

- Physical examination
- Concomitant medications
- Document AEs
- Vital signs: (i) Resting supine BP, (ii) pulse measurement, (iii) temperature, (iv) pulse oximetry (incl. respiratory rate), and (v) weight
- ECG*
- Echocardiogram*
- WHO Functional Class*
- 6MWT and Borg dyspnoea index*
- Blood samples for haematology and clinical chemistry*

**if clinically required*.

## 7. STUDY ASSESSMENTS

### 7.1 Physical examination, medical and medication history

Physical examinations will be performed to ensure suitability according to the inclusion and exclusion criteria at screening and to document the health status at the time-points specified in the Schedule of Events - see Appendix 1. The physical examination (incl. vital signs) is a routine medical examination. A medical history will be recorded at screening only. The medical history will elicit information concerning existing medical conditions, major illnesses, and related surgical procedures. Any prescribed or over-the-counter medications that the subject received within the past 30 days should be recorded on the case report form (CRF). Medication prescribed for the treatment of PAH for 2 months prior to enrolment should be recorded on the CRF. Subjects will be instructed to notify the study physician before beginning new prescribed or over-the-counter medications.

### 7.2 WHO functional class

Functional assessment of PAH will be made according to the WHO classification system (26).

| | |
|---|---|
| Class I: | Patients with PAH without limitation of physical activity. Ordinary physical activity does not cause increased dyspnoea or fatigue, chest pain, or near syncope. |
| Class II: | Patients with PAH resulting in slight limitation of physical activity. No discomfort at rest. Normal physical activity causes increased dyspnoea or fatigue, chest pain, or near syncope. |
| Class III: | Patients with PAH resulting in marked limitation of physical activity. There is no discomfort at rest. Less than ordinary activity causes increased dyspnoea or fatigue, chest pain, or near syncope. |
| Class IV: | Patients with PAH with inability to carry out any physical activity without discomfort. Indications of manifest right heart failure. Dyspnoea and/or fatigue may even be present at rest. Discomfort is increased by the least physical activity. |

### 7.3 Vital signs

Systolic and diastolic systemic BP will be measured by means of either a standard manual or an automatic BP measuring device (cuff method). For each subject, the same method should be used during the entire study, and the type of device used should be recorded on the CRF. The same arm will be used for each measurement of BP, and BP will be measured after 5 minutes seated. Heart rate (HR), respiratory rate, and oxygen saturation will be measured by pulse oximetry after the subject has been at rest for at least 5 minutes.

### 7.4 Borg Dyspnoea Index

| | |
|---|---|
| 0 | Nothing at all |
| 0.5 | Very, very slight (just noticeable) |
| 1 | Very slight |
| 2 | Slight |
| 3 | Moderate |
| 4 | Somewhat severe |
| 5 | Severe |
| 6 | |
| 7 | Very severe |
| 8 | |
| 9 | Very, very severe (almost maximal) |
| 10 | Maximal |

### 7.5 Six-minute walk distance (6MWD):

Distance walked during an unencouraged 6MWT conducted according to American Thoracic Society guidelines (6). This is a standard tool for the study of functional capacity in PAH patients and is primarily determined by cardiac output and hence right ventricular function. We are aware that a possible increase in haemoglobin might be a confounding factor, improving 6MWD not because of the increase in cardiac output but because of increased oxygen delivery. This is the rationale behind assessing exercise haemodynamics. We will interpret the 6MWD in the context of the overall results.

### 7.6 Laboratory measurements

#### 7.6.1 Routine laboratory measurements

Routine clinical laboratory parameters (haematology, clinical chemistry) will be analysed by local accredited hospital laboratories. Tests may vary slightly depending on the availability of local hospital assays and so some flexibility with regards to the specific tests will be tolerated.

Routine laboratory tests include the following:

| | |
|---|---|
| **haematology** | White blood cell count (WBC) and differentials (neutrophils, lymphocytes, monocytes, eosinophils, and basophils), red blood cell count (RBC), platelet count, haemoglobin (Hb), haematocrit level (Hct), mean cell volume (MCV), mean cell haemoglobin level (MCH), mean cell haemoglobin concentration (MCHC), and reticulocytes, red cell distribution width (RDW) |
| **clinical chemistry** | Albumin, total bilirubin, urea, creatinine, eGFR*, glucose, total protein, C reactive protein (CRP), urate, serum electrolytes [calcium (Ca), chloride (Cl), sodium (Na), phosphate (P), potassium (K), magnesium (Mg)], bicarbonate ($HCO_3$), aspartate aminotransferase (AST), alanine aminotransferase (ALT), $\gamma$-glutamyl-transferase ($\gamma$-GT/gamma-GT), alkaline phosphatase (ALP), lactate dehydrogenase (LDH/LD), creatine kinase (CK), serum iron, thyroid stimulating hormone (TSH), free thyroxine (T4); also, brain natriuretic peptide (BNP) or N-terminal (NT)-pro hormone BNP (proNT-BNP) |

| | |
|---|---|
| | Virology**:<br>HIV, Hepatitis B virus core Ab, Hepatitis B virus surface Ab, and Hepatitis B virus surface Ag |
| **serum pregnancy test** | beta human chorionic gonadotropin (β-hCG) |

**eGFR using CKD-EPI will be calculated in ml/min per 1.73m$^2$ via the following weblink:* https://qxmd.com/calculate/calculator_251/egfr-using-ckd-epi

***virology tests to be performed at screening only.*

### 7.6.2 Imatinib assay

Blood samples will be collected for measuring the blood levels of the study drug at baseline (Visit 2), at assessment on Week 4 and end of treatment (assessment on Week 24 or earlier, if early termination). Samples will be pre-processed at each site and stored locally in -80°C laboratory freezers. Frozen samples will be shipped to the central laboratory contracted to conduct further analyses to measure the drug concentration in the blood (25). Detailed instructions for the preparation, labelling, storage and shipment of these samples, as well as reporting results back to Imperial College London can be found in the relevant section of the laboratory manual.

### 7.6.3 Genotyping

A mouth swab sample (saliva and buccal cells of maximum 2ml) will be collected for each individual participant at screening. Samples may be temporarily stored at the recruiting site. Samples will be shipped to the accredited laboratory contracted to conduct central laboratory DNA testing. These samples will be analysed by polymerase chain reaction (PCR) using appropriate primers for single nucleotide polymorphisms (SNPs) related to PDGF activity. Sample-related data (labelling information) shared with Imperial College will be pseudonymised (coded). Details on preparation, labelling, storage and shipment of these samples can be found in the relevant  section of the laboratory manual.

### 7.6.4 Research blood samples

Blood samples will be collected for measuring proNT-BNP levels and for future research studies. Research blood samples will be processed at the site of collection to extract plasma serum and cells, which will be stored locally in -80°C laboratory freezers. Frozen samples will be shipped to the central laboratory at Imperial College London (Pulmonary Hypertension Biobank), where they will be deposited for future research focused on PAH and/or Imatinib studies. Data shared with Imperial College London to accompany research blood samples will be pseudonymised (coded). For selected measurements (e.g. proteomic analyses), samples or products of them shall be sent to accredited central laboratory/laboratories overseas (e.g. USA) contracted to perform proteomic analysis with regards to the PIPAH study. Any data shared with these third parties will be anonymised. Details on preparation, labelling, storage and shipment of these samples can be found in the relevant section of the laboratory manual.

### 7.6.5 Volume of blood collection

The approximate blood volume collected from each individual during all scheduled visits will be:

| **assessment** | **ml/collection** | **number of collections** | **total (ml)** |
|---|---|---|---|
| haematology | 3.0 | 6 | 18.0 |
| clinical chemistry | 10.0 | 6 | 60.0 |
| serum pregnancy test* | 1.5 | 6 | 9.0 |
| Imatinib assay | 5.0 | 3 | 15.0 |
| research blood | 10.0 | 3 | 30.0 |
| | | **total volume:** | 123.0 ml<br>(132.0 ml)* |

**for women of childbearing potential*

### 7.7 QoL questionnaire

The self-reported QoL questionnaire (23) for patients with PAH will be used at baseline (Week 0), assessment on Week 4 and end of treatment (assessment on Week 24 or earlier, if early termination). Individual scores will be calculated at each time-point.

### 7.8 Electrocardiogram (ECG) / Echocardiogram

A single 12-lead ECG will be conducted at baseline assessment, and at the following assessments on Weeks 4, 8, 12 and 24 (and at any unscheduled assessment, as needed). A qualified physician will be responsible for interpreting the ECGs. Any ECG finding that is judged by the site investigator as a clinically significant change (worsening) will be considered an Adverse Event.

An echocardiogram will be conducted at screening and at the end of treatment (assessment on Week 24 or earlier, if early termination); it may be also performed at any unscheduled assessment (as needed). A qualified physician will be responsible for interpreting the echocardiogram. Any echocardiogram finding that is judged by the site investigator as a clinically significant change (worsening) will be considered an Adverse Event.

### 7.9 Brain imaging

A conventional brain magnetic resonance imaging (MRI) scan will be conducted at screening (Visit 1) to exclude previous intracerebral haemorrhage. Where MRI is contra-indicated (e.g. due to metal implants and/or claustrophobia), participants will be asked to undergo a CT scan of the head at screening (Visit 1) to exclude intracerebral haemorrhage. The maximum total radiation dose from the CT scan will be approximately 2 mSv. The brain MRI scan will always be the first choice. Imaging will take place at each hospital site. All brain scans will be reported routinely. Reports will be documented on eCRFs for safety and monitoring purposes. Any incidental finding will be considered an Adverse Event.

### 7.10 Right heart catheterisation (RHC)

RHC will be conducted at screening (Visit 1) and at the end of Week 24 in patients who do not have an implanted CardioMEMS™ device. A qualified physician will be responsible for interpreting the RHC results. Any RHC finding that is judged by the site investigator as a clinically significant change (worsening) will be considered an Adverse Event. In a RHC procedure the catheter passes through the veins to the right side of the heart. Fluoroscopic X-ray images are used to guide the tip of the catheter up to the heart. A special type of dye called contrast medium is injected through the catheter and X-ray images (angiograms) are taken. The contrast medium is visible on the angiograms, showing the blood vessels that the contrast travels through and clearly highlights any blood vessels that are narrowed or blocked.

The amount of radiation used during the procedure depends on the complexity of the procedure but is approximately equivalent to about 7mSv effective dose, which is equivalent to about 3 years of natural background radiation. The maximum total radiation dose from the CT head scan (see above) and RHC procedures involved in this study is about 16 mSv. This is equivalent to approximately 7 years of natural background (2.3mSv/year) radiation. The risk of developing cancer as a consequence of taking part in this study is about 0.08 % (1 in 1250), which is low. For comparison, the natural lifetime cancer incidence in the general population is about 50%.

### 7.11 Remote monitoring and management system of haemodynamic data

The CardioMEMS™ Heart Failure System (27) provides pulmonary artery hemodynamic data used for monitoring and management of Heart Failure patients. The system includes an implantable wireless sensor with delivery catheter, a remote monitoring electronics system, and a System database.

The wireless sensor is implanted into the distal pulmonary artery. Once implanted, the CardioMEMS Pulmonary Artery Sensor provides non-invasive hemodynamic data that is collected in the System database. The dataset includes details on the pulmonary artery pressure waveform, systolic, diastolic, and mean

pulmonary artery pressure and cardia rhythm (heart rate). This hemodynamic data is transferred online to a secure website that serves as the System database, so that monitoring information is available at all times.

**7.12 Implementation of measures against Covid-19**
This study is conducted in accordance with Covid-19 Guidance from Public Health England and Public Health Scotland, Infection Prevention and Control (IPC) Recommendations, and local implementations of all involved NHS partner sites as well as the affiliated laboratories and clinical facilities where this study shall take place. Local rules and recommendations apply to all study participants and all clinical and non-clinical personnel involved for the conduct of the study in the UK. The above should read in conjunction with current NICE Covid-19 guidelines and relevant regulations and standard operating procedures. Recommendations are not mandatory and allow for local modification and adaptation.

## 8. PHARMACOVIGILANCE

### 8.1 Definitions

**Adverse Event (AE):** Any untoward medical occurrence in a patient or clinical trial subject administered a medicinal product and which does not necessarily have a causal relationship with this treatment. *An AE can therefore be any unfavourable and unintended sign (including an abnormal laboratory finding), symptom, or disease temporally associated with the use of an IMP, whether or not considered related to the IMP*.

**Adverse Reaction (AR):** All untoward and unintended responses to an IMP related to any dose administered. *All AEs judged by either the reporting Investigator or the Sponsor as having reasonable causal relationship to a medicinal product qualify as ARs. The expression reasonable causal relationship means to convey in general that there is evidence or argument to suggest a causal relationship*.

**Unexpected Adverse Reaction:** An AR, the nature or severity of which is not consistent with the applicable product information [e.g. Investigator's Brochure for an unapproved investigational product or summary of product characteristics for an authorised product]. *When the outcome of the AR is not consistent with the applicable product information this AR should be considered as unexpected. Side effects documented in the summary of product characteristics which occur in a more severe form than anticipated are also considered to be unexpected*.

**Serious Adverse Event (SAE)** or **Serious Adverse Reaction (SAR):** Any untoward medical occurrence or effect that at any dose:

- **results in death**
- **is life-threatening -** *refers to an event in which the subject was at risk of death at the time of the event; it does not refer to an event which hypothetically might have caused death if it were more severe.*
- **requires hospitalisation, or prolongation of existing inpatients' hospitalisation**
- **results in persistent or significant disability or incapacity**
- **is a congenital anomaly or a defect**

Medical judgement should be exercised in deciding whether an AE/AR is serious in other situations. Important AE/ARs that are not immediately life-threatening OR do not result in death or hospitalisation but may jeopardise the subject or may require intervention to prevent one of the other outcomes listed in the definition above, should also be considered serious.

**Suspected Unexpected Serious Adverse Reaction (SUSAR):** Any suspected AR related to an IMP that is both unexpected and serious.

### 8.2 Assigning causality

Each AE must be assessed by the site Investigator as to whether or not there is a reasonable possibility of a causal relationship to the study drug. The assignment of the causality should be made by the site Investigator

responsible for the care of the participant using the definitions below. If any doubt about the causality exists the site Investigator should inform the study coordination centre who will notify the Chief Investigator.

In the case of discrepant views on causality between the site Investigator, Chief Investigator and others, all parties will discuss the case. In the event that no agreement is made, the Medicines and Healthcare Products Regulatory Agency (MHRA) will be informed of both points of view.

### 8.2.1 Causality description

Most AEs and drug ARs that occur in this study, whether they are serious or not, will be expected treatment-related toxicities due to the drugs used in this study. The assignment of the causality should be made by the site Investigator responsible for the care of the participant using the definitions in the table below.

| Relationship | Description |
|---|---|
| **Unrelated** | There is no evidence of any causal relationship |
| **Unlikely** | There is little evidence to suggest there is a causal relationship (e.g. the event did not occur within a reasonable time after administration of the trial medication). There is another reasonable explanation for the event (e.g. the participant's clinical condition, other concomitant treatment). |
| **Possible** | There is some evidence to suggest a causal relationship (e.g. because the event occurs within a reasonable time after administration of the trial medication). However, the influence of other factors may have contributed to the event (e.g. the participant's clinical condition, other concomitant treatments). |
| **Probable** | There is evidence to suggest a causal relationship and the influence of other factors is unlikely. |
| **Definitely** | There is clear evidence to suggest a causal relationship and other possible contributing factors can be ruled out. |
| **Not assessable** | There is insufficient or incomplete evidence to make a clinical judgement of the causal relationship. |

## 8.3 Assigning severity

For each AE, intensity should be defined according to the following criteria:

| | |
|---|---|
| **Mild** | Awareness of sign or symptom, but easily tolerated |
| **Moderate** | Discomfort enough to cause interference with normal daily activities |
| **Severe** | Inability to perform normal daily activities |
| **Life threatening or disabling** | Immediate risk of death from the reaction as it occurred |
| **Death** | The event resulted in death |

## 8.4 AE reporting procedures

### 8.4.1 Screening period

SAEs occurring between signing the informed consent form and study drug initiation are only required to be reported if they are considered by the site Investigator to be related to study-mandated procedures.

### 8.4.2 Treatment period

All AEs should be reported. Depending on the nature of the event the reporting procedures below should be followed. Any questions concerning AE reporting should be directed to the study coordination centre in the first instance.

#### 8.4.2.1 Non serious AR/AEs

All such toxicities, whether expected or not, should be recorded in the AEs section of the CRF.

#### 8.4.2.2 Serious AR/AEs

All SAEs must be reported to the study coordination centre within 24 hours of the site Investigator being made aware of the event. The site Investigator should complete the SAE form and send it to the study coordination centre together with relevant treatment forms and anonymised copies of all relevant investigations. Follow-up information about a previously reported SAE must be reported within 24 hours of receiving it.

However, relapse and death due to pulmonary hypertension, and hospitalisations for elective treatment of a pre-existing condition do not need reporting as SAEs.

### 8.5 Reporting to the Regulatory Authorities

The Sponsor has delegated the responsibility for notifying the MHRA and REC of all SUSARs occurring during the study to the Chief Investigator. All SAEs and SUSARs must be reported in accordance with local regulatory guidelines:

*Life threatening SUSARs should be reported to the MHRA and REC n*o later than **7 days** after the Chief Investigator has first knowledge of the minimum criteria for expedited reporting. Further relevant information should be given within a further 8 days.

*Non-fatal and non-life threatening SUSARs should be reported to the MHRA and REC n*o later than **15 days** after the Chief Investigator has first knowledge of the minimum criteria for expedited reporting. Further relevant information should be given as soon as possible.

An annual safety report will be submitted on the anniversary of the Clinical Trial Application to the MHRA and REC.

### 8.6 Reporting to the Sponsor

All SAEs and SUSARs must be forwarded the Imperial College Joint Research Office within 24 hours of the site Investigator learning of its occurrence.

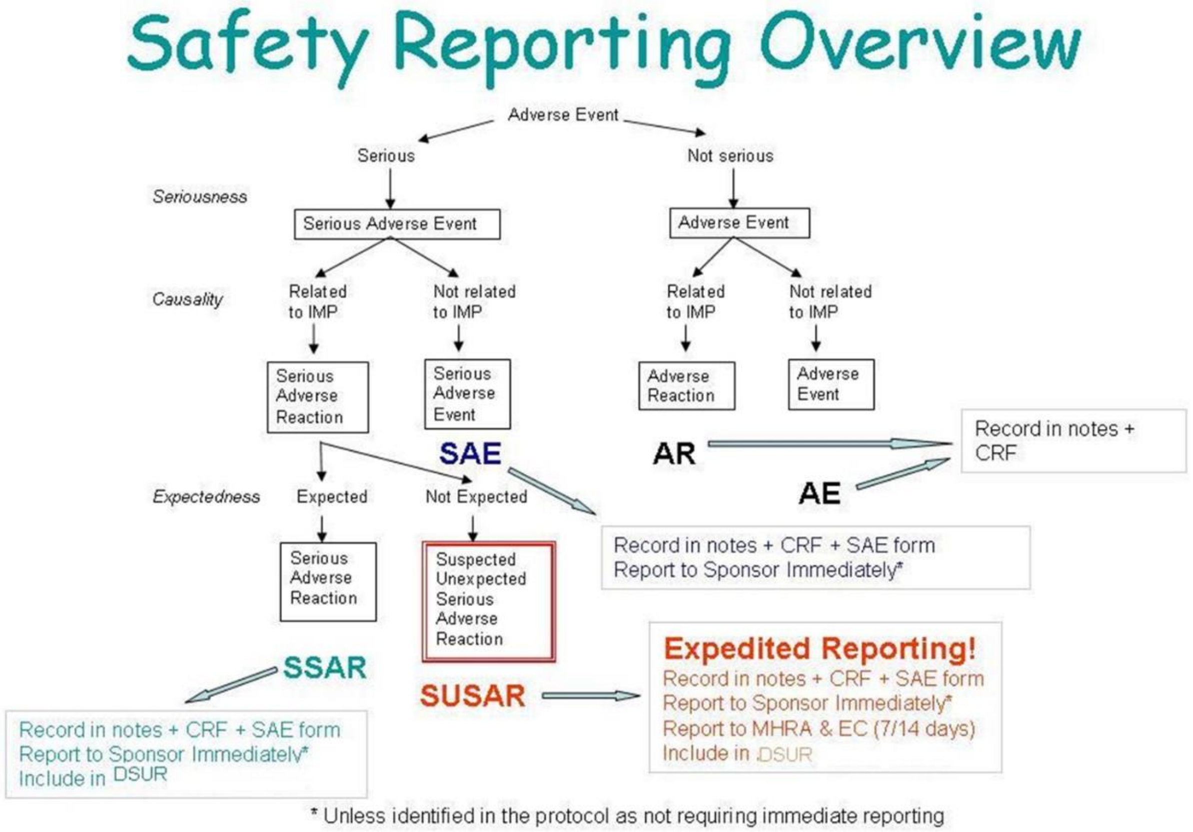


**Contact details for reporting SAEs and SUSARs**
**jrco.ctimp.team@imperial.ac.uk**
**Chief Investigator: m.wilkins@imperial.ac.uk**
**Fax: not applicable Attention to: Professor Wilkins / Dr Roussakis**
**Please send SAE forms to: jrco.ctimp.team@imperial.ac.uk cc'ing pipah@ic.ac.uk**
**Tel: 02033138070 (Mon to Fri 09.00 – 17.00)**

### 8.7 Follow-up of AEs

All AEs that are still ongoing at the end of the study will be followed up until resolution.

## 9. DOCUMENTATION OF DATA

### 9.1 Data collection

The data collection tool for this study will be electronic CRF (eCRFs). Paper CRFs may be completed for each subject. eCRFs and paper CRFs and will contain study data which are verifiable to the source data (i.e., original recordings, laboratory reports, and subject records). In addition, all source data should be attributable (signed and dated). Only the site Investigator and authorised co-workers are entitled to make entries on the eCRFs and CRFs. Concomitant medications may be entered as they appear in the participant's record or as per local standards (generic or trade names may be entered). It is the responsibility of the site Investigator to ensure that the eCRFs and CRFs are kept up-to-date so that they always contain the latest observations on the subjects enrolled.

Pathology and imaging results outside the normal range will be commented upon by the site Investigator. Other data than those requested by this protocol may be recorded as “additional data” in the comments section of the eCRF/CRF; the clinical significance of any additional data should be described. Subjects’ data will be stored in a validated database, developed and maintained by the Imperial College Trials Unit.

### 9.2 Data monitoring

The site Investigator is responsible for ensuring that the study is monitored appropriately in order to ensure compliance with GCP and local regulatory guidelines. Monitoring will organised by the Sponsor for all sites. The monitor will check the completeness of medical records, verify the accuracy of entries in the eCRFs/CRFs (source worksheets), and ensure adherence to the protocol and compliance with local regulatory requirements.

## 10. STATISTICS AND DATA ANALYSIS

### Part 1 - Dose finding phase

The highest tolerated dose of Imatinib will be determined using a Bayesian continual reassessment method. An initial dose/toxicity skeleton was generated based on a one-parameter power model and a prior guess of toxicities based on expert opinion and data from the IMPRES trial (10) - see Figure 6. Patients will be recruited into the trial individually; the first patient will receive the lowest dose of Imatinib (100mg daily). The highest tolerated dose will be reassessed after each patient completes follow-up and recommended as the dose for the subsequent patient. Dose escalation between patients will be conducted in increments, with no patient receiving more than 100mg than the previous patient (within the 100mg–400mg dose range), guided by the previous dose administered using a statistical model and with oversight from an Independent Data Monitoring Committee. A safety stopping rule has also been imposed if the model identifies with 90% certainty that the lowest dose has a higher toxicity than the target toxicity level (TTL) of 20%. The minimum and maximum number of patients to enter Part 1 of the trial will be 6 and 13, respectively. Under these constraints, using simulation studies we have explored the operating characteristics of this trial design. If our prior toxicities hold true –see Figure 6- the trial design will correctly predict a dose within 10% of the TTL 90% of the time. After the 13$^{th}$ patient completes follow-up (or if a stopping rule is triggered or otherwise stopped early as described in section *Premature Termination of the Trial*) the toxicities and 90% credible intervals will be calculated for each dose level to generate the dose/toxicity quotient based on the data and prior experience from compassionate use. The highest tolerated dose will be recommended as the dose closest to 20% toxicity. We will also report SAE counts and toxicity incidence with or without withdrawal from study.

### Part 2 - Efficacy Phase

Treatment efficacy at the pre-determined highest tolerated dose will be assessed using a Simons two stage design. A null hypothesis that the true response rate is 0.10 will be tested against a one-sided alternative. The first stage interim analysis will happen after 13 patients have completed the 24-week treatment. If there are 1 or no responses# the study will be stopped. If there are 2 or more responses the trial will continue and recruit another 21 patients, creating a maximum sample size of 34 patients. After the final patient is recruited the null hypothesis will be rejected if 7 or more responses are observed. This design ensures a type I error rate of 0.042 and a power of 0.8034 when the true response rate is 0.27. The expected sample size under the null hypothesis is 21 and the probability of an early termination is 0.6213.

Primary analysis of the response rate (PVR reduction after 24 weeks) will be done using an Intention-To-Treat approach. We will provide an alternative Bayesian analysis based on the posterior distribution of the treatment effect of the highest tolerated dose of Imatinib. Treatment effects will be estimated through 95% confidence intervals and credible intervals for the Bayesian analysis using the posterior distribution which results from the data collected and different priors (including a non-informative one). Vague, sceptical and enthusiastic priors will also be considered. The secondary outcomes assessed will be analysed in a similar manner when possible. Secondary analysis will include linear regression models adjusted for potential confounders (e.g., genotype, age, and gender). Continuous data will be summarised with means (geometric means for log-transformed data,

otherwise arithmetic), medians, standard deviations, inter-quartile range, coefficient of variation. Categorical data will be summarised by counts per level within factors. In case of missing data, the missing data mechanism will be explored and multiple imputation applied as a sensitivity analysis if appropriate.

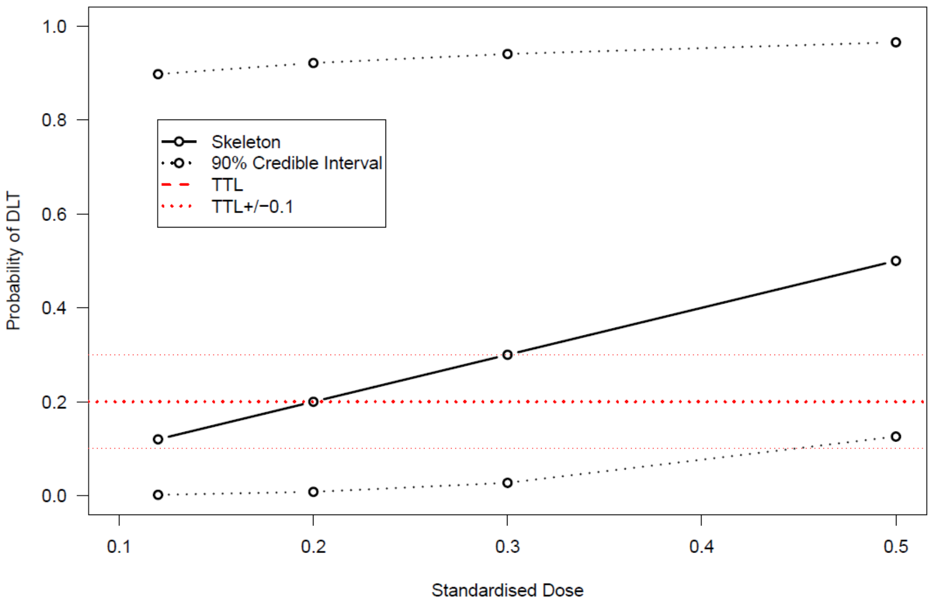


**Figure 6 -** Skeleton

## 11. ADMINISTRATIVE AND LEGAL CONSIDERATIONS

### 11.1 General Legal Requirements

This trial will adhere to the principles outlined in the Medicines for Human Use (Clinical Trials) Regulations 2004 (SI 2004/1031), amended regulations (SI 2006/1928) and the ICH GCP guidelines. It will be conducted in compliance with the protocol, the Data Protection Act and other regulatory requirements as appropriate.

### 11.2 Ethical and MHRA approvals

This study has Clinical Trials Authorisation from the UK Competent Authority; MHRA. The Study Coordination Centre has obtained approval from an NRES Research Ethics Committee (REC), Health Regulator Authority (HRA). The study must receive confirmation of capacity and capability from each participating NHS Trust before accepting participants into the study or any research activity is carried out. The study will be conducted in accordance with the recommendations for physicians involved in research on human subjects adopted by the 18th World Medical Assembly, Helsinki 1964 and later revisions. A list of the REC members must be provided. If any study staff were present, it must be clear that none of these persons voted. Modifications made to the protocol after receipt of the NRES REC/MHRA approvals must also be submitted as amendments by the site Investigator to the NRES REC/MHRA in accordance with local procedures and regulations.

### 11.3 Informed consent

Consent to enter the study must be sought from each participant only after a full explanation has been given, an information leaflet offered and time allowed for consideration. Signed participant consent should be obtained. The right of the participant to refuse to participate without giving reasons must be respected. After the participant has entered the trial the clinician remains free to give alternative treatment to that specified in the protocol at any stage if he/she feels it is in the participant's best interest, but the reasons for doing so should be recorded. In these cases the participants remain within the study for the purposes of follow-up and data analysis. All participants are free to withdraw at any time from the protocol treatment without giving reasons and without prejudicing further treatment.

### 11.4 Patient confidentiality

Participants' identification data will be required for the registration process. The Study Coordination Centre will preserve the confidentiality of participants taking part in the study and is registered under the Data Protection Act.

The site Investigators affirm and uphold the principle of the subject's right to protection against invasion of privacy. Personal health data will be kept confidential.

On eCRFs, paper CRFs or other documents, subjects will be identified by their subject ID only. However, each site Investigator will keep in his/her file a *Subject Identification List*. With respect to the processing of data, every subject has to agree with this in writing. This agreement should be documented together with the written informed consent for trial participation.

### 11.5 Protocol Amendments

Any changes in the protocol will require a formal amendment. Such amendments will be agreed upon and approved in writing by the site Investigator. Amendments to the protocol will be submitted to the relevant authorities and the NRES REC for approval prior to implementation.

Administrative changes which have no significant impact on the medical or scientific validity of the study will be documented in a statement. The NRES REC will be notified of administrative changes, if applicable.

### 11.6 Premature Termination of the Trial

The Chief Investigator reserves the right to terminate the trial for well-documented reasons. Instructions will be provided in a separate document should it be determined that assessments beyond those defined by the protocol are required, also on the recommendations of the independent Trial Steering Committee and Data Monitoring Committee.

Further recruitment of subjects will not take place under the following conditions:
- Premature termination of the trial.
- Drug-related events, i.e. SUSARs, emerging AEs that are serious and the risk/benefit ratio is unacceptable.
- Procedure-related events, i.e., the recruitment rate is too low or the number of dropouts for administrative reasons is too high.
- The model predicts that the lowest dose has a toxicity above the TTL with 90% certainty.

### 11.7 Funder

This project (NIHR128465) is funded by the Efficacy and Mechanism Evaluation (EME) Programme, an MRC and NIHR Partnership. The views expressed in this publication are those of the author(s) and not necessarily those of the MRC, NIHR or the Department of Health and Social Care.

### 11.8 Sponsor

Imperial College London will act as the main Sponsor for this study. Delegated responsibilities will be assigned to the NHS trusts taking part in this study.

### 11.9 Indemnity

Imperial College holds Public Liability ("negligent harm") and Clinical Trial ("non-negligent harm") insurance policies which apply to this trial.

### 11.10 Record Retention

The site Investigator must retain all study records by the applicable regulations in a secure and safe facility. The site Investigator/institution must take measures to prevent accidental or premature destruction of essential documents, that is, documents that individually and collectively permit evaluation of the conduct of a study and the quality of the data produced, including paper copies of study records (e.g. subject charts) as well as any original source documents that are electronic as required by applicable regulatory requirements.

The site Investigator/institution should retain subject identifiers for at least 15 years after the completion or premature termination of the study. Subject files and other source data must be kept for the maximum period of time permitted by the hospital, institution or private practice, but not less than 15 years. These documents should be retained for a longer period, however, if required by the applicable regulatory requirements. It is the responsibility of PI to inform the institution as to when these documents no longer need to be retained.

If a site Investigator moves, withdraws from an investigation, retires, requests to move records to another location or to assign these records to another party or (e.g. other Investigator) who will accept the responsibility, written notice of this transfer must be made to and agreed upon by each party.

### 11.11 Confidentiality

Imperial College London holds negligent harm and non-negligent harm insurance policies which apply to this study/ Imperial College Healthcare NHS Trust holds standard NHS Hospital Indemnity and insurance cover with NHS resolution for NHS Trusts in England, which apply to this study (delete as applicable).

### 11.12 Publications

All publications and presentations relating to the study will be authorised by the Trial Management Group. Members of the Trial Management Group and the Data Monitoring Committee will be listed and contributors will be cited by name if published in a journal where this does not conflict with the journal's policy. Authorship of parallel studies initiated outside of the Trial Management Group will be according to the individuals involved in the project but must acknowledge the contribution of the Trial Management Group and the Study Coordination Centre.

### 11.13 Audits and Inspections

The study may be subject to inspection and audit by Imperial College London under their remit as Sponsor, the Study Coordination Centre and other regulatory bodies to ensure adherence to GCP.
Audits and/or inspections may also be carried out by local authorities, or authorities to which information on this trial has been submitted. All documents pertinent to the trial must be made available for such inspection after an adequate announcement.

### 11.14 Participants' Expenses and Payments

Any reasonable journey costs (costs of fuel, bus or railway tickets) and subsistence costs will be reimbursed to individual patients, provided they are supported by valid receipts. Participants will not receive any other payment for taking part in this clinical study. Travel arrangements can be made as needed through the research site at no cost to the patient. Reasonable travel costs and expenses for going to hospital/clinic for the study visits will be reimbursed ss long as those costs and expenses follow the travel policy for the study site and all necessary receipts to the clinical study team.

## REFERENCES


1. Galiè N, Humbert M, Vachiery JL, Gibbs S, Lang I, Torbicki A, et al. 2015 ESC/ERS Guidelines for the diagnosis and treatment of pulmonary hypertension: The Joint Task Force for the Diagnosis and Treatment of Pulmonary Hypertension of the European Society of Cardiology (ESC) and the European Respiratory Society (ERS): Endorsed by: Association for European Paediatric and Congenital Cardiology (AEPC), International Society for Heart and Lung Transplantation (ISHLT). Eur Heart J. 2016; 37: 67-119.

2. Hoeper MM, Humbert M, Souza R, Idrees M, Kawut SM, Sliwa-Hahnle K, et al. A global view of pulmonary hypertension. Lancet Respir Med. 2016; 4: 306-322.

3. Stacher E, Graham BB, Hunt JM, Gandjeva A, Groshong SD, McLaughlin VV, et al. Modern age pathology of pulmonary arterial hypertension. Am J Respir Crit Care Med. 2012; 186: 261-272.

4. Lau EMT, Giannoulatou E, Celermajer DS, Humbert M. Epidemiology and treatment of pulmonary arterial hypertension. Nat Rev Cardiol. 2017; 14: 603-614.

5. (http://webarchive.nationalarchives.gov.uk/20180307220244/https://digital.nhs.uk/catalogue/PUB30128).

6. Schermuly RT, Ghofrani HA, Wilkins MR, Grimminger F. Mechanisms of disease: pulmonary arterial hypertension. Nat Rev Cardiol. 2011; 8: 443-455.

7. Schermuly RT, Dony E, Ghofrani HA, Pullamsetti S, Savai R, Roth M, et al. Reversal of experimental pulmonary hypertension by PDGF inhibition. J Clin Invest. 2005; 115: 2811-2821.

8. Ghofrani HA, Seeger W, Grimminger F. Imatinib for the treatment of pulmonary arterial hypertension. N Engl J Med. 2005; 353: 1412-1413.

9. Ghofrani HA, Morrell NW, Hoeper MM, Olschewski H, Peacock AJ, Barst RJ, et al. Imatinib in pulmonary arterial hypertension patients with inadequate response to established therapy. Am J Respir Crit Care Med. 2010; 182: 1171-1177.

10. Hoeper MM, Barst RJ, Bourge RC, Feldman J, Frost AE, Galié N, et al. Imatinib mesylate as add-on therapy for pulmonary arterial hypertension: results of the randomized IMPRES study. Circulation. 2013; 127: 1128-1138.

11. Humbert M. Impression, sunset. Circulation. 2013; 127: 1098-1100.

12. Capdeville R, Buchdunger E, Zimmermann J, Matter A. Glivec (STI571, Imatinib), a rationally developed, targeted anticancer drug. Nat Rev Drug Discov. 2002; 1: 493-502.

13. Yu Y, Sweeney M, Zhang S, Platoshyn O, Landsberg J, Rothman A, et al. PDGF stimulates pulmonary vascular smooth muscle cell proliferation by upregulating TRPC6 expression. Am J Physiol Cell Physiol. 2003; 284: C316-330.

14. Montani D, Perros F, Gambaryan N, Girerd B, Dorfmuller P, Price LC, et al. C-kit-positive cells accumulate in remodeled vessels of idiopathic pulmonary arterial hypertension. Am J Respir Crit Care Med. 2011; 184: 116-123.

15. Young KC, Torres E, Hehre D, Wu S, Suguihara C, Hare JM. Antagonism of stem cell factor/c-kit signaling attenuates neonatal chronic hypoxia-induced pulmonary vascular remodeling. Pediatr Res. 2016; 79: 637-646.

16. Cheah CY, Burbury K, Apperley JF, Huguet F, Pitini V, Gardembas M, et al. Patients with myeloid malignancies bearing PDGFRB fusion genes achieve durable long-term remissions with Imatinib. Blood. 2014; 123: 3574-3577.

17. Jawhar M, Naumann N, Schwaab J, Baurmann H, Casper J, Dang TA, et al. Imatinib in myeloid/lymphoid neoplasms with eosinophilia and rearrangement of PDGFRB in chronic or blast phase. Ann Hematol. 2017; 96: 1463-1470.

18. Patterson KC, Weissmann A, Ahmadi T, Farber HW. Imatinib mesylate in the treatment of refractory idiopathic pulmonary arterial hypertension. Ann Intern Med. 2006; 145: 152-153.

19. Souza R, Sitbon O, Parent F, Simonneau G, Humbert M. Long term Imatinib treatment in pulmonary arterial hypertension. Thorax. 2006; 61: 736.

20. Olschewski H. Imatinib for Pulmonary Arterial Hypertension - Wonder Drug or Killer Drug? Respiration. 2015; 89: 513-514.

21. Rhodes CJ, Batai K, Bleda M, Haimel M, Southgate L, Germain M, et al. Genetic determinants of risk in pulmonary arterial hypertension: international genome-wide association studies and meta-analysis. Lancet Respir Med. 2019; 7: 227-238.

22. Rhodes CJ, Wharton J, Ghataorhe P, Watson G, Girerd B, Howard LS, et al. Plasma proteome analysis in patients with pulmonary arterial hypertension: an observational cohort study. Lancet Respir Med. 2017; 5: 717-726.

23. Yorke J, Corris P, Gaine S, Gibbs JS, Kiely DG, Harries C, et al. emPHasis-10: development of a health-related quality of life measure in pulmonary hypertension. Eur Respir J. 2014; 43(4): 1106-13.

24. Mullens W, Sharif F, Dupont M, Rothman AMK, Wijns W. Digital health care solution for proactive heart failure management with the Cordella Heart Failure System: results of the SIRONA first-in-human study. Eur J Heart Fail. 2020. doi: 10.1002/ejhf.1870. Epub ahead of print. PMID: 32476191.

25. Merienne C, Rousset M, Ducint D, Castaing N, Titier K, Molimard M, et al. High throughput routine determination of 17 tyrosine kinase inhibitors by LC-MS/MS. J Pharm Biomed Anal. 2018; 150:112-120.

26. Rich S. Primary pulmonary hypertension: executive summary from the World Symposium on Primary Pulmonary Hypertension. Evian, France: World Health Organization, 199.

27. https://www.accessdata.fda.gov/cdrh_docs/pdf10/p100045d.pdf

## Appendix 1: Schedule of Events - PIPAH study

| STUDY PERIOD | Pretreatment | Dosing | Open Label Treatment Period | | | | | | | | | Follow-up | |
|---|---|---|---|---|---|---|---|---|---|---|---|---|---|
| **Assessment Name** | Assessment 1 (screening) | Assessment 2 (baseline) | Telephone assessment | Telephone assessment | Telephone assessment | Assessment 3 | Assessment 4 | Assessment 5 | Telephone assessment | Telephone assessment | Assessment 6 | Telephone assessment | Unscheduled assessment |
| **Location** | Clinic | Clinic/Home | ☎ | ☎ | ☎ | Clinic/Home | Clinic/Home | Clinic/Home | ☎ | ☎ | Clinic/Home | ☎ | Clinic/Home |
| **Time (weeks)** | Before Week 0 | Week 0 | Week 1 | Week 2 | Week 3 | Week 4 | Week 8 | Week 12 | Week 16 | Week 20 | Week 24 | Week 28 [g] | when needed |
| **Assessment Window (days)** | 0-28 | ± 3 days | ± 3 days | ± 3 days | ± 3 days | ± 3 days | ± 3 days | ± 3 days | ± 3 days | ± 3 days | ± 3 days | ± 3 days | n/a |
| Inclusion/Exclusion criteria | X | – | – | – | – | – | – | – | – | – | – | – | – |
| Written informed consent | X | – | – | – | – | – | – | – | – | – | – | – | – |
| Demographics | X | – | – | – | – | – | – | – | – | – | – | – | – |
| Medical and medication history | X | – | – | – | – | – | – | – | – | – | – | – | – |
| Physical examination | X | X | – | – | – | X | X | X | – | – | X | – | X |
| Concomitant medications | X | X | X | X | X | X | X | X | X | X | X | X | X |
| Vital signs | X | X | – | – | – | X | X | X | – | – | X | – | X |
| WHO Functional Class | X | – | – | – | – | X | X | X | – | – | X | – | X[e] |
| Six-minute walk test (6MWT) | X | X | – | – | – | X | X | X | – | – | X | – | X[e] |
| Borg Dyspnoea Index | X | X | – | – | – | X | X | X | – | – | X | – | X[e] |
| *Right heart catheterisation | – | X[a] | – | – | – | – | – | – | – | – | X[a] | – | – |
| Mouth swab sample | X | – | – | – | – | – | – | – | – | – | – | – | – |
| Haematology blood tests | X | X | – | – | – | X | X | X | – | – | X | – | X[e] |
| Clinical chemistry tests (incl. virology at screening) | X[b] | X | – | – | – | X | X | X | – | – | X | – | X[e] |
| Serum pregnancy test | X[c] | X[c] | – | – | – | X[c] | X[c] | X[c] | – | – | X[c] | – | – |
| Home urine pregnancy test | – | – | – | – | – | – | – | – | X[c,d] | X[c,d] | – | X[c,d] | – |
| Home body weight and ankle swelling self-check | – | – | X | X | X | – | – | – | X | X | – | X | – |
| Imatinib assay | – | X | – | – | – | X | – | – | – | – | X | – | – |
| Research blood samples | – | X | – | – | – | X | – | – | – | – | X | – | – |
| Electrocardiogram (ECG) | – | X | – | – | – | X | X | X | – | – | X | – | X[e] |
| Echocardiogram | X | – | – | – | – | – | – | – | – | – | X | – | X[e] |
| Brain MRI scan | X[a] | – | – | – | – | – | – | – | – | – | – | – | – |
| Optional CT head scan | X[a,f] | – | – | – | – | – | – | – | – | – | – | – | – |
| Quality of Life questionnaire | – | X | – | – | – | X | – | – | – | – | X | – | – |
| **Administration of Imatinib** | – | X | – | – | – | – | – | – | – | – | X | – | – |
| Review of the diary, Imatinib collection and reconciliation | – | – | – | – | – | X | X | X | – | – | X | – | – |
| Report of Adverse Events , if any | – | X | X | X | X | X | X | X | X | X | X | X | X |

*except for patients with an implanted CardioMEMS™ device.

[a]Can be performed on a separate day ± 3 days apart from the original visit date, as needed.

[b]Virology tests apply to this visit only.

[c]For women of childbearing potential.

[d]Urine pregnancy (β-hCG) tests will be performed at home. A telephone call will be performed within 72 hours of the urine test date to enquire about the results.

[e]If clinically required.

[f]A CT head scan will be performed at screening, when MR imaging is contra-indicated or not tolerated. The brain MRI scan will always be the first choice.

[g]The follow-up telephone assessment can be performed after early termination as long as patients are off study drug for 4 weeks (±3 days).

Appendix 2: NCI Common Terminology Criteria for Adverse Events adapted for PIPAH study

Appendix 2 of
PIPAH Protocol - NCI

Appendix 2 of PIPAH Protocol - NCI CTCAE adapted.pdf

Appendix 3

PIPAH
Imatinib in PAH

IMPERIAL

# Positioning Imatinib for Pulmonary Arterial Hypertension (PIPAH Study)

| | |
|---|---|
| **Sponsor:** | Imperial College London, UK |
| **Funding:** | Efficacy and Mechanism Evaluation (EME) Programme, an MRC and NIHR partnership |
| **IRAS registration:** | 274093 |
| **Ethics Reference:** | 20/SC/0240 |
| **MHRA Reference:** | CTA 19174/0419/001-0001 |
| **EudraCT Register:** | 2020-001157-48 |
| **ClinicalTrials.gov:** | NCT04416750 |

## PIPAH Protocol Amendments | Summary of major changes

| Substantial Amendment AM01, 06-OCT-2020 | |
|---|---|
| Section | Changes (in brief) |
| 4.1 Overall study design and plan | Details on set-up for remote monitoring assessments in Sheffield partner site and background to monitoring devices and transfer of relevant data |
| 7.10 Right hearth catheterisation (RHC) | Clarification on when RHC is required and details of indwelling monitoring device |
| **Substantial Amendment AM02, 29-MAR-2021** | |
| Section | Changes (in brief) |
| 6.6 – 6.8 Assessment on Weeks 4, 8, and 12, respectively | Updates on blood sampling for additional pharmacokinetic measurements included |
| **Substantial Amendment AM03, 10-AUG-2021** | |
| Section | Changes (in brief) |
| 4.3.1 Inclusion criteria | Inclusion criterion 2 changes from “PAH which is idiopathic, heritable or associated with anorexigens” to: "PAH which is idiopathic; PAH heritable; PAH associated with connective tissue disease; PAH after ≥1 year repair of congenital systemic to pulmonary shunt; or PAH associated with anorexigens or other drugs". |
| **Substantial Amendment AM04, 19-MAY-2022** | |
| Section | Changes (in brief) |
| 10. Statistics and Data Analysis | Extension with additional patients |
| **Substantial Amendment AM05, 14-NOV-2022** | |
| Section | Changes (in brief) |
| 10. Statistics and Data Analysis | Extension phase - slight increase in the population target |

Appendix 3

PIPAH
Imatinib in PAH

IMPERIAL

# Positioning Imatinib for Pulmonary Arterial Hypertension (PIPAH Study)

| | |
|---|---|
| **Sponsor:** | Imperial College London, UK |
| **Funding:** | Efficacy and Mechanism Evaluation (EME) Programme, an MRC and NIHR partnership |
| **IRAS registration:** | 274093 |
| **Ethics Reference:** | 20/SC/0240 |
| **MHRA Reference:** | CTA 19174/0419/001-0001 |
| **EudraCT Register:** | 2020-001157-48 |
| **ClinicalTrials.gov:** | NCT04416750 |

## PIPAH Protocol Amendments | Summary of major changes

| Substantial Amendment AM01, 06-OCT-2020 | |
|---|---|
| Section | Changes (in brief) |
| 4.1 Overall study design and plan | Details on set-up for remote monitoring assessments in Sheffield partner site and background to monitoring devices and transfer of relevant data |
| 7.10 Right hearth catheterisation (RHC) | Clarification on when RHC is required and details of indwelling monitoring device |
| Substantial Amendment AM02, 29-MAR-2021 | |
| Section | Changes (in brief) |
| 6.6 – 6.8 Assessment on Weeks 4, 8, and 12, respectively | Updates on blood sampling for additional pharmacokinetic measurements included |
| Substantial Amendment AM03, 10-AUG-2021 | |
| Section | Changes (in brief) |
| 4.3.1 Inclusion criteria | Inclusion criterion 2 changes from “PAH which is idiopathic, heritable or associated with anorexigens” to: "PAH which is idiopathic; PAH heritable; PAH associated with connective tissue disease; PAH after ≥1 year repair of congenital systemic to pulmonary shunt; or PAH associated with anorexigens or other drugs". |
| Substantial Amendment AM04, 19-MAY-2022 | |
| Section | Changes (in brief) |
| 10. Statistics and Data Analysis | Extension with additional patients |
| Substantial Amendment AM05, 14-NOV-2022 | |
| Section | Changes (in brief) |
| 10. Statistics and Data Analysis | Extension phase - slight increase in the population target |

# Statistical Analysis Plan

.

| | |
|---|---|
| TRIAL FULL TITLE | Positioning Imatinib for pulmonary arterial hypertension (PIPAH) |
| EUDRACT NUMBER | |
| SAP VERSION | 1.2 (corresponding to PIPAH Part 1) |
| ISRCTN NUMBER | |
| SAP VERSION DATE | 11/06/2024 |
| STATISTICIANS | Martin Law and Sofia Villar (former trial statisticians Mikel McKie and Lauren Bell) |
| TRIAL CHIEF INVESTIGATOR | Martin Wilkins |
| SAP AUTHOR | Sofia Villar, Mikel McKie, Lauren Bell, Martin Law |

## 1 SAP Signatures

I give my approval for the attached SAP entitled PIPAH dated

**Chief Investigator**

Name:

Signature: ____________________________________

Date: 9/6/2024

Name: Sofia S. Villar

Signature: ____________________________________

Date: 9/06/2024

# 2 Administrative Information

a/ SAP Revision History

| SAP Version | Date of SAP version sign off | Justification of SAP Version |
|---|---|---|
| SAP version 1.0 signed off<br>(Mikel Mckie and Sofia Villar) | 26th October 2021 | |
| SAP version 1.1 amendments begin<br>(Lauren Bell and Sofia Villar) | 30th May 2022 | Inclusion of secondary, exploratory outcomes |
| SAP version 1.2<br>(Martin Law and Sofia Villar)<br>Efficacy Analysis Appendix to this SAP written by Alexander Rothman, revised by Martin Law and Sofia Villar | 9th June 2024 | Inclusion of extension cohort and appendix describing exploratory outcome measures and analysis |

# 3 Abbreviations and Definitions

| | |
|---|---|
| AE | Adverse Event |
| CRF | Case Report Form |
| IMP | Investigational Medical Product |
| SAP | Statistical Analysis Plan |
| PAH | pulmonary arterial hypertension |
| CRM | Continual Reassessment Method |
| TNL | target non-tolerability |
| 6MWD | 6 minute walk |
| NTproBNP | N-terminal pro-brain natriuretic peptide |
| SBP | Systolic blood pressure |
| DBP | Diastolic blood pressure |

# 4. Background and Rationale

### 4.1.1 **Overview**

The PIPAH study was designed as a study in two parts. The objective of Part 1 is to identify the best-tolerated dose of imatinib at 4 weeks (between 100 mg and 400 mg once daily for a target treatment duration of at least four weeks) for patients with pulmonary arterial hypertension (PAH) [1]. Part 1 aims to inform the choice of a recommended dose to study in Part Two. The objective of Part 2 is to assess the efficacy of imatinib on pulmonary vascular resistance (PVR) in patients with PAH, administered at the dose recommended from Part 1.

The protocol for both Part 1 and Part 2 is published [1]. We aim to maximise transparency of the planned analysis with the intention of eradicating misreporting or selective reporting of the trial data. Following 'Guidance of the Content of Statistical Analysis Plans in Clinical Trials' [2] and 'Early phase clinical trials extension to guidelines of the content of statistical analysis plans' [3], this statistical analysis plan (SAP) details the planned analysis of the aims and objectives of Part 1.

Part 1 is a dose-escalation trial, undertaken through the Continual Reassessment Method (CRM) [4] and this SAP describes the escalation framework and methods of estimation and inference for the tolerability estimates of doses in the 100-400mg range. In addition to the primary objective, we report the objectives, outcomes, and methods for estimation and reporting of data connected to two important secondary/exploratory objectives: (i) signals of efficacy of doses allocated within the 100-400mg range, and (ii) longer-term tolerability, measured over an 8-week period.

#### 4.1.2 **Background and main rationale**

There is a growing evidence-base demonstrating the promise of imatinib for treating patients with pulmonary arterial hypertension (PAH). Imatinib inhibits platelet-derived growth factor receptor (PDGFR) alpha and beta and is thought to have antiproliferative properties that might halt or reverse pulmonary hypertension. That is, imatinib is expected to reduce total pulmonary resistance in patients with PAH, which leads to an improvement in the health and quality of life of patients. However, pursuits to find an efficacious dose have been challenged by patients discontinuing treatment because they could not tolerate a daily dose over time. An urgent goal is to identify an efficacious daily dose of imatinib, in the range 100mg to 400mg, which is tolerable for patients with PAH.

Part 1 of the study is motivated by safety concerns that arose from a phase 3 trial (IMPRES) [5]. IMPRES was a randomised, double blinded trial to determine if a 400mg dose of imatinib, administered daily, improved functional capacity and haemodynamics in patients with PAH. The study reported a significant increase in 6-minute walk distance, a measure of function and the primary prespecified outcome; however, only 44% of patients were able to tolerate the drug at a dose of 400 mg beyond 11 weeks. Due to the high levels of discontinuation of imatinib, the FDA requested further safety data and imatinib, despite its potential as an efficacious treatment for PAH, remains currently unlicensed for this condition.

The design of Part 1 of PIPAH uses a Bayesian CRM, incorporating both the information from IMPRES and expert opinion for determining tolerability of imatinib at 4 weeks, to explore doses in the 100-

400mg range. Patients are recruited sequentially to the study. Outcomes on tolerability from previous recruited patients at the 4-week follow-up timepoint are used to update the Bayesian model and estimate the best tolerated dose for the next patient enrolled into the trial.

The primary objective of Part 1 is to find the best tolerated dose with a pre-specified target non-tolerability (TNL) level of 20%, within the accuracy range of +/- 10%. That is, we set the recommended dose to have an estimated risk of a patient discontinuing imatinib for 5 or more consecutive days at approximately 20%, and within a 10-30% range. Using simulation studies, we determined that a sample size of 13 patients should correctly identify the best tolerated dose 90% of the time.

We aim to make use of data gathered during to the Part 1 study, to explore signals of efficacy as well as longer term tolerability. At the start of the trial we expected to recruit a small number of patients to PIPAH Part 1 who have FDA/CE-approved pulmonary artery pressure monitors (CardioMEMS) and activity monitors which capture and relay daily haemodynamic measures. These data present the opportunity to better understand any signals of efficacy and thus make a better decision on a dose carry forward to part 2 of the study. At the end of the study, the number of patients with a CardioMEMS was higher than originally anticipated (13 out of 17). This explains why the dosing design did not consider using the efficacy data to guide dosing decisions in the original CRM Model (as discussed in [7]). Future work could include a redesign of PIPAH part 1 that considers how dosing decisions could have differed if both efficacy and tolerability signals are used to inform dosing. The final analysis performed to explore efficacy data are described in an appendix to this SAP.

Part 2 of PIPAH will have a separate analysis plan. This SAP documents the rationale and reporting that will be followed for data collected in Part 1.

### 4.1.3 **Justification of the additional, explorative, secondary outcomes**

*Long-term tolerability*

CRM dose finding trials involve the sequential recruitment of patients. This framework sees patients enrolled in a sequential manner, where (i) one patient (or a cohort or *batch* of patients) is allocated to the trial, (ii) follow-up of all outcomes are observed for this patient and (iii) the posterior outcomes are then updated to inform what dose the next patient is to receive. This sequential element to the trial requires the current patient (or cohort of patients) to complete their primary follow-up measure before the next patient is enrolled into the trial. When individuals are recruited sequentially, the duration of the whole trial can, on average, be expected to be roughly no less than the number of patients

multiplied by the follow-up period. For PIPAH Part 1, the follow-up period for the primary tolerability outcome measure was set to 4 weeks, a pragmatic decision to ensure the feasibility of delivering the trial in an acceptable time and cost.

The recommended dose will be that recommended by the CRM at the end of Part 1 i.e., when patient 13$^{th}$ has the primary outcome of tolerability measured at 4 week. We propose to fit a secondary model with this follow-up period to explore longer term tolerability at the 8-week mark with the data of longer term follow up on tolerability. We will fit a full CRM for the 8-week mark, as we plan to have complete measures for all patients at the time of final analysis.

4.1.4 *Efficacy signals and extension cohort*

In early-stage trials, we are often able to observe a response for efficacy, such as a haemodynamic biomarker. In the context of PIPAH, a reduction in total pulmonary pressure over time is considered a signal that an early targeted therapeutic pathway is reached. Thus, responses for early efficacy can be a monotonic relationship with dose, such that higher doses yield, on average, better clinical outcomes. Alternatively, the relationship of total pulmonary pressure and dose may not be directly linear, where early targeted therapeutic pathways are reached through a range of doses over time. The efficacy-dose curve may have a steep slope at lower doses, reach a maximum efficacy ceiling at a certain dose and then plateau at higher doses. In such circumstances, the goal is to understand if a range of doses have an equivalent probability of reaching this targeted therapeutic signal of total pulmonary pressure, and then understand the dynamics and associations of such dosing levels with both short-term and longer-term tolerability events over time.

To better explore the above, an extension cohort of 4 patients at doses at or below the maximum tolerated dose from the CRM at patient 13 was recruited. This would provide additional data on both efficacy signal and tolerability of lower doses to complement the CRM dose recommendation based solely on the 4 week tolerability data.

In the context of PIPAH Part 1 trial, exploring the nature of efficacy signals may add light to the overall aim to understand how different doses of imatinib impacts a patients total pulmonary resistance over time. We will explore the continuously gathered efficacy outcomes from the FDA/CE-approved pulmonary artery pressure monitors (CardioMEMS) to compliment the primary objective of the best tolerated dose. This analysis is described in the appendix to this SAP.

## 4.2 Study Objectives

**Primary Objective (Part 1):** To identify the best tolerated dose of imatinib (as the discrete range of 100mg, 200mg, 300mg or 400mg) daily for 4 weeks. The best tolerated dose is defined to have a target non-tolerability level (TNL) of 20%. This is the risk a patient has, on average, of not tolerating imatinib which results in a discontinuation of treatment for 5 or more consecutive days at any stage during the first 4 weeks of treatment.

### Secondary, Explorative Objectives

1. **Signals of efficacy:** to explore the efficacy-tolerability relationship at the 8-week mark. Variables reported are described in the SAP appendix.

2. **Signals of longer-term intolerability:** to estimate the events of longer-term intolerability, over an 8-week exposure period.

3. **Secondary variables**: PAH classification, WHO Functional Class, 6MWD, Borg Dyspnoea Index, NTproBNP, SBP, DBP, Treatment (before IMP).

### 4.3 Inclusion-Exclusion Criteria and General Study Population

Inclusion Criteria

1. Subjects aged between 18-75 years old
2. PAH which is idiopathic, heritable or associated with anorexigens
3. Resting mean pulmonary artery pressure >25 mmHg, Pulmonary capillary wedge pressure ≤15 mmHg, PVR >5 wood units, and normal or reduced cardiac output, as measured by right heart catheterisation (RHC) at entry
4. Six-minute walking distance >50m at entry
5. Stable on an unchanged PAH therapeutic regime comprising at least 2 therapies licensed for PAH (any combination of endothelin receptor antagonist, phosphodiesterase inhibitor or prostacyclin analogue) for at least 1 month prior to screening
6. Able to provide written informed consent prior to any study mandated procedures. Women of child-bearing potential are eligible to participate, if they agree to use one of the following contraception methods: Abstinence OR Highly effective contraceptive methods with typical-use failure rate <1% i.e.

o Male or female sterilisation and long-acting reversible contraceptive methods (intrauterine devices and implants) prior to the female subject's entry into the study

o Progestogen-only injections if repeat rejections are documented as having been administered on schedule by a healthcare professional.

Exclusion Criteria

1. Unable to provide informed consent and/or are non-fluent speakers of the English language
2. Hypersensitivity to imatinib or to any of the excipients
3. Clinically-significant renal disease (confirmed by creatinine clearance <30 ml/min per 1.73m2)
4. Clinically-significant liver disease (confirmed by serum transaminases >3 times than upper normal limit)
5. Patients receiving oral and/or parenteral anticoagulants
6. Anaemia confirmed by haemoglobin concentration <10 g/dl
7. History of thrombocytopenia
8. Individuals known to have haemoglobinopathy sickle cell disease, thalassaemia
9. Hospital admission related to PAH or change in PAH therapy within 3 months prior to screening
10. History of left-sided heart disease and/or clinically significant cardiac disease, including but not limited to any of the following:
    - Aortic or mitral valve disease (stenosis or regurgitation) defined as greater than mild aortic insufficiency, mild aortic stenosis, mild mitral stenosis, moderate mitral regurgitation
    - Mechanical or bioprosthetic cardiac valve
    - Pericardial constriction/effusion with tamponade physiology, or abnormal left atrial size.
    - Restrictive or congestive cardiomyopathy
    - Left ventricular ejection fraction ≤50% (measured in echocardiogram at screening)
    - Symptomatic coronary disease
    - Significant (2+ for regurgitation) valvular disease other than tricuspid or pulmonary
    - regurgitation
    - Acutely decompensated left heart failure within 1 month of screening
    - History of untreated obstructive sleep apnoea
11. Evidence of significant lung disease on high-resolution CT (if available) or recent (performed within 12 months) lung function, where FEV1 <50% predicted and FVC <70% predicted, and DLCO (or TLCO) <50% predicted if any CT abnormalities; judged by the Site Physician
12. Patients with a history of uncontrolled systemic hypertension
13. Acute infection (including eye, dental, and skin infections)

14. Chronic inflammatory disease including HIV and Hepatitis B
15. Women of childbearing potential who are pregnant or breastfeeding
16. Previous intracerebral haemorrhage
17. Patients who have received an IMP within 1 month before the baseline visit

6.3 Randomisation and Blinding: This is an open label non-randomised safety and efficacy trial.

### 4.4 Trial Design

The CRM will explore four doses: 100mg, 200mg, 300mg and 400mg. The trial design is a Continual Reassessment Method [5]. This method adopts a Bayesian approach, by combining prior information gained through the IMPRESS trial with the evolving, sequential data coming in through the PIPAH trial. Patients enter the trial at the specified dose that is based upon the previous patient's outcomes and the prior distributions.

After a patient's outcomes are observed, the posterior distribution is calculated to estimate the probability of non-tolerability at each dose. The next patient is then entered into the trial and assigned the dose closest to the desired level of tolerability based on the updated posterior means. The dose-escalation, decision-making framework of which dose to test sequentially during the trial is detailed below. Methods of estimation for the primary and secondary outcomes are detailed in following next section

### 4.5 Patient Characteristics

Descriptive statistics (frequency distributions and measures of central tendency) will be used to characterise the baseline and demographic variables of the patients recruited to PIPAH.

### Baseline

We will tabulate the reported baseline measures of: Sex, age (years), body mass index, blood pressure – diastolic (mm Hg), blood pressure – systolic (mm Hg), total pulmonary resistance (dynes), mean pulmonary artery pressure (mm Hg), cardiac output, cardiac index, functional class (expressed as a proportion of either 1,2,3,4), Log2 NT-proBNP, PH treatment naïve, ERA, prostanoid, PDE5, other PH drug.

### 4.6 Sample Size

Part 1 will recruit a sample size between 6 to 13 patients. Simulation studies reported in section show that if our prior tolerability expectations are true, with a sample size up to 13, we will correctly identify the best tolerated dose 90% of the time. See section 5 for simulation results.

### 4.6.1 Dose finding and safety analyses

Part 1 of this study is solely concerned determining the highest tolerable or safe dose (MTD) of imatinib. This is being done within a Bayesian continual reassessment method (CRM) framework. Determination of the model parameters was carried out by simulation to optimise the outcomes based on our prior understanding or toxicity. Please see below for details on the prior toxicities, specific model parameters and the derived skeleton dose-toxicity curve.

### 4.6.2 Prior guess of toxicities

Imatinib is a widely used chemotherapeutic drug so there is quite a lot of clinical understanding of the dose toxicity relationship in cancer patients. Furthermore, a randomised clinical trial has already been carried out to attempt to position imatinib to treat patients with PAH (REF). This study was unsuccessful largely due to patient drop out from drug toxicity at the study dosage. We have generated our prior understanding of the dose-toxicity relationship of imatinib by combining information from the drop-out rates of the RCT and clinical understanding from using imatinib in PAH on compassionate grounds and for cancer treatment. Our predicted toxicity likelihood at each dose is outlined below:

*Table 2: Toxicity predictions*

| Dose | 100 mg | **200 mg*** | 300 mg | 400 mg |
|---|---|---|---|---|
| Prior guess of risk | 0.12 | 0.20 | 0.30 | 0.50 |

* Prior prediction of MTD

### 4.6.3 Model parameters and stopping rules

The target toxicity level for the CRM was determined to be dose closest to 20% toxicity, chosen to optimise number of patients on the drug whilst maintaining a good chance of find any efficacy in part 2. The base dose toxicity relationship was determined to be a simple single parameter power model in the form, p(tox|d) = $d^{\alpha}$, where our single parameter α follows a gamma(1,1) distribution. These parameters combined with our prior information results in the following dose toxicity skeleton.

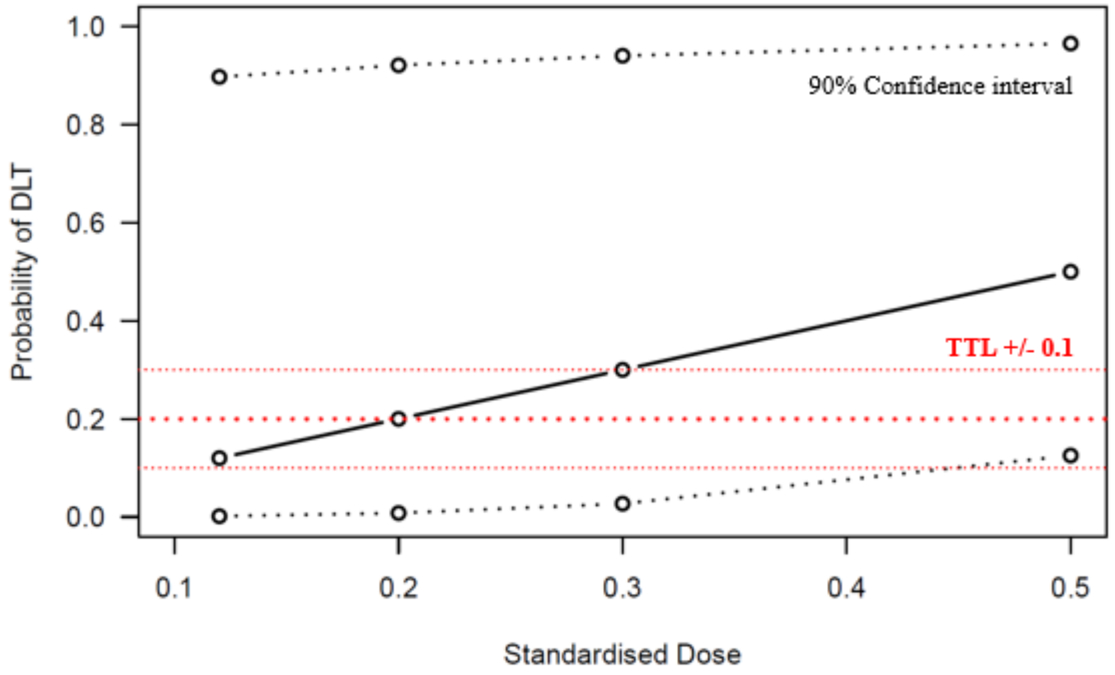


We also built in study specific constraints and stopping rules that will alter the behaviour of our CRM. These include:

- The first patient will be started on the lowest dose
- Patients are recruited individually
- Dose escalation or de-escalation are limited to single dose level
- The trial will stop early if it is determined with 90% probability that all doses are above the MTD
- There is a minimum sample size of 6 and a maximum of 13
- 

#### 4.6.4 Determination of a toxic response

A toxic outcome is determined if the current patient discontinues taking the study drug due to intolerable side effects for 5 consecutive days in the 4-week treatment period.

## 5 Study operating characteristics

To test how well these study parameters performed we simulated different trial conditions and assessed the resulting operating characteristics. We used the following different scenarios:

(a) Toxicities are as expected

(b) Toxicity is increased by 20%

(c) Toxicity is decreased by 20%

(d) The rate change of toxicity between doses is doubled

(e) The rate change of toxicity between doses is halved

(f) All doses are above the target toxicity (but increase at same rate)

*Table 3: Toxicities in different scenarios*

| Dose | 100 mg | 200 mg | 300 mg | 400 mg |
|---|---|---|---|---|
| (a) | 0.120 | **0.200** | 0.300 | 0.500 |
| (b) | **0.144** | **0.240** | 0.360 | 0.600 |
| (c) | 0.096 | **0.160** | **0.240** | 0.400 |
| (d) | **0.120** | **0.280** | 0.480 | 0.880 |
| (e) | 0.120 | 0.160 | **0.210** | 0.310 |
| (f) | 0.330 | 0.370 | 0.420 | 0.520 |

*Bolded is the MTD for each scenario

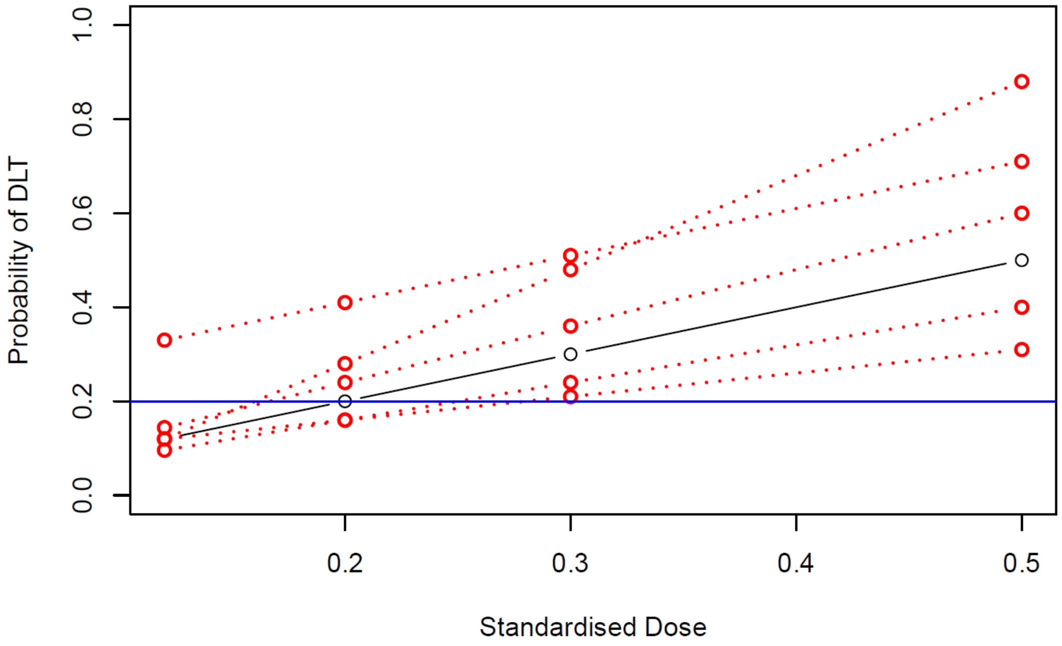


Red – skeletons for all scenarios, Black – Predicted skeleton, Blue – Target toxicity level (TTL)

Each scenario was simulated 1000 times to generate useful average characteristics such as the proportion of trials correctly determining the MTD and the average number of toxicities. As our target precision is +/- 10% we created an acceptable dosing interval of 10-30% chance of dose limiting toxicity (DLT) and aggregated the simulation data to show the probability of recommending a dose within our acceptable range rather than the true MTD.

*Table 4: Results of simulation study*

| | CRM - Probability of DLT (%) | | | |
|---|---|---|---|---|
| Scenario | 0-10 | 10-30 | 30-60 | 60+ |
| | Experimental proportions | | | |
| (a) | 0 | 0.915 | 0.0847 | 0 |
| (b) | 0 | 0.702 | 0.298 | 0 |
| (c) | 0.27 | 0.583 | 0.147 | 0 |
| (d) | 0 | 0.801 | 0.176 | 0.0234 |
| (e) | 0 | 0.805 | 0.195 | 0 |
| (f) | 0 | 0 | 0.975 | 0.0246 |
| | Recommended proportions | | | |
| (a) | 0.06 | **0.898** | 0.042 | 0 |
| (b) | 0.082 | **0.687** | 0.231 | 0 |
| (c) | 0.171 | **0.693** | 0.136 | 0 |
| (d) | 0.064 | **0.816** | 0.117 | 0.003 |
| (e) | 0.035 | **0.75** | 0.215 | 0 |
| (f) | **0.555** | 0 | 0.444 | 0.001 |

*Bolded is the proportion of trials that recommended an acceptable dose.

The results showed that if the toxicities are as expected the design will have a 90% of picking an acceptable dose. This is the basis for our original sample size calculation. Furthermore, in all our scenarios that deviate from our prior predictions the most likely outcome is always an acceptable dose.

*Table 5: Average sample sizes of CRM*

| | CRM - Sample size | | |
|---|---|---|---|
| Scenario | Mean | Minimum | Maximum |
| (a) | 12.665 | 6 | 13 |
| (b) | 12.58 | 6 | 13 |
| (c) | 12.909 | 6 | 13 |
| (d) | 12.663 | 6 | 13 |
| (e) | 12.788 | 6 | 13 |
| (f) | 9.904 | 6 | 13 |

As there are early stopping rules, we check what the average sample sizes were for our simulated trials and found in all scenarios nearly all cases used the maximum sample size with only scenario f (the most extreme toxicities) having a smaller on average sample size due to the safety stopping rule. As expected, these sample sizes are roughly in line or smaller than an equivalent 3+3 design. Taken all together our CRM design has favourable operating characteristics.

Additional considerations:

The one-parameter model is commonly implemented for the CRM design. However, a trade-off with the one-parameter model is that, in pursuit of accurately determining the estimated risk of tolerability of the dose closest to the TNL as efficiently as possible, exploration rates of lower doses is reduced. An alternative two-parameter model may direct more exploration to the lower doses in such circumstances, or further evaluation of lower doses can be achieved through a cohort extension that would depart from the recommendations of the one-parameter model. The latter is the approach that was implemented in PIPAH part 1.

## 6 Reporting Adverse Events

All AEs will be reported. Depending on the nature of the event the reporting procedures set out in study protocol should be followed. Any questions concerning AE reporting will be directed to the study coordination centre in the first instance. Summary statistics will be produced in accordance with Sections 12 and 13 and an example can be shown in the report template of section 15.

## 7 Statistical interim analysis and stopping guidance

A safety stopping rule is in place. The study will stop when the model identifies a dose, within a +/-10% range, that has a non-tolerability target level of 20%. We included a lower bound of six patients to ensure that any intolerable responses in the first patients do not trigger an early termination of the trial.

## 8 Methods of Estimation and Inference

The findings will report at each interim analysis the estimated non-tolerability probability for each of the four doses, with 90% credible intervals. The credible intervals will be narrower for doses which are closer to the targeted non-tolerability level of 20% and therefore sampled more.

## 9 Timing of final analysis and Withdrawal and Follow-Up

With 8-week outcome data, we will conduct the analysis of longer-term tolerability will fully observed outcomes for the whole trial.

## 10. Measures

Each patient will have recorded one response of tolerability at 4-weeks and one at 8-weeks and one continuous efficacy response (defined and analysed as per SAP appendix). The tolerability response is equal to one if intolerance occurs in the first four weeks of treatment and zero otherwise.

### 10.1 Outcome definitions

*Primary Outcome*

The primary outcome is the binary (yes/no) tolerability. A patient does not tolerate Imatinib if treatment is discontinued for five or more consecutive days during the first four weeks of treatment. The reasons for discontinuation of Imatinib must be due to Grade 2 or above Adverse Events defined by the National Cancer Institute (NCI) Common Terminology Criteria for Adverse Events (version 5.0, 2017), adapted for this trial.

### 10.2 Adherence to treatment policy

The primary outcome will be analysed at the 4-week mark from the start of treatment policy. For example, if a patient tolerates the dose at 4-weeks but does not tolerate the dose at 6-weeks and changes dose, their outcomes will be used for analysis regarding the 4-week time point as a well-tolerated dose, but as a none tolerability event for the 8-week timepoint.

### *10.3 Secondary Outcomes*

The secondary outcomes include:

1. ***Signal of efficacy:*** see measures described in appendix to this SAP.

2. ***Signal of longer-term tolerability:*** The event (occurrence and timing) if a patient experiences intolerability, defined as the discontinuation of Imatinib for five or more consecutive days during the first 8-weeks. The timestamp of intolerability will be recorded at the 5$^{th}$ day of discontinuation.

## 11 General Considerations

### 11.1 Timing of Analyses

The final analysis for part 1 will take place after the 13th patient has either completes the 4-week treatment of Imatinib or has stopped taking imatinib for 5 consecutive days.

### 11.2 Covariates and Subgroups

The only subgroup analyses that may be considered will be the patients genotype at the rs3816018 locus. We assess the effect of Imatinib on change in PVR according to genotype as a secondary analysis. Reporting this subgroup is exploratory and will only be done in case sufficient data on this genotype is available at time of reporting.

### 11.3 Missing Data

Missing data in the primary outcome is not expected. Missing data in secondary endpoints will be quantified per variable (total and %).

### 11.4 Interim Analyses and Data Monitoring

#### 11.4.1 Purpose of Interim Analyses

During part 1 an interim analysis will take place either after each patient has either completed 4 weeks of treatment with Imatinib at a given dose or after a patient has not taken their dose of Imatinib for 5 consecutive days due to toxicity. This will allow us to update the dose toxicity curve so that the next recommended dose is as accurate as possible. The analyses will also allow for the study to be stopped early by triggering a safety stopping rule.

#### 11.4.2 Planned Schedule of Interim Analyses

The interim analysis for part 1 will happen either after each patient has either completed 4 weeks of treatment with Imatinib at a given dose or after a patient has not taken their dose of Imatinib for 5 consecutive days due to toxicity. A sample report that will be used for reporting to the study IDMC is included in section 15.

#### 11.4.3 Scope of Adaptations

The CRM for part 1 will recommend the dose that is predicted to be the MTD. This means the adaptation may be a dose escalation, de-escalation or continuation of dose. In the case the predicted MTD is more than one dose level from current dose the dose recommendation will be limited to a single dose step

to avoid extreme jumps in dose. The final recommended dose will the dose predicted to be the MTD after patient 13 at 4 week follow up.

### 11.5 Adjustment of Credible intervals, confidence Intervals and p-values

After completion of part 1, the dose toxicity curve will be calculated with 90% credible intervals as a measure of uncertainty in the model. No further adjustments are planned.

#### 11.5.1 Interim Analysis for Sample Size Adjustment

In part 1 the minimum sample size is 6 and the maximum is 13 participants. The sample size may be cut if an early stopping rule is triggered.

### 11.6 Documentation of Interim Analyses

The data available at each interim will be saved as well as any code used for calculations and reports made to the IDMC and TSC. The versions of any documentation used for each interim will also be saved to account for any amendments. An interim report template can be found in section 15 of this SAP.

### 11.7 Multi-centre Studies

This is a multi-centre study but no explicit adjustments for interactions or bias have been considered.

### 11.8 Multiple Testing

Operating characteristics have been controlled for the primary endpoint only (that is for the CRM model) and no adjustment for multiple comparisons in the secondary endpoints will be carried out so these results should be considered exploratory.

## 12 Summary of Study Data

All continuous variables will be summarised using the following descriptive statistics: n (non-missing sample size), mean (geometric if log-transformed otherwise arithmetic), and standard deviation, median, maximum and minimum. The frequency and percentages (based on the non-missing sample size) of observed levels will be reported for all categorical measures. In general, all data will be listed, sorted by site, dose and subject, and when appropriate by visit number within subject. All summary tables will be structured with a column for each dose and will be annotated with the total population size relevant to that table/treatment, including any missing observations.

## 13 Reporting Conventions

P-values ≥0.001 will be reported to 3 decimal places; p-values less than 0.001 will be reported as "<0.001". The mean, standard deviation, and any other statistics other than quantiles, will be reported to one decimal place greater than the original data. Quantiles, such as median, or minimum and maximum will use the same number of decimal places as the original data. Estimated parameters, not on the same scale as raw observations (e.g. regression coefficients) will be reported to 3 significant figures.

## 14. Statistical Software

The following R packages will be used for each analysis.

| Analysis | R package |
|---|---|
| **Primary Analysis:** Best tolerated dose at week 4 | bcrm [8] |
| **Secondary Analysis:** Best tolerated  at week 8 | bcrm [8] |

## 15. Reporting Template (for Continuous Reassessment Method)

PIPAH Imatinib in PAH

### TRIAL STATISTICIANS' INTERIM REPORT

**Chief Investigator:** Prof Martin R Wilkins (Imperial College London)
**Trial Statisticians:** Dr Sofía Villar & Dr Mikel McKie
(Papworth CTU & MRC Biostatistics Unit, Cambridge)

| REPORT IDENTIFICATION | | | |
|---|---|---|---|
| Report ID: | PIP-007 | Date of Report: | dd/mm/yr |

*The validity of the actual clinical research data presented below is the responsibility of the reporting Site.*

| INDIVIDUALS – SUMMARY | | | |
|---|---|---|---|
| Subject ID: | 44300XX | Research Site: | 4430 |
| Subject Allocated Dose: | | 300mg | |
| Compliance % rate to date (if known): | | 100% | |
| Treatment duration to date (weeks): | | 4 weeks | |
| Treatment Tolerability during study period: | | Tolerated | |
| Subject AEs | | | |

| AE NUMBER | 1 | 2 |
|---|---|---|
| **SUBJECT** | 44300XX | 44300XX |
| **CTCAE TERM** | Bloating | Periorbital oedema |
| **ONSET DATE OF AE** | 12/10/20XX 00:00 | 18/10/20XX 00:00 |
| **CTCAE SEVERITY** | Grade 2 | Grade 1 |
| **STUDY DRUG DOSE** | 300mg | 300mg |
| **CAUSALITY/RELATIONSHIP** | Possibly | Probably |
| **ACTION TAKEN** | None | None |
| **NOTES** | NA | NA |

| OVERALL – SUMMARY | | | | | | | | | | |
|---|---|---|---|---|---|---|---|---|---|---|
| Treated Subjects to date: | 7 | | | | | | | | | |
| Reported AEs to date: | 16 | | | | | | | | | |
| CTCAE Grades (all subjects): | Grade 1: | 10 | Grade 2: | 6 | Grade 3: | 0 | Grade 4: | 0 | Grade 5: | 0 |

Table 1: Posterior estimates of dose toxicity

| Posterior estimates of Toxicities | Dose 100 | 200 | 300 | 400 |
|---|---|---|---|---|
| Mean | 0.062101 | 0.10756 | 0.173566 | 0.339353 |
| SD | 0.078016 | 0.103578 | 0.128406 | 0.155218 |
| Median | 0.033793 | 0.076431 | 0.146085 | 0.330407 |
| Plug-in Estimate* | 0.02442 | 0.059729 | 0.121478** | 0.297119 |

* Plug-in estimate denotes the toxicity estimate using the mean posterior estimation of the model parameter plugged into the toxicity model. This is the metric used to decide the next recommended dose.
** Current estimation of HTD

Table 2: Credible intervals of posterior estimates of dose toxicity

| Quantile | Dose 100 | 200 | 300 | 400 |
|---|---|---|---|---|
| 2.50% | 0.000324 | 0.002248 | 0.010445 | 0.072351 |
| 10% | 0.002273 | 0.00985 | 0.031547 | 0.136718 |
| 25% | 0.01064 | 0.03179 | 0.07579 | 0.226451 |
| 50% | 0.033793 | 0.076431 | 0.146085 | 0.330407 |
| 75% | 0.082657 | 0.150707 | 0.242765 | 0.44263 |
| 97.50% | 0.292767 | 0.393596 | 0.497817 | 0.669264 |

Figure 1: Current estimation of the Dose toxicity curve.

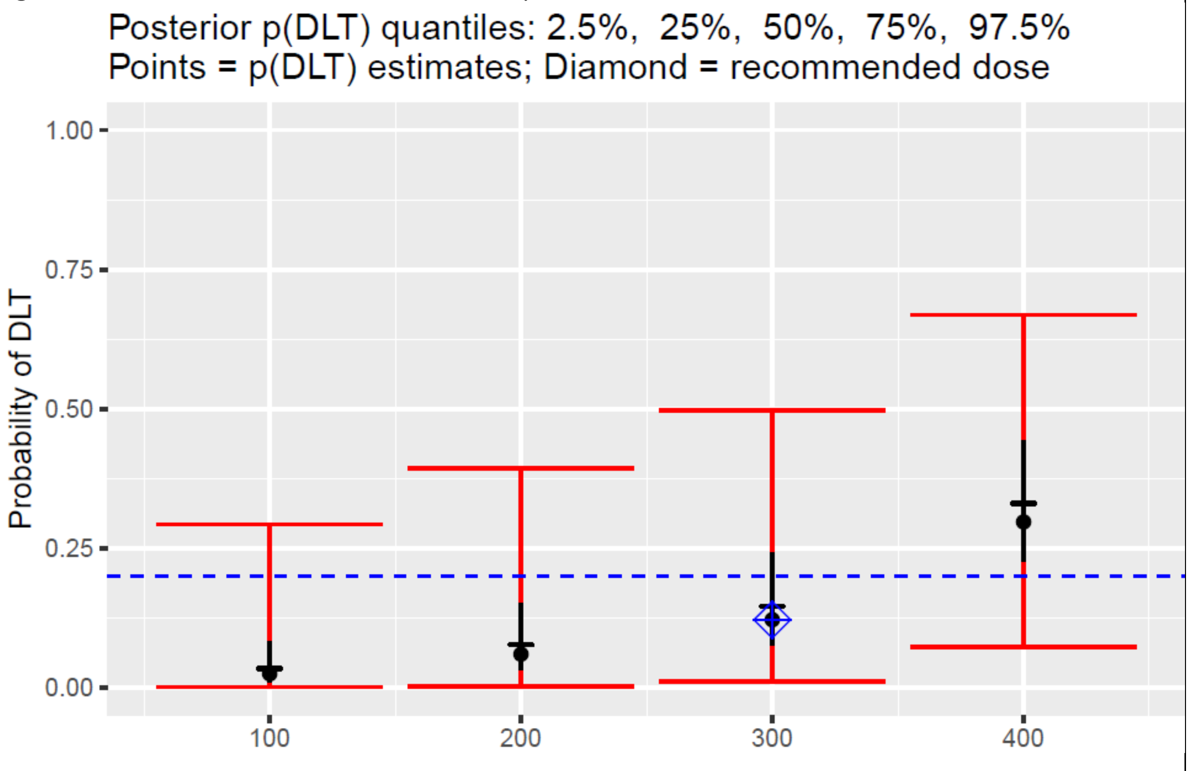

Figure 2: Simplified dose/toxicity showing our prior guess and updated toxicities.

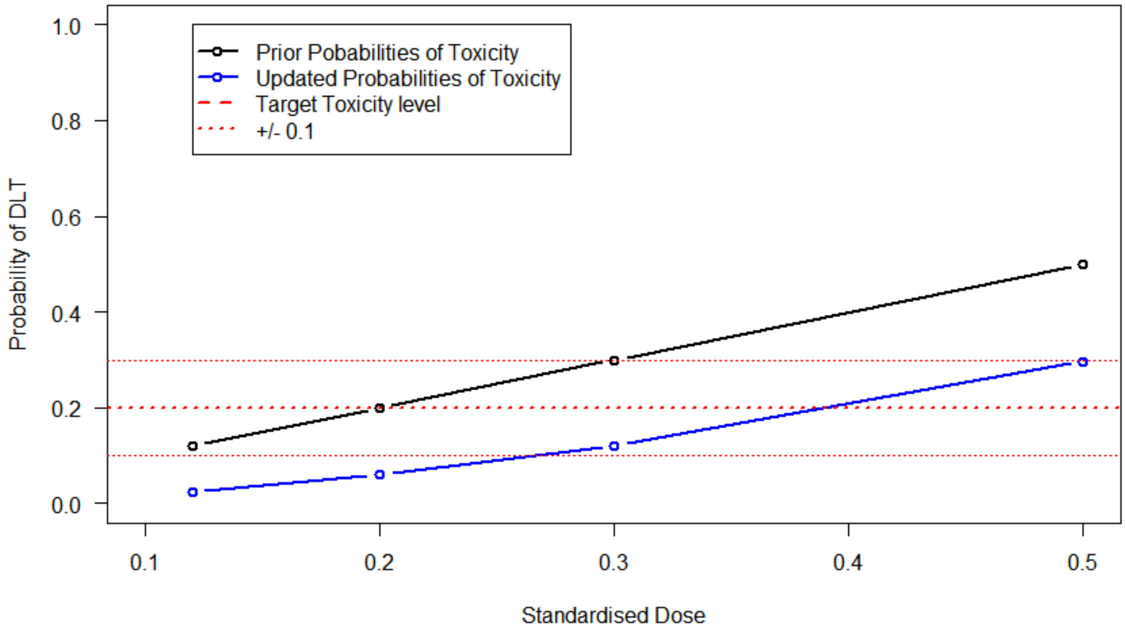


| Prior highest tolerated dose (HTD) estimation: 300mg | Current HTD estimation: 300mg |
|---|---|

| OUTCOME: | |
|---|---|
| Subsequent subject | |
| XXXXXXX | The current best estimation of the HTD is 300mg. We recommend the next patient is remains on 300mg.<br>Confirmation needed by independent DMC and TSC. |

## APPROVAL

To my knowledge, the content of this report is correct:

Signature: Sofia S. Villar

Date:

*This report can be signed electronically to avoid delay.*

## References


1. Wilkins, M.R., et al., *Positioning imatinib for pulmonary arterial hypertension: A phase I/II design comprising dose finding and single-arm efficacy.* Pulmonary Circulation, 2021. **11**(4): p. 20458940211052823.
2. Gamble, C., et al., *Guidelines for the Content of Statistical Analysis Plans in Clinical Trials.* JAMA, 2017. **318**(23): p. 2337-2343.
3. Homer, V., et al., *Early phase clinical trials extension to guidelines for the content of statistical analysis plans.* BMJ, 2022. **376**: p. e068177.
4. O'Quigley, J., M. Pepe, and L. Fisher, *Continual reassessment method: a practical design for phase 1 clinical trials in cancer.* Biometrics, 1990. **46**(1): p. 33-48.
5. Hoeper, M.M., et al., *Imatinib mesylate as add-on therapy for pulmonary arterial hypertension: results of the randomized IMPRES study.* Circulation, 2013. **127**(10): p. 1128-38.
6. Cheung, Y.K. and R. Chappell, *Sequential designs for phase I clinical trials with late-onset toxicities.* Biometrics, 2000. **56**(4): p. 1177-82.
7. Braun, T.M., *The bivariate continual reassessment method. extending the CRM to phase I trials of two competing outcomes.* Control Clin Trials, 2002. **23**(3): p. 240-56.
8. Sweeting, M., A. Mander, and T. Sabin, *bcrm: Bayesian Continual Reassessment Method Designs for Phase I Dose-Finding Trials.* Journal of Statistical Software, 2013. **54**(13): p. 1 - 26.

## SAP Appendix

**Statistical Analysis Plan for Efficacy Analysis**

**Endpoint efficacy measures**.

Key clinical endpoints in PAH studies comprise changes in cardiopulmonary haemodynamics, notably pulmonary artery pressure (PAP), cardiac output (CO), and heart rate, along with functional measures such as change in WHO functional class and exercise activity (e.g. 6 minute walk distance) and biochemical markers, such the change in plasma NTproBNP.

The change in cardiopulmonary haemodynamics is usually captured by repeat right heart catheterisation – at baseline and end of study. A reduction in resting mean PAP (mPAP) coupled with an increase in CO, signals a reduction in pulmonary vascular resistance (PVR = mPAP-right atrial pressure/CO), a measure of resistance to blood flow through the pulmonary vascular bed. A reduction in PVR in PAH is a beneficial change.

The use of implanted PAP sensors permits daily measurements of mPAP and the calculation of CO using a proprietary algorithm. mPAP divided by CO provides a value for total pulmonary resistance (TPR), another measure of resistance to blood flow through the pulmonary circulation. The availability of daily measurements enables the change in TPR to be calculated over time, rather than just at the end of the study. A reduction in TPR can be considered an indication of therapeutic efficacy. Furthermore, the longitudinal data series for each patient can provide insight of the time course of response.

In this study, we will include analysis of continuously gathered cardiopulmonary haemodynamic (mPAP, CO, heart rate) and physical activity data gathered from the FDA/CE-approved pulmonary artery pressure monitors (CardioMEMS, Abbott Laboratories) and insertable cardiac monitors (LinQ, Medtronic) and remote measurement of blood pressure, weight and O2 saturations (CHFS, Endotronix) to compliment the primary objective of identifying the highest best tolerated dose. We will also analyse changes in WHO functional class, 6 minute walk distance and plasma NTproBNP.

**Available data**

***Baseline and repeated at follow up***

We will tabulate the following reported measures at baseline and follow up: Body mass index, systemic and pulmonary blood pressure – both diastolic (mm Hg) and systolic (mm Hg), total pulmonary resistance (Wood units), mean pulmonary artery pressure (mmHg), cardiac output (L/min),

cardiac index (L/min/m$^2$), functional class (expressed as a proportion of either 1, 2, 3, 4), NTproBNP (ng/L), 6 minute walk distance and background drug therapy (endothelin receptor antagonist, phosphodiesterase type 5 inhibitor, soluble guanylate cyclase stimulator, and prostanoid oral, nebulised or iv).

***Daily, time-varying measures collected from the heart monitors, during the 24-week follow-up.***

The following will be collected or calculated from daily measurements: Mean PAP (measured), CO (derived from algorithm), TPR (mPAP/CO), activity of daily living (minutes per day) and heart rate (beats per minute).

**Analysis plan**

***Changes in endpoint efficacy measures***

Change in baseline and endpoint efficacy measures will be compared by paired Students t-test or Wilcoxon test as appropriate.

***Changes in TPR, total systemic resistance, heart rate and physical activity***

Change in TPR will be calculated from invasive right heart catheterisation (snapshot at baseline and 24 week follow up) or pulmonary artery pressure monitor (average of the 14-days preceding drug administration or withdrawal) derived measures as described above (mPAP/CO). For patients with implanted devices, time to stability of TPR following drug initiation and withdrawal will be evaluated using time-to-event analysis with an event defined as a change in 3-day average of TPR of less than 0.15 Wood Units for 6 of 7 consecutive days. Likewise changes in heart rate and activity will also be based on the change in the 3-day average.

***Pharmacokinetic analyses***

Pharmacokinetic analysis will be performed using nonlinear mixed effects modeling (NONMEM version 7.3).
